# Abstracts of the 2024 50th Annual NATAS Conference

**DOI:** 10.3390/polym16192844

**Published:** 2024-10-08

**Authors:** Rigoberto Advincula, Christopher Bowman, Dennis Smith, Karl Schoch, Tina Adams, Cathy Stewart, Lawrence Judovits, Louis Pilato

**Affiliations:** 1 Knoxville/Oak Ridge National Laboratory, University of Tennessee, Knoxville, TN 37830, USA; 2 University of Colorado, Boulder, CO 80309, USA; christopher.bowman@colorado.edu; 3 Mississippi State University, Starkville, MS 39762, USA; 4 Northrop Grumman Systems Corporation, Linthicum, MD 21090-2202, USA; k.eric.schoch@ngc.com; 5 Lubrizol, Wickliffe, OH 44092, USA; 6 Intertape Polymer Group, Marysville, MI 48040, USA; 7 Arkema (retired), King of Prussia, PA 19406, USA; 8 Pilato Consulting, Bound Brook, NJ 08805, USA; lapilato@optonline.net

**Keywords:** thermal analysis

## Abstract

The 50th annual conference of the North American Thermal Analysis Society is being held jointly with the 8th Baekeland Symposium to bring those communities together. NATAS offers scientists and practitioners the opportunity to explore the frontiers of thermal analysis, rheology, and materials characterization, learn of developments in instrumentation and software, as well as applications in a variety of industrial settings. The Baekeland Symposium has a record of showcasing the latest scientific, technical and industrial innovations in the field of high performance thermosetting polymers. This year is the first joint meeting of the two organizations and is expected to provide opportunities for new collaborations and exchange of ideas. Presentations and posters by renowned scientists and graduate students set the stage for excellent discussions and an ideal environment to learn about state-of-the-art techniques and exciting new developments in thermal analysis and materials research.

## 1. Plenary Lectures

### 1.1. Mettler-Toledo Award Presentation: Aspects of the Calorimetric Characterization of Matter in the Non-Equilibrated State

**Jürgen E.** **K. Schawe**
ETH Zurich
*  Correspondence: juergen.schawe@mat.ethz.ch

**Abstract:** The characterization of the formation of metastable structures and the kinetics of the associated transformations is an important task in materials research and modern calorimetry. Many improvements in dynamic scanning calorimetry (DSC) have been developed for this task over the last 35 years. These include the development of databases of materials, software for the correction and evaluation of measurement curves, software tools for kinetic evaluation [1], new DSC techniques such as temperature-modulated DSC [2] and the improvement of conventional DSC devices as well as the development of calorimetric techniques such as Fast DSC (FDSC) with MEMS chip technology [3,4]. From this large number of developments, I would like to focus on the techniques in which I was more or less involved. These are the introduction of complex heat capacity in DSC [5], the advanced modulation technique called TOPEM [6], and FDSC [7]. These techniques are used for various thermal events in metastable materials such as glass transition, crystallization, solid state transitions and melting. However, I will also show the determination of the thermodynamic properties of metastable phases.

**References**

1. Vyazovkin, S. Modification of the integral isoconversional method to account for variation in the activation energy. *J. Comp. Chem*. **2001**, *22*, 178–183.
2. Reading, M.; Elliott, D.; Hill, V.L. A new approach to the calorimetric investigation of physical and chemical transitions. *J. Therm. Anal.* **1993**, *40*, 949–955.
3. Zhuravlev, E.; Schick, C. Fast scanning power compensated differential scanning nano-calorimeter: 1. The device. *Thermochim. Acta* **2010**, *505*, 1–13.
4. Mathot, V.; Pyda, M.; Pijpers, T.; Poel, G.V.; van de Kerkhof, E.; van Herwaarden, S.; van Herwaarden, F.; Leenaers, A. The Flash DSC 1, a power compensation twin-type, chip-based fast scanning calorimeter (FSC): First findings on polymers. *Thermochim. Acta* **2011**, *522*, 36–45.
5. Schawe, J.E.K. Principles for the interpretation of modulated temperature DSC measurements. Part 1. Glass transition. *Thermochim. Acta* **1995**, *261*, 183–194.
6. Schawe, J.E.K.; Hütter, T.; Heitz, C.; Alig, I.; Lellinger, D. Stochastic temperature modulation: A new technique in temperature-modulated DSC. *Thermochim. Acta* **2006**, *446*, 147–155.
7. Schawe, J.E.K.; Löffler, J.F. Existence of multiple critical cooling rates which generate different types of monolithic metallic glasses. *Nat. Commun*. **2019**, *10*, 1337.

### 1.2. Net-Zero by 2050: Can We Get There?

**David Sholl**
Oak Ridge National Laboratory
*  Correspondence: shollds@ornl.gov

**Abstract:** The US and many other countries have embraced goals of reaching net-zero GHG emissions by 2050. I will discuss the far-reaching implications of this goal for the nation’s economy, and examine the urgent need to develop scalable net-negative technologies that can be deployed in the next decade. I will also discuss the roles for materials R&D in developing scalable technologies to achieve decarbonization goals on climate-relevant scales.

### 1.3. Cool Plastics for a Greener World

**Natalie Stingelin**
Georgia Institute of Technology
*  Correspondence: natalie.stingelin@mse.gatech.edu

**Abstract:** With seabirds trapped in multipack drink rings, and mid-ocean islands of indestructible rubbish, the idea that plastics could play a big part in a sustainable future world might seem far-fetched. However, new smart plastics may yet rescue the reputation of this all-consuming 20th century material. Research into “cool plastics” for cars and buildings could reduce the need for air conditioning and, thus, improve their energy efficiency. We will present recent efforts to design plastics of desired optical functions targeted for a greener world. We will discuss the potential of such systems that can offer the same flexibility, softness and light weight as commodity plastics but can control the flow of light and heat therefore assisting energy management in buildings and greenhouses in the form of heat mirrors, photovoltaic applications when used as anti-reflection coatings and semi-transparent mirrors, as well as building blocks for novel optical structures that can lead to quantum devices.

## 2. Session: Past Recipients of the Mettler-Toledo Award

### 2.1. My Journey with Thermal Analysis

**R. Bruce Prime**
IBM (retired)
*  Correspondence: rbprime@sbcglobal.net

**Abstract:** For me thermal analysis provided a highly versatile tool box that allowed me to address a vast number of applications and problems, starting in graduate school, during my 30-year career at IBM and into my retirement years.

Like many, my journey was influenced by teachers and mentors along the way, starting with my freshman chemistry teacher. A summer job at a polyurethane foam company introduced me to polymers, with RPI my next stop. It was my great good fortune to work with Professor Bernhard Wunderlich. He had discovered that linear polyethylene could form highly crystalline extended chain structures when cooled under high pressure. Together, we decided that “The Equilibrium Melting of Homopolymers” would be a good research topic for my PhD, which included modeling based on Flory-Huggins, HDPE, fractionation, GPC, density gradient columns, DSC and dilatometry.

When I joined IBM in Endicott, NY my experience with DSC led to me being put in charge of thermal analysis. While polyethylene was used at IBM epoxies were more prevalent. Studies with my colleagues led to my first publications on thermoset cure. After three years I joined the new Materials Science Complex in San Jose, CA that incorporated scientists and engineers from the operating divisions into the Research environment. TMA became central to my research into low expansion materials and creep compliance of toner in the hot roll fusing process. Next was a position in an advanced development group for an electrophotographic printer. In addition to toner a key polymeric material was the thin silicone rubber coating of the fuser roll, which operated in a high temperature, high humidity environment. TGA played a key role in both optimizing the cure and maximizing the life of the silicone rubber roller. Following the successful release of this product I joined an advanced development group for hard disk drives. DMA, TGA and TGA-MS played key roles in optimizing the cure and durability of the magnetic coating, a complex mixture including epoxy, phenolic, and Fe_2_O_3_. When the disk coating changed to metal thin film, the Materials Analysis Lab was next, where all of the thermal analysis techniques played important roles.

My “retirement” years have included consulting, teaching of short courses, and writing, all of which have involved thermal analysis. The Golden Gate Polymer Forum, a group I helped to form in 1980, has hosted several symposia and short courses where thermal analysis was a central theme, including a joint symposium with NATAS in 2003. My writing includes the chapter on “Thermosets” in Edith Turi’s book Thermal Characterization of Polymeric Materials, Thermal Analysis of Polymers with Joe Menczel (2nd edition with Larry Judovits in preparation), and a book on Thermosets with Jeff Gotro that is in preparation.

As to the future I see continuing advances in computing capability, in particular in the area of kinetics analysis. I believe that data from DSC measurements contain valuable kinetic information and that sophisticate kinetics software is becoming increasingly capable of extracting that information.

### 2.2. Accidental Discoveries/Innovations/Business Growth via Thermal Analysis & Rheology

**Yash Khanna**
InnoPlast Solutions
*  Correspondence: yash.khanna@innoplastsolutions.com

**Abstract:** Thermal Analysis and Rheology are uniquely positioned to probe into Structure-Property-Performance relationships and, more importantly, present opportunities for Accidental Discoveries, Innovations and Business Growth. In order to illustrate our claims, we will just present 3-case studies. (1) Discovery of Reactor Memory in Nylons after 50 years of their existence (2) Invention of High-Melting Teflon after 50 years of its discovery by DuPont and (3) Bringing Rheology into Thermal Analysis triggered by accidental melting of polyethylene in DMA. In addition, we will highlight the skills required to interpret DSC by presenting a Case-Study where a mis-interpretation of “ Nylon Crystallinity by DSC” led to a patent infringement between Honeywell (then AlliedSignal) and DuPont costing $50 MM.

The presentation will conclude with 7-Principles for promoting accidental discoveries, more frequently.

### 2.3. Composites, Rubber, Glasses and DNA: 50+ Years of Interrogating Materials Physics with Thermal Analysis and Rheology

**Gregory McKenna**
North Carolina State University
*  Correspondence: gbmckenn@ncsu.edu

**Abstract:** Experimentalists have a plethora of methods to choose from for their explorations of materials. For the past 50+ years I have been able to exploit a subset of such tools to interrogate the physics of materials at scales from the macroscopic to the nanometric and molecular. Rheological and mechanical methods complemented by calorimetry have served as the mainstays of such studies. In the beginning the work investigated how composite materials interacted with the bone remodeling process to develop orthopedic implant devices; Non-linear rheology was used in the first characterizations of the normal forces in glassy polymers and these methods were extended to investigate the strain energy density function of rubber networks and, when combined with swelling measurements, demonstrated that the Flory-Rehner swelling model at its deepest level is the correct thermodynamic description of rubber behavior. The work morphed into a series of investigations related to nonlinear mechanical spectroscopies that fingerprint heterogeneous material behaviors. DSC entered the repertoire in studies of the impact of nano-confinement on crystallization and the glass transition of organic molecules; Follow-on development of a nanobubble inflation method to measure the viscoelasticity of ultrathin polymer films led to important findings about glassy dynamics at the nanometer size-scale. The advent of Flash DSC complemented the rheology with measurement of temperature and rate dependence of the glassy response in ultrathin polymer films. Molecular rheology from rings to bottle brushes and DNA has given important insights into structure-property relationships for chain dynamics; Finally, a combination of the nanocalorimetry and nanomechanical measurements led to unique measurements of ultrastable glasses formed by vapor deposition methods that challenge the meaning of the Kauzmann paradox in the fundamentals of glass-formation and the glass transition. The journey has been exciting and continues to provide intriguing questions and answers to important problems in materials physics.

**Acknowledgments:** The National Science Foundation, the U.S. Army Research Office, and the Department of Energy Office of Science are thanked for their support of the work described in this abstract. The work was carried out at the University of Utah, the National Bureau of Standards/National Institute of Standards and Technology, the Institut Charles Sadron in Strasbourg, FR, the École Supérieure de Physique et de Chimie Industrielles de la Ville de Paris, Texas Tech University, and North Carolina State University.

### 2.4. A Life in Thermal Analysis: The Rigid Amorphous Phase, the Cooling Calibration of DSCs, Polymeric Fibers and the Crystal-to-Crystal Transitions

**Joseph Menczel**
Thermal Measurements, LLC
*  Correspondence: jmenczel@yahoo.com

**Abstract:** My name is Joseph D. Menczel, and I learnt the basics of thermal analysis from the person, who often was called the king of thermal analysis, Bernhard Wunderlich. Originally a physical chemist dealing with magnetic resonance, mostly with electron spin resonance (ESR or EPR), I started to do differential scanning calorimetry (DSC) in 1978 at the Technical University of Budapest, then I spent 1979 and 1980 at Rensselaer Polytechnic Institute (RPI) in Wunderlich’s lab, where I was doing DSC of liquid crystalline polymers. Through this topic, since of the major components of the earlier liquid crystalline polymer was PET we found the heat capacity change deficiency for semicrystalline polymers first for PET and then for most semicrystalline polymers (Menczel, J., and Wunderlich, B. (1980) J. Polym. Sci., Polym. Lett. Ed. 19, 261) called later ”rigid amorphous fraction (RAF)” It took a long time to clarify the nature of the RAF. I reported the most important characteristics of the RAF, the correlation with the chain rigidity just last year here at the NATAS conference. For the RAF I received the Mettler-Toledo Award in 2010. In addition, among other things, I dealt with the mechanism of crystal-to-crystal transitions of polymers, developed the cooling calibration of DSCs. Also, I am the author of the Fibers and Films chapter in the Turi book, published three other thermal analysis books, and working on two more books. For a while I was the North American editor of JTAC, but I had to give this up, a lot of time is needed for writing books. 2009–2013 I was the Secretary of NATAS, in 2013 I was the President of NATAS.

### 2.5. Sixty Years of Thermal Analysis and Counting

**Andrew McGhie**
Laboratory for Research on the Structure of Matter, University of Pennsylvania
*  Correspondence: mcghie@lrsm.upenn.edu

**Abstract:** This talk will be unlike any other and will cover a journey from birth in the most blitz-ravaged town in Scotland during WWII to the birthplace of the United States and where I found thermal analysis. It is a life in which thermal analysis played a leading, supportive role in the broader field of materials research and education. It is a life that was enriched by membership and participation in three societies ranging from the local, through national, to the global: TAFDV, NATAS, and ICTAC, and which continue to stimulate me to this day. Research and development, educational outreach, and helping others has made the journey so much fun, especially when punctured sporadically by the arts.

### 2.6. Milestones with Thermal Analysis: Mode of Degradation for Vinylidene Chloride Barrier Polymers and Poly(Styrene)

**Bob A. Howell**
Central Michigan University
*  Correspondence: bob.a.howell@cmich.edu

**Abstract:** The thermal degradation processes for two important commercial polymers, vinylidene chloride barrier polymers and poly(styrene), have been established using thermogravimetry as a primary technique coupled with evolved gas analysis and appropriate spectroscopic methods. Vinylidene chloride polymers display very low permeability to the transport of small molecules, most notably oxygen. This property has given them a premier position as materials for plastic packaging. However, these materials undergo thermal degradation at relatively low temperature. Special techniques must be used for processing to avoid degradative damage. The degradation may most conveniently be observed using thermogravimetry. The degradation reflects sequential dehydrochlorination along the polymer mainchain via a tight radical pair. Poly(styrene) is a large volume commodity polymer which finds applications in many areas, including packaging. Thermal degradation may occur under relatively mild conditions and is strongly temperature dependent. At low temperature, 2800 °C, cleavage of the mainchain at a head-to-head unit, introduced by polymerization termination by radical coupling, occurs. The macroradicals formed rapidly unzip to expel styrene monomer. At higher temperatures several processes become significant.

### 2.7. Thermal Properties of Silk Fibroin Protein

**Peggy Cebe**
Tufts University
*  Correspondence: peggy.cebe@tufts.edu

**Abstract:** In 2013 I was honored to receive the Metter-Toledo Award in Thermal Analysis. My lecture that year centered on thermal properties of fibroin, the protein found in silk fibers, using primarily differential scanning calorimetry and the then-novel approach of fast scanning calorimetry. Collaborating with the group of Christoph Schick, we used FSC to demonstrate the melting of dry (i.e., non-solvated) beta pleated-sheet crystals of fibroin, the first time that thermally induced crystal melting was reported for any fibrous protein. Later on, after first commercialization of FSC as the Mettler Flash DSC1, a series of experiments was undertaken to distinguish the melting of different crystalline polymorphs of silk fibroin. In my presentation, I will review the challenges faced when working with fibrous proteins and compare the thermal behavior of silk fibroin to that of synthetic semicrystalline polymers. My collaborators on various aspects of this work include: Xiao Hu, Benjamin Partlow, David Kaplan, David Thomas, John Merfeld, Marek Pyda, Rufina Alamo, Evgeny Zhuravlev, Andreas Wurm, Daniella Arbeiter, and Christoph Schick

### 2.8. Tg and Structural Recovery by Flash DSC

**Sindee Simon**
North Carolina State University
*  Correspondence: slsimon@ncsu.edu

**Abstract:** The behavior of glass-forming materials has been of considerable interest over the past half century with a number of unresolved issues and debates. In this talk, we will examine what we have learned about the glass transition and new experiments that we have been able to accomplish using the Mettler Toledo Flash differential scanning calorimeter (DSC). The advantages of the Flash DSC include sufficient sensitivity to measure enthalpy recovery for nanogram samples, as well as extension of the measurements to aging times as short as 0.01 s and to aging temperatures as high as 15 K above nominal Tg since high fictive-temperature glass can be created by the fast cooling rates (1000 K/s). The discussion will include the behavior of bulk polymeric samples, as well as samples in nanoconfined and other geometries.

### 2.9. Defining Starch Materials: Structure and Properties

**Janis Matisons**
Intertape Polymer Group
*  Correspondence: jgmatisons@gmail.com

**Abstract:** Starch is one of the three most abundant and renewable biopolymers on earth (cellulose and chitin are the other two). In food science starch remains the most used polymer. In botany, starch has three tree structural motifs, based on crystalline morphology {corn is A type; Potato is B type and Peas are C type), which will be explained in my talk. Processing starch in water breaks this crystalline morphology, and processing such a solution through drying can develop an alternative crystalline morphology (Vh). we see this in bread staling over time. In industrial use, such changes must be carefully monitored as properties change. Thermal analysis has generally been one of the principal techniques used to monitor such changes and transformations. Industrial starch formulations incorporate additives, generating complex morphologies with synergistic properties. I will briefly review the complex interactions of such starch formulations and discuss the challenges that still lie ahead.

### 2.10. TA Today the Impact of Thermal Analysis for the Characterization of Bioderived and Bioinspired Polymeric Materials Sustainable Materials, Materials for Use In Vivo

**Michael Jaffe**
New Jersey Innovation Institute
*  Correspondence: jaffe@njit.edu

**Abstract:** Over my decades long career, I have seen the focus of polymer research shift from understanding basic polymer science (morphology, equilibrium melting point) to polymer process and product development (fibers, films, plastics) to the present focus on bioderived and bioinspired materials (renewable polymers to replace petroleum-based plastics, biocompatible polymers for in-vivo medical use). Thermal Analysis remains an effective tool for the characterization and understanding of these materials, allowing the integration of physical science and biological concepts to create effective solutions to current problems. To illustrate the utility of thermal analysis in understanding these materials I review the use of isosorbide, a corn starch (glucose derived) bioderived diol as an effective chemistry for improving, rather than just replacing, historically petroleum-based materials. The combinatorial library of tyrosine based pseudo amino acid polymers invented by Professor Joachim Kohn for in-vivo use is my example of the power of TA in understanding the complex behavior of bioinspired materials. While the focus of the work may change, the rules and underlying science does not, all that changes are the independent variables.

## 3. Session: Energetic Materials and Thermal Hazards

### 3.1. Novel Screening Method for Explosive Hazard Identification in Pharmaceutical Process Development

**Han Xia**
Eli Lilly and Co.
*  Correspondence: hxia@lilly.com

**Abstract:** As a pharmaceutical company, Lilly seeks to avoid the use or production of explosive materials. However, Lilly does use some reagents and intermediates which could potentially be classified as explosives based on their chemical structure. Under the current UN standard, Lilly has a legal obligation to ship a minimum of 1.5 kg of material to a designated UN explosive shooting range to be properly classified. This sample size is unrealistic for Lilly’s early-stage portfolio, and a late stage project might face delays if an intermediate or reagent is determined to be an explosive at a later stage. The ability to reduce this risk at an earlier stage with small amounts of material is important to the success of the business and the safety of its employees. The goal of this project was to develop a smaller scale screening method that requires approximately 1 g of material. This was done by testing a set of explosive compounds, non-explosive compounds, and unclassified compounds with both differential scanning calorimetry and accelerating rate calorimetry. Parameters affecting the explosivity of a compound were collected from these tests and the structures of the compound. JMP software’s neural network was then utilized to analyze this dataset and predict the hazard classification of each unknown compound. Classifications were verified with the lab lead and added to the training set of the neural network to enable more accurate predictions of future potential explosives.

### 3.2. Penetration Depth Test Study of Pellet to Cladding Interaction in Fast Reactor Design

**Yan Peng**
China Institute of Atomic Energy
*  Correspondence: pengyan@ciae.ac.cn

**Abstract:** CFR600 is a pool-type sodium-cooled fast reactor. Its thermal power is about 1500 MW and its electrical power is about 600 MW. The fuel material is MOX of 30 wt% PuO_2_ with 70 wt% UO_2_, and the cladding 15–20%CW 15-15Ti. In order to gain a right design which can be adopted into the real engineering application, that is required to study Pellet to Cladding Interaction (PCI) phenomenon which is the important project fort he safety review in Fast Reactor design.

This paper focuses on the inter-diffusional factors of diffusion coupling problem as PCI relates to the fuel and the cladding operation at elevated temperatures [1]. At locations along the interface between the fuel and the cladding where there is good contact, inter-diffusion can occur among fuel, fission product and cladding constituents. This fuel-cladding chemical interaction should lead to the development of interaction zones along the inner surface of the cladding, which can input the performance of a fuel element! Therefore, the metallurgical phase diagrams of these diffusion coupling zones come first [2], for analyzing their physical property changes with the increasing of NPP burn-ups.

This paper creatively merges the ASTM E37 Standards on Thermal Measurements, E228 [3] and E831 [4], into the research about the penetration depth of this kind of interaction zones or diffusion couplings during PCI. Furthermore, it highlights that these diffusion coupling zones can be brittle, causing the cracks that could affect the mechanical integrity of the claddding to be initiated, and especially, they can contain relatively low-melting phases.

**References**

1. Castleman, L.S. An analytical approach to the diffusion bonding problem. *Nucl. Sci. Eng. ANS* **1958**, *4*, 209–226.
2. Boettinger, W.J.; Kattner, U.R.; Perepezko, J.H. *DTA and Heat-Flux Dsc Measurements of Alloy Melting and Freezing*; Special Publication 960-15; National Institute of Standards and Technology: Gaithersburg, MD, USA, 2006.
3. *ASTM E228*; Standard Test Method for Linear Thermal Expansion of Solid Materials with a Pushrod Dilatometer. Annual Book of ASTM Standards: West Conshohocken, PA, USA, 2015; Volume 14.02, pp. 61–69.
4. *ASTM E831*; Standard Test Method for Linear Thermal Expansion of Solid Materials by Thermomechanical Analysis. Annual Book of ASTM Standards: West Conshohocken, PA, USA, 2015; Volume 14.02, pp. 237–240.

### 3.3. An Improved Chemical Kinetic Model for TATB Decomposition

Alan Burnham  ^1^^,^*, Jason Moore  ^2^, Batikan Koroglu   **^2^, Keith Morrison**  **^2^, Keith Coffee**   **^2^, Greg Klunder**   **^2^, Adele Panasci-Nott**  **^2^, Ana Racoveanu**  **^2^** **and John Reynolds**   **^2^**
^1^  Stratify//MH Chew Associates
^2^  Lawrence Livermore National Laboratory
*  Correspondence: burnham1@llnl.gov

**Abstract:** Thermal analysis (TG and DSC), analytical pyrolysis (Py-MS, Py-GCMS, and Py-FTIR), bulk pyrolysis, FTIR characterization of residues, and traditional extraction and chromatography methods (LC-DAD and LC-MS) have been combined to create a detailed model of chemical species creation and destruction [1–5]. Isotopic substitution was important to confirm some reaction pathways. No one technique was sufficient to develop an adequate picture of the lumped-species decomposition mechanism, which includes two main competitive pathways, each having multiple sequential steps. This talk emphases the strengths and weaknesses of thermal analysis methods contributing to the overall picture. Autocatalysis is prominent in the reaction network, as was characterized using interrupted-heating DSC experiments. Commonly used methods for analyzing TG and DSC data give misleading chemical kinetic parameters, although they are helpful for understanding the full picture. One limitation is that kinetics derived at relatively fast heating rates underestimate autocatalysis for long thermal soaks at low temperatures. Another limitation is that the extent of conversion can depend on heating rate, sample size, and confinement conditions. Not having the same conversion for all experiments means that the isoconversional principle is not honored, which invalidates some methods. The ultimate optimization of the kinetic parameters was accomplished using coupled heat and mass transport equations in combination with the chemical kinetic network to model cookoff at the scale of grams [6].

**References**

1. Koroglu, B.; Burnham, A.K.; Mashiana, I.; Racoveanu, A.; Arose, C.; Crowhurst, J.C.; Reynolds, J.G. Comparative analysis of sublimation and thermal decomposition of TATB. *Propellants Explos. Pyrotech.* **2024**, *49*, e202300124. https://doi.org/10.1002/prep.202300124.
2. Morrison, K.D.; Moore, J.S.; Coffee, K.R.; Koroglu, B.; Burnham, A.K.; Reynolds, J.G. TATB thermal decomposition: Expanding the molecular profile with cryo-focused pyrolysis GC-MS. *Propellants Explos. Pyrotech.* **2024**, *49*, e202300268. https://doi.org/10.1002/prep.202300268.
3. Burnham, A.K.; Coffee, K.R.; Klunder, G.L.; Panasci-Nott, A.F.; Reynolds, J.G. Towards a heat- and mass-balanced kinetic model of TATB decomposition. *Propellants Explos. Pyrotech.* **2024**, *49*, e202300121. https://doi.org/10.1002/prep.202300121.
4. Coffee, K.R.; Panasci-Nott, A.F.; Olivas, J.A.; Selinsky, J.; Morrison, K.D.; Burnham, A.K.; Klunder, G.L.; Reynolds, J.G. Analysis of degradation products in thermally treated TATB. *Propellants Explos. Pyrotech.* **2024**, *49*, e202300176. https://doi.org/10.1002/prep.202300176.
5. Moore, J.S.; Morrison, K.D.; Burnham, A.K.; Racoveanu, A.; Reynolds, J.G.; Koroglu, B.; Coffee, K.R.; Klunder, G.L. TATB thermal decomposition: An improved kinetic model for explosive safety analysis. *Propellants Explos. Pyrotech.* **2024**, *49*, e202300237. https://doi.org/10.1002/prep.202300237.

This work was performed under the auspices of the U.S. DOE by Lawrence Livermore National Laboratory under contract DE-AC52-07NA27344. LLNL-ABS-862220.

### 3.4. Influence of the Temperature and Size on the Safety Conditions for Reactions in Big Volumes

**Elena Moukhina**
NETZSCH Geraetebau GmbH
*  Correspondence: elena.moukhina@netzsch.com

**Abstract:** It is known that the rate of exothermal reactions depends on the temperature and changes with the time depending on reaction type. However, for the big volumes the size and geometry of reacting material become to have the high influence on the safe storage and safe industrial processes. For the reactions with the high energetic potential the temperature gradients and hot spots can lead to runaway reactions and thermal explosion. If the reacting material is placed into container with on-zero mass and heat capacity, then it can get the part of the heat producing during reaction and therefore the reaction rate and temperature increase will be different from those in the material without container. The additional factors are stirring or active surficial cooling. Depending on these factors the storage of material packages can be safe or not. For industrial processes these factors influence on the fact, if only primary reaction starts or both primary and secondary reactions will run.

Present work contains the simulations for different size and temperature conditions, with the goal to find the critical conditions leading to runaway reactions and for triggering of the secondary reaction.

It is shown that the classical calculation according to Phi-factor is often not conservative enough. The results present the examples where time to maximum rate (TMR) is less, and the maximal temperature is higher than the values according the classical approach over phi-factor.

The simulations are done with NETZSCH Termica Neo software, which is fully compatible with Kinetics Neo Software which could be used for analysis of thermal analytical data like DSC, TG or ARC.

### 3.5. Heat Flow Calorimetry on High Explosives at Low Temperatures

John Rosener *, Cody Cockreham, Elizabeth Glascoe and Steven Hawks
Lawrence Livermore National Laboratory
*  Correspondence: rosener1@llnl.gov

**Abstract:** Characterizing the thermal properties of high explosives is a key component to a comprehensive view of explosive safety. Thermal properties of interest are typically: degradation onset temperature, thermal runaway temperature, the amount of heat released upon thermal decomposition, etc. Measuring these properties on big and bulky explosive samples is a challenge for several reasons. The main challenge being risk mitigation to personnel and infrastructure. If a large explosive sample unintentionally detonates during a test, people and buildings are at risk of irreparable damage. To minimize this risk, explosive sample weights are reduced to the absolute minimum amount required.

A standard test method to probe the thermal properties of small amounts of high explosive is differential scanning calorimetry (DSC). In a DSC test, high explosives are heated at a constant heating rate from an initial temperature to a final temperature. DSC by design is meant to be a relatively quick test due to the use of a fixed heating rate (10 °C/min, 5 °C/min, etc.). However, when studying slow decomposition mechanisms of high explosives at low temperatures (less than 150 °C), the DSC instrument may not be sensitive enough to detect it. When heating high explosives at typical heating rates, by the time you detect a change in the heat flow signal, you are not measuring a single decomposition mechanism but several happening at the same time. DSC instruments have the capability of performing isothermal hold segments at low temperatures. However, the baseline heat flow signal will begin to drift if the isothermal hold segment is too long. At that point, you might not be sure whether the change you are seeing is due to the sample or due to measurement drift.

The sensitivity limitations of DSC for slow reactions at low temperatures has led to an effort to find alternative testing methods. Heat Flow Calorimetry (HFC) is a technique that could fill this gap for high explosives. HFC instruments are designed to measure heat flow at a constant temperature and do so for long periods of time. Published HFC literature has demonstrated experiments that have run from weeks to months in duration. HFC is a documented method for testing the stability of propellants from room temperature up to 90 °C. Not a lot of literature has been published on HFC used on high explosives. This talk will focus on the use of HFC with respect to high explosives, and the different information that can be gathered using it.

This work was performed under the auspices of the U.S. Department of Energy by Lawrence Livermore National Laboratory under Contract DE-AC52-07NA27344.

### 3.6. Thermoanalytical Studies on the Reactions of Magnesium and Lithium Powders with Oxygen and Carbon Dioxide

Sergio Cordova, Kevin Estala-Rodriguez and Evgeny Shafirovich *
The University of Texas at El Paso
*  Correspondence: eshafirovich2@utep.edu

**Abstract:** Knowledge of the reactions of magnesium and lithium particles with oxygen and carbon dioxide is required for the development of space power systems based on combustion of metals [1,2]. However, the kinetics of these reactions are not well understood. Here we present the results of our thermoanalytical studies on the reactions of magnesium powder and stabilized lithium metal powder (SLMP) with O_2_ and CO_2_ gases [3–5]. Both isothermal and non-isothermal methods of thermal analysis (TGA and DSC) were used. For spherical Mg particles heated in an O_2_ flow, the Avrami-Erofeev model of simultaneous nucleation and growth provides the best fit with the experimental isothermal and non-isothermal TGA curves. The Mampel-Delmon analysis of the isothermal TGA data has shown that for smaller (30 to 60 µm) particles, the nucleation is relatively slow, leading to the sigmoidal TGA curve, described by the three-dimensional Avrami-Erofeev equation. An increase in the particle size decreases the dimension of this equation, and for larger (300 µm) spherical particles, as well as for flakes, the entire process can be considered as a growth of a non-protective oxide layer. For Li particles, the TGA in O_2_/Ar environments has revealed the formation of lithium peroxide (Li_2_O_2_) in addition to lithium monoxide (Li_2_O) at temperatures below 400 °C. At higher temperatures, Li_2_O_2_ decomposes, which explains the observed acceleration of the reaction. For Mg particles in CO_2_ and Li particles in both O_2_ and CO_2_, a noticeable reaction starts after melting of the metal and leads to the formation of a hollow shell, which indicates that the oxidation process includes growth of a solid oxide layer on the surface of the metal droplet and simultaneous growth of a cavity inside the droplet. The reaction of Li particles with CO_2_ includes formation of Li_2_O and its subsequent conversion into lithium carbonate (Li_2_CO_3_). High-pressure DSC has shown that with increasing pressure, the conversion of Li_2_O into Li_2_CO_3_ occurs at a lower temperature.

**References**

1. Shafirovich, E. Lithium and magnesium fuels for space propulsion and power. In *Nano- and Micro-Scale Energetic Materials*; DeLuca, L.T., Pang, W.Q., Eds.; Propellants and Explosives; Wiley-VCH: Weinheim, Germany; 2023; pp. 397–416.
2. Shafirovich, E. Conceptual design of a space power system based on combustion of metals. *J. Propuls. Power* **2023**, *39*, 464–468.
3. Cordova, S.; Estala-Rodriguez, K.; Shafirovich, E. Oxidation kinetics of magnesium particles determined by isothermal and non-isothermal methods of thermogravimetric analysis. *Combust. Flame* **2022**, *237*, 111861.
4. Estala-Rodriguez, K.; Cordova, S.; Shafirovich, E. Oxidation and combustion of stabilized lithium metal powder (SLMP). *Proc. Combust. Inst.* **2023**, *39*, 3583–3592.
5. Cordova, S.; Estala-Rodriguez, K.; Shafirovich, E. Oxidation and combustion of lithium and magnesium powders for space power applications. *Int. J. Energetic Mater. Chem. Propuls.* **2023**, *22*, 53–66.

### 3.7. A Safe Approach to Explosive Material Processing with LabRAM Technology

**Ginger Guillen**
Lawrence Livermore National Laboratory
*  Correspondence: guillen5@llnl.gov

**Abstract:** Modifying the particle size of synthesized high explosive (HE) materials is of great interest for changing the properties of new HE formulations and applications. Particle size reduction can play a crucial role in optimizing the performance and safety of explosives. Use of Resonant Acoustic Mixing (RAM) for processing HE materials has increased significantly over the past decade, with new efforts being focused on its ability to mill explosive materials. RAM offers an innovative approach that harnesses the power of acoustic energy, but at high accelerations and chaotic collision rates, we must consider a safe approach when processing explosive materials. In addition to reduced sensitivity of “some” conventional explosives, reducing heat generation (especially during scale-up operations) is another challenge we must continue to consider, ensuring a safe approach to material processing. By achieving precise and controlled particle size reduction, we can improve properties and enhance the performance of explosive materials.

### 3.8. New Kinetic Approach for Evaluation of Thermal Hazard Indicators Based on DSC and ARC Data

**Bertrand Roduit** **^1^** ^,^*, **Patrick Folly** **^2^, Alexandre Sarbach** **^2^** **and Richard Baltensperger** **^3^**
^1^  AKTS SA
^2^  armasuisse
^3^  Department of Safety, Health, and Environmental Engineering, University of Applied Sciences of Western Switzerland
*  Correspondence: b.roduit@akts.com

**Abstract:** The estimation of the hazard probability of “reactive or self-reactive” is a very important issue especially for the safety analysis of many technological processes or packaged materials during transport conditions [1,2]. The dangerous runaway scenario is quantitatively characterized by the thermal hazard indicators such as the Time to Maximum Rate under adiabatic conditions (TMRad) and the Self Accelerating Decomposition Temperature (SADT).

Due to the fact that significant amount of heat is evolved during the decomposition of self-reactive chemicals their thermal properties are frequently investigated in laboratory at mg- or g-scales under non-isothermal or isothermal conditions using Differential Scanning Calorimetry (DSC) or, more sensitive, Heat Flow Calorimetry (HFC) techniques [3–5].

The elaboration of the heat flow data monitored by these both techniques allows determination of the kinetic parameters of the decomposition process which describe the rate of the heat generation in the conditions of the ideal heat exchange with the surroundings. However, the correct scale-up of the results obtained by DSC or HFC requires the application of the proper heat balance in the simulated system. In kg-scale, due to increasing sample mass, the conditions of the heat exchange with the environment significantly changes what, in turn, may significantly increase the reaction rate and the spatial evolution of the sample temperature [3–5].

Presented study illustrates the basic principles of a new kinetic analysis workflow in which the heat flow traces collected in mg-scale (e.g., DSC, Calvet, TAM, etc.) are simultaneously considered with the results of investigations performed in accelerating rate calorimeters (ARC) [5]. Using the newly proposed kinetic workflow can improve the accuracy of scale-up experiments compared to using ARC data alone. This method improves the accuracy of simulations for large-scale experiments and helps to determine more accurate temperatures for explosions and thermal hazard indicators. It also significantly reduces the number of costly and time-consuming experiments required.

New kinetic approach is illustrated by the simulation of TMRad and SADT values of Azobisisobutyronitrile (AIBN), 3-Methyl-4-nitrophenol (3M4NP) and Di-tert-butyl peroxide (DTBP) in toluene using DSC, Calvet and TAM experiments with heat-wait-search ARC data.

**References**

1. Stoessel, F. Thermal safety of chemical processes: risk assessment and process design. Wiley-VCH Verlag GmbH & Co. KGaA; 2020.
2. Recommendations on the Transport of Dangerous Goods, Model Regulations, Vol. I, 21-th Revised Edition, United Nations. 2019. Available online: https://unece.org/fileadmin/DAM/trans/danger/publi/unrec/rev21/ST-SG-AC10-1r21e_Vol1_WEB.pdf (accessed on 27 December 2024).
3. Roduit, B.; Hartmann, M.; Folly, P.; Sarbach, A.; Baltensperger, R. *Thermochim. Acta* **2015**, *621*, 6.
4. Roduit, B.; Hartmann, M.; Folly, P.; Sarbach, A.; Brodard, P.; Baltensperger, R. *Chem. Ing. Trans*. **2016**, *48*, 37.
5. AKTS-Thermokinetics Software (Versions DSC-ARC and TS 6.02), AKTS SA, Technopôle 1, Sierre, Switzerland. Available online: https://www.akts.com (accessed on 17 May 2024).

### 3.9. Exothermic Decomposition Energy and Temperature—Proposal for Measurement Standardization in the United Nations Manual of Tests and Criteria

Shanti Singh *, Barbara Acheson, Garrison Condran and Sam Maach
Canadian Explosives Research Laboratory
*  Correspondence: shanti.singh@canada.ca

**Abstract:** Differential scanning calorimetry (DSC) is used as a screening test in the United Nations Manual of Tests and Criteria (UN-MTC, Appendix 6) to measure enthalpy (-∆H) and onset temperature (Tonset) of decomposition of a chemical. These data help determine whether a chemical can be excluded from being classified as a Class 1 (Explosive) material, provided that certain threshold values for -∆H and Tonset are not exceeded. However, variations in DSC testing protocols across different laboratories, such as the choice of sample vessel, sample mass, baseline shape, peak shape and heating rate, can impact the recorded -∆H and Tonset. To enhance the interlaboratory reproducibility of the DSC measurement, Appendix 6 would benefit from standardized guidelines. Working towards this goal, CERL is preparing samples for DSC using a consistent methodology for various chemicals, including dicumlyperoxide, dimethyldinitrobutane, ditertbutylperoxide, ammonium nitrate and pentaerythritoltetranitrate. Using the proposed methodology, preliminary results from CERL will demonstrate the consistency of data (-∆H, Tonset). Other laboratories are invited to take part in these DSC tests using the same chemicals and unified protocol, with all collected data available for review and comments.

© His Majesty the King in Right of Canada, as represented by the Minister of Natural Resources, 2024.

### 3.10. Thermal-Treated IrOx Electrodes with Boosted Efficiency and Durability for Green Hydrogen Production

**Jun Li** **^1^** ^,^* **, Feng-Yuan Zhang** **^1^** **, Weitian Wang** **^1^** **, Lei Ding** **^1^** **and David Cullen** **^2^**
^1^  University of Tennessee, Knoxville
^2^  Oak Ridge National Laboratory
*  Correspondence: fzhang@utk.edu

**Abstract:** Proton exchange membrane electrolyzer cells (PEMECs) have been regarded as a promising energy storage and conversion technology due to high efficiency, compact design, and close-to-zero emissions advantages [1]. The major challenges associated with oxygen evolution reactions (OERs) inhibits their large-scale application, including the high cost of electrodes and scarcity of Ir-based catalyst [2,3]. To manufacture the low-cost OER electrodes with boosted performance and durability, we electroplate the nanostructured ionomer-free IrOx catalysts onto titanium porous transport layers to form gas diffusion electrodes (GDEs). The GDE obtained by controlling the current density exhibits a low overpotential of 263 mV at 10 mA cm^−2^, indicating a high energy conversion efficiency. More importantly, it is found that the post thermal process can significantly improve the durability of IrOx electrodes. This work provides new insights into low-cost facile GDE fabrication for high-energy efficient PEMECs.

**References**

1. Ding, L.; Wang, W.; Xie, Z.; Aaron, D.S.; Paxson, A.; Hamdan, M.; Zhang, F.Y. Efficient Integrated Electrode Enables High-Current Operation and Excellent Stability for Green Hydrogen Production. *ACS Sustain. Chem. Eng.* **2023**, *11*, 11219–11228.
2. Ding, L.; Wang, W.; Xie, Z.; Li, K.; Yu, S.; Capuano, C.B.; Zhang, F.Y. Highly Porous Iridium Thin Electrodes with Low Loading and Improved Reaction Kinetics for Hydrogen Generation in PEM Electrolyzer Cells. *ACS Appl. Mater. Interfaces* **2023**, *15*, 24284–24295.
3. Xie, Z.; Ding, L.; Yu, S.; Wang, W.; Capuano, C.B.; Keane, A.; Zhang, F.Y. Ionomer-free nanoporous iridium nanosheet electrodes with boosted performance and catalyst utilization for high-efficiency water electrolyzers. *Appl. Catal. B Environ.* **2024**, *341*, 123298.

### 3.11. Boosting the Energy Conversion Efficiency of Power-to-Hydrogen via Developing a Novel Porous Transport Material

**Weitian Wang** * **, Jun Li, Lei Ding, Christopher Capuano, Nel Hydrogen; Kathy Ayers, Nel Hydrogen** **and Feng-Yuan Zhang**
University of Tennessee, Knoxville
*  Correspondence: wweitian@vols.utk.edu

**Abstract:** Power-to-hydrogen has been regarded as one of the most promising pathways to reach a carbon neutral world. However, the costs are still the main barrier for its large-scale commercialization, which can be significantly improved by the development of novel porous transport layer (PTL) materials. PTLs work as key water/gas transport components in the power-to-hydrogen devices, namely proton exchange membrane electrolyzer cells (PEMECs) [1,2]. In PTLs, the local gas accumulation has been widely recognized, which decreases the local water saturation and electrochemical reactions, and blocks some active areas [3]. Especially for some 2D structured PTLs, the absence of in-plane transport ability raises transport concerns at high current densities. In this study, a wet etching lithography method is introduced to fabricate 3D-structured PTLs, named flow-enhanced liquid/gas diffusion layers (LGDLs) for promoting multiphase transport. In-situ performance evaluation validates a 7.9% efficiency improvement at 6 A/cm^2^ compared with the 2D-structured LGDL in PEMECs, which are mainly attributed to the improved reactant supply to the pores under BP lands. Additionally, the mass transport limitation is extended from 6.3 to 9.0 A/cm^2^ with an anode water flow rate of 20 mL/min. By the in-situ visualization method, the enhanced in-plane mass transport is directly visualized. The successful fabrication and performance validation of flow-enhanced LGDL opened a new direction to develop high-efficiency power-to-hydrogen devices.

**References**

1. Kang, Z.; Mo, J.; Yang, G.; Retterer, S.T.; Cullen, D.A.; Toops, T.J.; Zhang, F.Y. Investigation Of Thin/Well-Tunable Liquid/Gas Diffusion Layers Exhibiting Superior Multifunctional Performance In Low-Temperature Electrolytic Water Splitting. *Energy Environ. Sci.* **2017**, *10*, 166–175.
2. Mo, J.; Kang, Z.; Retterer, S.T.; Cullen, D.A.; Toops, T.J.; Green, J.B., Jr.; Zhang, F.Y. Discovery of true electrochemical reactions for ultrahigh catalyst mass activity in water splitting. *Sci. Adv.* **2016**, *2*, e1600690.
3. Chen, Q.; Wang, Y.; Yang, F.; Xu, H. Two-dimensional multi-physics modeling of porous transport layer in polymer electrolyte membrane electrolyzer for water splitting. *Int. J. Hydrog. Energy* **2020**, *45*, 32984–32994.

### 3.12. Estimating the Post-Exotherm Pressure Generation from Heated High Explosives within a Sealed Vessel

Steven Hawks * and Maxwell Lynch
Lawrence Livermore National Laboratory
*  Correspondence: hawks3@llnl.gov

**Abstract:** High explosive (HE) aging experiments often involve heating a HE within a sealed container for an extended period of time. From a safety perspective, it is essential to know how much pressure could be generated in the event of unexpected thermal runaway. This information is used to design safe aging assemblies and scale HE quantities appropriately. Here we provide a simple method for calculating the maximum pressure rise after a complete thermal decomposition event. To validate the method, we employ a NETZSCH accelerating rate calorimeter (ARC) to directly measure pressure rise after thermal decomposition for several common high explosives and compare the results to theoretical predictions.

**Acknowledgments:** This work was performed under the auspices of the U.S. DOE by Lawrence Livermore National Laboratory under contract DE-AC52-07NA27344. LLNS, LLC.

### 3.13. Polymer Property Impacts on Processing Propellant Composites

Alexandra Dobbs * and Blair Brettmann
Georgia Tech
*  Correspondence: adobbs8@gatech.edu

**Abstract**: Improving the processing of propellant composites is pivotal for the production of novel material formulations that meet performance demands, integrate with new technologies, and are environmentally conscious. There is a need in the field to define fundamental formulation-processing relationships that can be leveraged to address the challenging nature of developing and processing highly loaded particulate composites. The key challenge of interest to this work is the formation of inhomogeneities in the particle spatial distribution upon shaping of the dense paste propellant precursor. Dense pastes undergo particle network deformation under applied stresses above the yield stress that often cause irreversible particle migration and the formation inhomogeneities. These inhomogeneities are then cured into the propellant grain and can hinder mechanical properties and performance. Herein, inhomogeneity formation after significant deformation is examined via rheological thixotropy testing and is investigated as a function of binder formulation. Model suspensions of inert glass microparticles were employed and the total solids content was kept consistent at 61.4 vol% of an efficient bimodal distribution of small and large glass spheres. Polymer concentration and molecular weight in the binder were systematically varied using polyvinylpyrrolidone (PVP) and polyethylene glycol (PEO) polymers dissolved in water. Thixotropy tests show that increasing polymer content effectively reduces inhomogeneity formation; and that viscosity is not the only predictor for thixotropic behavior in dense pastes. This observation may be due to polymer entanglements acting to stabilize the particle network. Elucidating the formulation-processing relationships between polymer formulation and composite thixotropy will provide insight into the complex rheology of dense pastes, and help enable informed polymer selection during formulation development.

### 3.14. Evaluation of DMA Data of HMX Based Explosive Formulation and of Its HTPB-IPDI Binder Alone

Manfred Bohn *, Peter Gerber and Michael Herrmann
Fraunhofer ICT
*  Correspondence: Manfred.Bohn@ict.fraunhofer.de

**Abstract**: The dynamic behavior determined by dynamic mechanical analysis (DMA) in torsion mode, of a high explosive charge (HE-charge) formulation called HX522 (HTPB-IPDI bonded with 85 mass-% HMX and 6.0 mass-% plasticizer DOA) is compared with its binder alone, called BV_HX522. The HE-charge was used in a Variable velocity HEmispherical Projectile Impact Test (VHEPIT). This is a modified version of the so-called “Steven Impact Test” (SIT) or “Steven Projectile Impact Test” (SPIT). The DMA was operated in the linear visco-elastic range with regard to applied maximum strains. The deformation frequency range was from 0.1 Hz to 30 Hz with HX522 and from 0.1 Hz to 10 Hz with BV_HX522. Data characterization and evaluation was performed by the shift of maximum temperatures of the loss factor with deformation frequency and with establishing of master curves via the Time-Temperature-Superposition (TTS) procedure. The parameterization of the shifts of the main maximum of the loss factor with deformation frequency, representing the glass-to-rubber transition (GRT), is made with standard and modified Arrhenius equation. For comparison, also the Williams-Landel-Ferry (WLF) parameters are calculated with the latter. The second characterization is in form of master curves. Their valid ranges are appraised also with the use of so-called “van Gurp-Palmen” (vGP) plots. For establishing the master curves, two optimization ways have been applied: optimization of the shift (1) using storage shear modulus G′ and (2) using loss shear modulus G′′.

### 3.15. Peculiarities in Assessing Chemical Inter-Substance Reactions of Energetic Materials

Manfred Bohn *
Fraunhofer ICT
*  Correspondence: Manfred.Bohn@ict.fraunhofer.de

**Abstract**: The often overseen effect of the temperature weighting already observable with reactions in parallel with different activation energies will be demonstrated and the consequences by this effect discussed. For reliable assessment the experimental determinations must be performed in such a way to cover the time-temperature load of the material at in-service conditions also by the test conditions. This is named TEL (thermal equivalent load) principle. Further only with certain extents, at least 4–6%, of reaction conversions at not to high temperatures a suitable assessment of chemical stability and chemical compatibility can be achieved. The interest with compatibility evaluation is to get the information on the inter-reaction conversion at defined test time and test temperature of substances in contact, means on the so-called inter-reactivity. The way to achieve this is not as simple as typical standards propagate. Also with compatibility, one encounters the peculiarity of temperature-weighted reactions. In adverse cases, one may assess compatibility at typical higher test temperatures but at low in-service temperatures the inter-reactivity is higher than expected.

The next question is the about the allowed extent of inter-reaction conversion with methods as gas generation, heat generation and mass loss. Until now, this question is not satisfactorily answered, especially with heat generation and gas generation, which are determined by heat flow microcalorimetry and vacuum stability test apparatus, respectively. A way to come to conclusive values will be outlined and discussed. The method mass loss is in advantage; therewith the extent of conversion can be determined unambiguously.

## 4. Session: Composites and Nanocomposites

### 4.1. Spinning a Greener Future: Biodegradable Silk Composite Fibers via Silkworm Diet Modification

Xiao Hu * and Melinda Deng
Rowan University
*  Correspondence: hu@rowan.edu

**Abstract:** This research introduces a groundbreaking method for producing biodegradable silk fiber materials by integrating silk with various biopolymers (e.g., cellulose) or biocompatible inorganic materials via an in vivo silkworm spinning process. This is achieved through a novel dietary approach for Bombyx mori silkworms, feeding them a range of biomaterial “foods”, such as cellulose acetate and microcrystalline cellulose. This strategy marks a significant advancement over traditional silk material production methods by eliminating the need for toxic chemical agents to dissolve silk fibers and simplifying the production process of silk-based fibrous materials directly through a biological system.

For the first time, our study successfully demonstrates the incorporation of biofillers into silkworm silk without negatively impacting the health of the silkworms or altering the size of their cocoons. Employing comprehensive analysis techniques such as Fourier transform infrared spectroscopy, scanning electron microscopy, mechanical tensile testing, and thermal analysis (DSC and TGA), we have identified the seamless integration of various biofillers into silk. Notably, an increase in beta-sheet content was observed, signifying a direct correlation with improved material strength. The results indicate that optimal enhancements in the properties of silk-based composite materials are achieved with lower concentrations of biocompatible fillers.

This innovative approach of combining silk with biofillers through dietary modifications opens new avenues for developing sustainable, strong textiles and materials for biomedical applications. Beyond offering an eco-friendly alternative to conventional plastics and materials, this technique broadens the scope of biodegradable materials across multiple sectors. Our findings underscore the transformative potential of silk-based fiber composites in material science, positioning them as a viable solution to the pressing global issue of plastic pollution.

### 4.2. The Impact of Ultrasound Sonication on Chitosan-Silk Fibroin Composite Films

Nagi Reddy Poluri and Xiao Hu *
Rowan University
*  Correspondence: hu@rowan.edu

**Abstract:** Ultrasound technology, a non-isothermal and non-toxic method, has gained considerable attention across diverse domains within chemical, biological, and food sciences. Operating within the frequency range of 20 kHz to 1 MHz, the effects of sonication have been extensively explored for their ability to modify material and functional properties, either through cavitation-induced alterations or by modifying the biopolymer structure and promoting both inter- and intra-molecular interaction forces. In this study, we investigate the effects of ultrasound sonication on the physical and molecular properties of chitosan-silk composite films with varying molecular weights of chitosan. Chitosan, derived from shrimp skeletons, and silk fibroin, obtained from Bombyx mori silk cocoons, were chosen as the constituents for the composite films due to their biocompatibility and film-forming properties. Composite films were prepared via a solution casting method, combining chitosan and silk solutions in different weight ratios and subjected to ultrasound sonication at 20 kHz for 10~30 min.

The effects of ultrasonication on treated and untreated samples were assessed using scanning electron microscopy (SEM), Fourier-transform infrared spectrometry (FTIR), differential scanning calorimetry (DSC), thermal gravimetric analysis (TGA), water contact angle measurements, mechanical testing, and enzyme degradation studies. It was observed that the material characteristics of the films were enhanced by ultrasound. FTIR results confirmed the presence of strong interactive hydrogen bonding between silk and chitosan in the composite films formed, and the β-sheet conformation of silk in the films was enhanced, resulting in a shift in the thermal peak. Compared with untreated samples, the mechanical properties of the films slightly increased, with improved tensile strength. Moreover, ultrasonication improved the enzyme degradation rate and flexibility of the films, and the water retention capacity of the films was slightly increased, making the films more favorable for cell proliferation and attachment. With enhanced flexibility, strength, and stability, ultrasound-treated chitosan/silk films could be superior materials for packaging or biomedical applications.

### 4.3. Multi-Scale Structure Regulation of Ceramizable Phenolic Aerogel Composites to Achieve Super Thermal Insulation and Extreme Thermal Protection

Yiming Yang *
Chinese Academy of Science, Institute of Chemistry
*  Correspondence: yiming924@iccas.ac.cn

**Abstract**: Thermal insulating materials play a vital role in personal protection, industrial thermal management, and space exploration. Due to the increasing need for thermal protection against high temperature oxidation extreme environment for aerospace mission, lightweight ablative phenolic aerogel composites with both thermal ablative and insulative properties had become emerging materials. Herein, we reported multiscale structure regulation of phenolic aerogel composites including molecular structure, hybridized elements distribution, microphase separation and nanoporous structure via synthetic and preparation design, and finally achieve comprehensive properties enhancement including mechanical properties, thermal insulation, ablative properties. A ceramizable Zirconium/Silicon hybridized phenolic resin was synthesized and its resin aerogels showed excellent nanoporous structure, good mechanical properties and low thermal conductivities. Furthermore, the hybridized elements showed atomic level of distribution within the aerogel network without phase separation. Based on this new type of resin, the prepared quartz fiber/aerogel nanocomposite perfectly inherits their nanoporous microstructure and fascinating properties, in particular, good thermal ablative and insulative properties. A 30 mm thick composite showed back temperature increasing of 38 °C in aerodynamic heating test by quartz lamp array at 1200 °C for 900 s, and significant reduced line ablation rate under 4 MW/m^2^ extreme heat flow from oxy-acetylene flame test. The ablated surface preserved nanoporous structure formed by ceramized zirconia, silica and carbon, and zirconium elements still dispersed uniformly with residual carbon. The evolution of hybridized elements during pyrolysis was tracked by FTIR, XPS and XRD, and analyzation of pyrolysis gas revealed that hybridized elements prevent the degradation of carbon ring at high temperature. This ceramizable lightweight composite presents huge application prospects in thermal protection and heat insulation field, especially in aerospace industry.

### 4.4. Enhancement of Rate of Crystallization of Thermoplastic Polymers by Carbon Nanoparticles

Joseph Menczel *
Thermal Measurements LLC
*  Correspondence: jmenczel@yahoo.com

**Abstract:** Nucleation during polymer crystallization is very important, because the developed crystal size can regulate the mechanical properties. Carbon nanoparticles are excellent nucleating agents, but their disadvantage is that the color of the final product can only be black.

It is unknown so far if the high nucleating efficiency of carbon nanoparticles is coming from the very large concentration of polar groups of the surface of the particles, or the extremely small size of these particles is more important. In order to clarify this question, a number of nucleated polymers was tested by DSC, and their crystallization properties were compared.

### 4.5. Adhesion-Diffusional-Based Corrosion Protection Mechanisms of Polyaniline-Primed Fluoropolymer Coatings

Mark Rigel Ali *, Szeemaine Tigno, Jackson Honeyman and Eugene Caldona
North Dakota State University
*  Correspondence: mark.ali@ndsu.edu

**Abstract:** In this study, we developed a series of dual-coating systems, composed of poly(vinylidene fluoride-co-hexafluoropropylene) (PVDF-HFP) and electrodeposited polyaniline (PANI) as the primary coating and primer, respectively, for stainless steel (SS). Prior to PVDF-HFP topcoat application, PANI (in either doped emeraldine salt (ES) or dedoped base (EB) form) was electrodeposited potentiodynamically on either etched or unetched SS substrate using H_2_C_2_O_4_ as the supporting electrolyte. Tape tests showed improved PVDF-HFP adhesion on EB-PANI primed substrates than on ES-primed samples. Impedance analysis and potentiodynamic chemistry were used to quantitatively investigate the electrochemical behavior and corrosion kinetics of the coated samples, respectively, in a 3.5 wt.% NaCl corrosive solution. Electrochemical results revealed that the PVDF-HFP/EB-PANI dual coating system exhibited superior corrosion resistance (low frequency impedance modulus, (~10^6^ Omega cm^2^) compared to either the dual PVDF-HFP/ES-PANI or pristine PVDF-HFP coating (~104 Omega cm^2^). Based on the correlation between electrochemical data and water uptake, high water diffusivity for ES-PANI-primed PVDF-HFP coating (diffusivity, D ~10.0 × 10^−10^ mm^2^ s^−1^) was obtained due to the ionic solvation of counter ions, while relatively lower diffusivity for EB-PANI-primed counterpart (D ~5.0 × 10^−10^ mm^2^ s^−1^) was observed due to the effective impingement of water ingress by the insulating hydrophobic EB-PANI undercoat. Overall, we investigated the impact of the electronic state of an electroconductive polymer primer on the corrosion resistance of a two-coat system with a fluoropolymer topcoat, and explored nuanced effects on adhesion and electrolyte diffusion, providing comprehensive insights and laying the foundation for tailored design strategies to enhance corrosion resistance, filling a critical gap in the current understanding and guiding future research in coating science innovation.

### 4.6. Advanced Poly(Naphthalene) Networks from BODA-Derived Resins: Building Blocks for High-Temperature Thermosetting Polymers, Composites, and High Yielding Carbon Precursor

**Ernesto Borrego** **^1^** ^,^*, Dennis Smith **^1^, Niroshani Abeynayake** **^1^** **and William Johnson** **^2^**
^1^  Hand Technologies, LLC
^2^  Mississippi State University
*  Correspondence: eborrego@handtechllc.com

**Abstract:** Carbon-carbon (C/C) composites are promising candidates for leading edges in hypersonic applications because of their inherently low density and excellent materials properties at extreme temperatures: high thermal conductivities, specific strength, and low coefficients of thermal expansion. Ultimate performance is dominated by microstructure, interphase stress transfer, and cohesion between the fiber and matrix. Bis-ortho-diynylarene (BODA)-derived resins (BDR) constitute a versatile platform of melt-processable resins capable of rapidly producing high performance matrix composites which include thermoset, carbon-carbon, and other aerospace and specialty carbon structure. Herein are described the fundamental properties of BDR, covering the following aspects: (i) melt-processing suitability for various micro-molding and composite fabrication techniques; (ii) facile reagent-free, catalyst-free, thermal-initiated polymerization into a polyarylene thermoset with outstanding thermal-oxidative stability, low heat release, flame resistance, and high carbon yield; (iii) the discrete molecular transformations occurring within this network during carbonization, considering variables such as temperature, heating rate, and copolymer structure; and (iv) the general properties of their resulting carbons and C/C composites.

### 4.7. Recyclable Thermoset Resin and Composites via Tailored Vitrimer Chemistry

Mary Danielson, Menisha Koralalage, Tomonori Saito and Anisur Rahman *
Oak Ridge National Laboratory
*  Correspondence: rahmana1@ornl.gov

**Abstract:** Plastics and composites have become an integral part of our daily lives, but their disposal has emerged as a significant global challenge. Establishing closed-loop circularity for plastics with an easy manufacturing process is crucial for a global circular economy. Adaptive polymeric materials that incorporate dynamic (reversible) bonds hold immense potential for creating sustainable future technologies. Dynamic bonds can selectively undergo reversible bond breaking and reformation in specific conditions that enable structural rearrangements in polymer networks, strongly improve the toughness, and provide recyclability, self-heal ability, and strong adhesion. In contrast, permanently crosslinked polymers (e.g., thermosets), comprising 15–20% of all produced polymers, do not have facile recyclability due to their unpliable nature. In our study, we upcycled a commodity thermoplastic elastomer, polystyrene-b-poly(ethylene-co-butylene)-b-polystyrene (SEBS) toward higher-value materials including tough adhesives and remarkably strong and closed-loop recyclable carbon-fiber reinforced vitrimers (CFRVs). These CFRVs exhibit excellent chemical and thermal stability, exceptional mechanical properties, interfacial adhesion, remarkable toughness as well as closed-loop circularity. We have also upcycled PET waste into vitrimer materials, which demonstrate exceptionally tough reversible adhesive properties on various substrates. Our strategy of incorporating dynamic covalent and noncovalent bonds into the polymer and the interface enables mechanically robust, tough adhesion, and fast processability/recyclability of thermoset materials. This presentation will summarize our effort in the design, synthesis, and processing of novel dynamically crosslinked polymers and their composites for the circular economy.

### 4.8. 3D-Printing of Partially Bio-Based Methacrylate Resin Composites with Surface Functionalized Carbon Fibers

**Karen Cortes-Guzman** **^1^** ^,^*, Zoriana Demchuk **^1^, Tomonori Saito** **^1^** **and Rigoberto Advincula** **^2^**
^1^  Oak Ridge National Laboratory
^2^  University of Tennessee—Knoxville
*  Correspondence: cortesguzmkp@ornl.gov

**Abstract:** Carbon fiber composite materials represent lightweight structural materials due to their high mechanical strengths, but there are several challenges to overcome especially in the interfacial interactions of the fibers and the resin matrix. If the wettability of the carbon fibers is not optimal, the fiber surface could introduce void areas between the fiber and the resin, where the composite could be more prone to failure and result in an overall reduction of the mechanical performance. Among the various ways to improve wettability of the carbon fibers, chemical bonding through surface functionalization provides a tunable method for high interfacial adhesion. Additionally, chemical bonding interactions between the fibers and the resin could provide better compatibility of the composite resin with 3D printing technologies. Usually, 3D printing of carbon fibers through light-mediated polymerization tends to be challenging. Their uneven distribution of the carbon fibers in the resin matrix, leading to precipitation of the fibers into the vat, and further blocking of the light source, reducing the penetration depth and therefore the polymerization. Here, we report a partially biobased methacrylate resin system, capable of supporting hydrazide functionalized carbon fibers, and allowing even distribution of the fibers in the resin, which results in uniform composite prints as opposed to the clearly visible gradient prints obtained with the pristine carbon fiber composite resins. The mechanical performance of the composites made with functionalized carbon fibers, remains consistent after various print cycles with a drop in ultimate tensile strength of only 0.1 MPa between first and 4th batch of printing, whereas the pristine carbon fiber composites exhibit a drop of ~3 MPa between first and 4th batch of printing. This composite design will enable obtaining strong polymer composites that benefit from the design freedom enabled by 3D printing technologies.

### 4.9. Enhanced Energy Storage with 3D-Printed Bimetallic Metal-Organic Frameworks and Reduced Graphene Oxide Hybrid Nanocomposite

Mahshid Mokhtarnejad *, Soheil Almasi, Mariana Milano-Benitez and Bamin Khomami
University of Tennessee
*  Correspondence: mmokhtar@vols.utk.edu

**Abstract:** Supercapacitors (SCs) with rapid recharge rate and substantial power output bridge the gap between conventional capacitors and batteries, making them an essential component in energy storage applications. Metal-organic frameworks (MOFs) are gaining prominence in SC technology due to their high porosity, tunable pore size distribution, and exceptional structural adaptability. When composited with other materials, such as carbon-based compounds, graphene oxide and graphene to produce hybrid nanocomposites (HNCs), they achieve the required high conductivity and chemical stability, making them ideal as high-performance electrode materials in SCs. This study introduces a fast and cost-effective method for synthesizing bimetallic MOFs composited with reduced graphene oxide (rGO). The experiments reveal that bi-MOF-rGO HNCs exhibit high specific capacitance and power density. Furthermore, we have successfully integrated these HNCs into an active layer of electrode material through sequential inkjet printing. The results of this experiment pave the way for the integration of these HNCs in energy storage applications, particularly in the development of high-performance printed electronics.

### 4.10. AI and Biomimetic Assembly for Soft Hybrid Materials and Energy Storage

Jihua Chen *
Oak Ridge National Laboratory
*  Correspondence: jihuac@gmail.com

**Abstract:** Tailored polymer template and organic molecules represent an important approach to guide the growth of functional nanostructures from solution [1,2]. The intermolecular interactions within those organic or ionic complexes can lead to thin films with fascinating structures, and at the same time, greatly improve their ability to build up unusual mechanical properties, transport charge, or store energy. However, the underlying nanostructures can be challenging and delicate to probe due to the weak interactions involved in the system. In this work, we demonstrated that using AI and low-dose analytical TEM, one can reveal these intriguing nanostructures and nanointerfaces effectively. An insulator polymer/small molecular organic semiconductor blend is used herein as an example for charge transport enhancement, while a polymer based salt complexes is showcased as an improved solid electrolyte. Results are also correlated with electrical measurements such as impedance testing, equivalent circuit modeling, or transistor output characteristics to further our understanding of the intriguing structure-induced functionality. Instance and semantic segmentation based on deep learning are shown to be instrumental for soft matter TEM analysis. Similar algorithms may be applied to other aspects of energy storage and soft matter systems for improved design and performance management [3].

**References**

1. Chen, J. *Nanomaterials* **2021**, 11, 2405.
2. Chen, J.; et al. *SmartMat 2*, 367–377.
3. This research was conducted at the Center for Nanophase Materials Sciences, which is a DOE Office of Science User Facility.

***This manuscript has been authored in part by UT-Battelle, LLC, under contract DE-AC05-00OR22725 with the US Department of Energy (DOE). The US government retains and the publisher, by accepting the article for publication, acknowledges that the US government retains a nonexclusive, paid-up, irrevocable, worldwide license to publish or reproduce the published form of this manuscript, or allow others to do so, for US government purposes. DOE will provide public access to these results of federally sponsored research in accordance with the DOE Public Access Plan (http://energy.gov/downloads/doe-public-access-plan).

### 4.11. Effect of Silica Particle Functionalization on the Bulk Viscoelastic Properties of Covalent Adaptable Network Composites

Jaehyun Cho *, Blair Brettmann and M.G. Finn
Georgia Institute of Technology
*  Correspondence: jcho361@gatech.edu

**Abstract:** Covalent Adaptable Networks (CANs) featuring dynamic covalent bonds have attracted considerable interest due to their reprocessability. Incorporating highly tunable silica particles into these networks not only enhances their mechanical properties but also facilitates diverse chemical reactions at the polymer-particle interfaces. To improve reprocessability, the crosslinked network structure can be controlled by adjusting the crosslinking density and the number of bond-exchangeable units, and the bond-exchange thermodynamics. In this study, we aim to comprehend the bulk bond-exchange behavior as stress relaxation in composite systems. Deconvoluting the factors influencing the overall structure-property relation in bulk vitrimer composites is crucial. By introducing surface-functionalized silica particles capable of dynamic crosslinking, we have developed robust and reprocessable polymer composites. This research into the particle-polymer interfaces and bulk crosslinked structures will enhance the practical application of these advanced materials.

## 5. Session: Sustainable Polymers

### 5.1. Novel Reprocessable Cross-Linked Polymer Networks: Importance of Rheology and Thermophysical Property Characterization in Advancing Sustainability, Recycling, and Upcycling

John Torkelson *
Northwestern University
*  Correspondence: j-torkelson@northwestern.edu

**Abstract:** We have developed several classes of covalent adaptable networks (CANs) that have the properties and performance of thermosets at use conditions but, unlike thermosets, allow for melt-state reprocessing at elevated temperature (T) with complete recovery of cross-link density and associated properties after reprocessing. We will give examples from three classes of CANs demonstrating how cross-links formed with dynamic covalent bonds provide for recycling and robust of such networks: (A) thermoplastic polyethylene and ethylene-containing copolymers upcycled into CANs by reactive radical-based processing, (B) isocyanate-based polyurethane and polythiourethanes networks, and (C) non-isocyanate polythiourethane. The robustness of network properties even after multiple reprocessing steps is demonstrated via thermophysical property and rheological characterization, including elevated-T creep and stress relaxation tests and T-dependent dynamic mechanical analysis (DMA). DMA provides a simple method for defining effective cross-link density in CANs via the value of the storage modulus in the quasi-rubbery plateau region, which is essentially equivalent to the rubbery plateau modulus; Flory’s ideal rubber elasticity theory states that effective cross-link density is proportional to the rubbery plateau modulus. The T-dependence of the storage modulus also provides characterization of the nature of the dynamic covalent bond. Dissociative dynamic bonds exhibit a decrease in rubbery plateau modulus at conditions where the dynamic character is active; in contrast, associative dynamic bonds yield a rubbery plateau modulus that increases linearly with absolute T because the cross-link density is essentially constant. An ideal thermoset will exhibit nearly zero elevated-T creep and stress relaxation up to the degradation T. In contrast, a CAN exhibits nearly zero elevated-T creep up to T values when the dynamic nature of the dynamic covalent bonds is incipient and then exhibits creep dominated by the bond and chain dynamics. As such, elevated-T creep characterization may define the T limits of CAN utility. Similarly, a CAN exhibits stress relaxation with a T dependence that may be defined by an activation energy that is equal to the bond dissociation energy for dissociative dynamic bonds or defined by chain or segmental dynamics for associative dynamic bonds. The relative rapidity of stress relaxation at elevated T can be useful in indicating the T conditions under which CANs may be (re)processed by melt extrusion or injection molding, processes that cannot be employed with permanently cross-linked thermosets. We will provide examples of extrudable/injectable CANs.

### 5.2. Upcycling PET Waste into Vitrimers with Exceptionally Tough Adhesion

Mary Danielson *, Tomonori Saito and Anisur Rahman
Oak Ridge National Laboratory
*  Correspondence: danielsonmk@ornl.gov

**Abstract:** Plastic pollution is a significant and growing problem worldwide. Single-use plastics and packaging make up most commercial products, leading to waste volumes that far exceed the capacity for recycling. To integrate this excess plastic waste into a circular economy, advanced chemical recycling technologies are essential. In this work, we demonstrate the upcycling of the commodity plastic poly(ethylene terephthalate) (PET) into circularly recyclable vitrimer products. PET waste is deconstructed via aminolysis into macromonomers, which are then crosslinked with various acetoacetate-functionalized molecules to form vitrimers. These vitrimers exhibit superior mechanical performance compared to the original PET. The PET vitrimer can be efficiently depolymerized again through aminolysis, allowing for the recovery of both the monomer and crosslinker, thus validating its closed-loop recyclability and providing a sustainable solution for plastic waste management. Importantly, our PET vitrimer materials exhibit exceptional adhesive performance on various substrates, with lap shear strengths exceeding 12 MPa. The final adhesive formulation of the PET vitrimers is both solvent-free and curable at room temperature, which is important for practical deployment. Our research demonstrates the potential for upcycling existing plastic waste into new high performance materials, paving the way towards a circular economy.

### 5.3. Crystallization Kinetics of Blends of Sustainable Aliphatic Polyesters Derived from 1,18 Octadecanedioic Acid and Diols with Dissimilar Length

Briona Carswell and Rufina G Alamo *
FAMU-FSU College of Engineering
*  Correspondence: alamo@eng.famu.fsu.edu

**Abstract:** Bio-sourced polyethylene-like polymers have been synthesized as possible substitutes for commodity polymers derived from fossil fuel feedstocks. Their crystalline and mechanical properties are of interest in the path to developing a more sustainable, circular plastics economy. We are interested in understanding their solidification behavior from the melt and the semicrystalline morphology acquired under different crystallization modes. In this work, we have studied the crystallization kinetics of two sets of blends of polyesters: PE-2,18/PE-10,18 and PE-4,18/PE-10,18 in the whole range of composition. Under fast crystallization, PE-2,18 develops hexagonal crystals, while PE-10,18 develops orthorhombic crystals under any crystallization mode. Hexagonal PE-2,18 exhibits superior tensile deformation behavior than the orthorhombic counterpart. Hence, the first type of blend is chosen to probe if the hexagonal phase with superior mechanical properties also develops in mixtures of polyesters. An additional interest is probing if the blends co-crystallize and if the minimum in the isothermal crystallization rate observed in PE-2,18 vanishes due to the loss of layered crystallites. Because PE-4,18 and PE-10,18 have equivalent crystal packing and crystallization rates, blends of these polyesters will be essential to demonstrate their co-crystallization behavior or lack thereof by following their crystallization kinetics contrasted with data from the pure homopolymers. The results from both types of blends will be discussed in the context of reusing or recycling these polyethylene-like materials.

### 5.4. Upcycling of Waste Plastic into New Materials via Multiple Olefin Metathesis

**Jeff Foster** **^1^** ^,^*, Joshua Damron **^1^, Chao Guan** **^1^, Ilja Popovs** **^1^, Anisur Rahman** **^1^, Nick Galan** **^1^, Tomonori Saito** **^1^, Isaiah Dishner** **^1^** **and Jackie Zheng** **^2^**
^1^  Oak Ridge National Laboratory
^2^  University of Tennessee—Knoxville
*  Correspondence: fosterjc@ornl.gov

**Abstract:** The accumulation of plastic waste in the environment has emerged as a significant global challenge. In 2018 alone, 27 MMT (million metric tons) of used consumer plastic was landfilled in the US, with most items—water bottles, food packaging, grocery bags—discarded after just a single use. Beyond the detrimental environmental effects incurred by this “take-make-waste” mentality, the energy and feedstock resource costs must also be considered. Significant energy and petroleum-based material are consumed in the process of plastics manufacturing and these resources are essentially lost when the plastic item is discarded. Thus, there is a critical need to develop energy- and atom-efficient upcycling strategies that convert waste plastics into new, value-added materials to offset the energy losses associated with landfilling or chemical deconstruction.

In this contribution, we report a polymer upcycling strategy that uses cyclic, monofunctional monomers to insert new functionality into polyalkenamers via a tandem ring-opening metathesis polymerization (ROMP) and cross-metathesis. Our approach is differentiated from previous works on olefin metathesis depolymerization or backbone scrambling in that new polymeric materials are generated in a single, highly efficient synthetic transformation. Using polycyclooctadiene (PCOD) as a model platform, we show that backbone insertions occur rapidly and efficiently under mild reaction conditions, affording copolymers with various modified backbone structures. Through rational monomer design, we obtain semicrystalline copolymers, amorphous copolymers with blocky sequences, and copolymers with highly alternating segments. We demonstrate that our process can be applied to upcycle commercial polybutadiene in the presence of other waste plastics and impurities. Importantly, many of the bonds formed during the synthesis of the original sample are conserved in these examples. Thus, our strategy fosters significant progress towards mitigating the loss of feedstock resources and energy associated with landfilling waste polymers while affording value-added functionality.

### 5.5. Selective Deconstruction and Upcycling of Step-Growth Polymers

Tomonori Saito ^1^^,^*, Nick Galan ^1^, Anisur Rahman ^1^, Ke Cao ^1^, Zoriana Demchuk ^1^, Jeff Foster ^1^, Bobby Sumpter ^1^, Jackie Zheng ^2^ and Md. Arifuzzaman ^3^
^1^  Oak Ridge National Laboratory
^2^  University of Tennessee—Knoxville
^3^  Re-Du
*  Correspondence: saitot@ornl.gov

**Abstract:** Engineering plastics based on step-growth polymerization such as poly(ethylene terephthalate) (PET), polyamides (PA), polyurethanes (PU), and polycarbonate (PC) comprise ~30% of the global plastic production. The catalytic deconstruction is one of the major paths for chemical recycling of step-growth polymers. Although there has been progress on their chemical recycling especially PET, most step-growth polymers are not recycled because of the difficulty in depolymerization to pure building blocks especially from mixed waste in an energy efficient manner. Here, we have developed a tailored organocatalyst to enable low energy depolymerization pathways for step-growth polymers. Our catalyst allows glycolysis of PET, PA, PU, PC and their multiple mixture at moderate temperature with high yield. A wide range of post-consumer plastics waste, such as bottles, packagings, foams, carpets etc. is readily deconstructed into valuable building blocks with exceptional efficiency. The Life Cycle Assessment indicates that the reproduction of various engineering plastics from the deconstructed monomers will result in a significant reduction in greenhouse gas emissions (82–95% reduction) and energy input (68–94% reduction). Furthermore, we have developed a path to deconstruct those step-growth polymers to selective length of oligomers. We have utilized those deconstructed building blocks to synthesize upcycled polymers, and the upcycled polymers can be further deconstructed to reusable building blocks. Such circular design contributes to establishing new closed-loop circularity of polymers by energy efficient selective deconstruction and upcycling of various engineering plastics. This presentation will update our progress on chemical deconstruction of step-growth polymers and their upcycling.

### 5.6. Catalytic Mechanopolymerization for the Synthesis of Sustainable Polymers

**Toby Nelson** **^1^** ^,^*, Mohammad Ebqa’ai **^2^**
^1^  University of Tennessee
^2^  Oklahoma State University
*  Correspondence: tnelso31@utk.edu

**Abstract:** Catalytic mechanopolymerization is defined here as the use of mechanochemical methods to polymerize monomers in the presence of a catalyst. This extension on traditional polymer mechanochemistry takes advantage of the carbon-carbon cross-coupling reaction technology for the synthesis of advanced materials. The talk will explore the mechanosynthesis of poly(lactic acid)s (PLA)s, PLA nanoparticles and PLA-drug conjugates. I will discuss lessons learned about the effect of ball-milled reaction parameters such as milling ball size, reaction time, collision frequency and temperature influence on conversion, degree of polymerization, molecular weights, polydispersity and yield.

### 5.7. 3D Printing Fully Recyclable, Self-Healing Vitrimer from 100% Sustainable Sources

Sargun Singh Rohewal *, Logan Kearney and Amit Naskar
Oak Ridge National Laboratory
Correspondence: srohewa1@vols.utk.edu

**Abstract:** The widespread adoption of 3D printing across various sectors is driven by its advantages in fabrication convenience, efficiency, cost-effectiveness, and waste reduction. Traditionally, thermoplastic inks have dominated the 3D printing landscape due to their recyclability and ease of processing. However, the inability to recycle thermoset-based 3D prints poses significant environmental challenges and economic inefficiencies, particularly in large-scale engineering applications. To mitigate this issue, recent advancements in vitrimer technology offer a promising avenue for developing fully recyclable 3D printing inks for thermoplastic polymers. Vitrimer is an innovative class of polymeric materials which demonstrates traditional thermoset-like mechanical and chemical resilience while still being able to flow on demand akin to thermoplastic through relaxation caused by rapid bond exchange in the networked structures.

In this study, we developed an ester-based vitrimer ink by utilizing functionally enriched lignin oligomer and epoxidized natural rubber, yielding a dual-phase structure within the vitrimer system. The incorporation of lignin-based hard phase plays a pivotal role in enhancing stiffness, resulting in outstanding mechanical performance. Concurrently, the rubber-based soft phase facilitated fast relaxation through increased productive exchange reactions, fostering supramolecular interactions such as hydrogen bonding, p-p stacking, and ionic interactions. These interactions collectively expedited spontaneous diffusion and contributed to potential self-healing properties. The resulting vitrimeric ink exhibited heat-driven malleability, allowing for the printing of complex 3D geometries that can subsequently be recycled into new inks for successive printing cycles. Post-annealing of printed samples near their topology-freezing temperature (~150 °C) markedly improved interlayer adhesion through fast associative dynamic covalent bond exchange reactions. Our results demonstrated that the printed materials can be fully recycled for multiple cycles while maintaining thermal and mechanical properties, thus offering a sustainable solution for 3D printing applications.

### 5.8. Biopolymer and Polymer Synthesis with Biocatalysts

Yue Yuan *
Oak Ridge National Laboratory
*  Correspondence: yuany@ornl.gov

**Abstract:** Development and utilization of renewable feedstocks is crucial when addressing sustainability in advanced material development. Although biobased polymers have been considered sustainable alternatives of petroleum-based plastics, a lack of deep understanding of the functional group-driven molecular interactions and their behaviors in processing has limited their application in large scale manufacturing. In parallel, developing a more sustainable synthesis route for polymers is crucial in the overall picture of sustainable materials manufacturing, including using enzymes instead of harsh condition or metallic catalysts. Enzymes, nature’s biocatalysts, were discovered and still can only be synthesized in biological systems. Using enzymes in non-biological environment enables new opportunities in low energy solutions for emerging areas such as carbon capture, water treatment and green chemistry.

We have developed functionalization strategies in both organic phase and aqueous phase to control the functionality of polysaccharide-based nanomaterials, and the modification of functional groups in polysaccharides resulted in tailored molecular interactions, thus improving the material performance in the application. Therefore, we also conduct fundamental studies of molecular interactions of polysaccharide-based materials using methods such as scattering and Isothermal Titration Calorimetry (ITC), to study the structure of molecular complexes and thermodynamics of the molecular complex, respectively. Here at Oak Ridge National Laboratory, we are developing deuterated polysaccharides as model systems for structure and dynamic studies using neutron techniques.

We would also present ongoing work on enzymatic ring-opening reaction in organic solvents, with focus on workflow development for enzyme performance and selectivity, including medium to high throughput assays. Since 90’s, exploratory work of polyester enzymatic synthesis has been conducted with hydrolases for polycondensation, transesterification, and ring-opening mechanisms. In the past decades, the aim of enzymatic polyester synthesis often targets at molecular weight, particularly in aliphatic polyester synthesis. In this work, we used serine superfamily hydrolases to develop a workflow to compare the enzyme performance in regards of selectivity. In the method development we minimized the purification steps while retaining the repeatability of the data, and the properties of the biosynthesized oligomer and polymer products were analyzed for their structure and thermal properties. The workflow bridges the biocatalyst characteristics (e.g., sequences, secondary structure) with performance in non-biological environment.

### 5.9. Advancing Sustainability and the Circular Economy through Advanced Molecular-Level Recycling of Automotive Plastics

**Mahshid Mokhtarnejad** **^1^** ^,^*, Gary Hawkins **^2^, Hendrik Mainka** **^3^**
^1^  University of Tennessee
^2^  Eastman Chemical Co.
^3^  Volkswagen Group of America
*  Correspondence: mmokhtar@vols.utk.edu

**Abstract:** The pressing global plastic waste crisis necessitates a transformative shift towards sustainable materials utilization. In this regard, Volkswagen Group of America Inc. (VWGoA) and Eastman Chemical Company are taking the lead in designing and driving circular economy solutions for automotive plastics by employing innovative molecular recycling technologies. This approach facilitates the conversion of traditionally hard-to-recycle plastic waste streams into their molecular components for the production of virgin-grade raw materials. These feedstocks are then used to produce specialty plastics and fibers with quality and performance equivalent to their virgin-grade counterparts. Our work targets a wide range of plastic waste streams including mixed plastics, colored plastic materials, post-consumer and industrial car components, and textiles, with the ultimate goal of confronting recycling challenges while providing environmental sustainability. We have shown that by using this molecular-level chemical recycling process, we can not only offer a high-quality alternative to virgin fossil-based plastics, but also reduce greenhouse gas emissions by 25–50% compared to conventional petroleum-derived automotive plastics. Our team has demonstrated the automotive viability of these sustainable materials by creating multiple demonstration parts on production equipment: an Atlas Cross Sport Spoiler Assembly and Passat R-Line Diffuser. These parts have successfully met all essential mechanical and thermal criteria, including scratch and chemical resistance, adhesion, tensile and flexural modulus as well as Vicat softening and glass temperature tests. This exemplifies the efficacy of the molecular-level chemical recycling process in recycling mixed plastics for automotive application. These components serve as examples of our commitment to sustainability and circular economy principles, paving the way for a cleaner and more resource-efficient automotive industry.

## 6. Session: Rheology

### 6.1. Elongational Viscosity of Large Cyclic Macromolecules

**Gregory McKenna** **^1^** ^,^*, Dongjie Chen **^1^, Julia Kornfield** **^2^, Hojin Kim** **^2^, Zhiyuan Qian** **^3^, Judit Puskas** **^4^, Gabor Kaszas** **^4^, Kristof Molnar** **^5^**
^1^  North Carolina State University)
^2^  California Institute of Technology
^3^  Texas Tech University
^4^   Ohio State University
^5^   Semmelweis University
*  Correspondence: gbmckenn@ncsu.edu

**Abstract:** Macrocyclic polymers represent a sort of last frontier in the polymer physics community’s efforts to understand dynamics of chain-like macromolecules. In this work results from an investigation of the extensional rheology of entangled cyclic poly(3,6-dioxa-1,8-octanedithiol) (PolyDODT) with an entanglement number Zw based on the linear counterpart of 58. In addition, two cyclic polyisobutylene disulfides (PIB-disulfide) with Zw = 10.7 and Zw = 5.5 were investigated. From prior work on ring rheology [1] we think that the PIB disulfide rings are not fully entangled, though their linear counterparts would be. The materials were synthesized by a novel synthesis route: radical ring-opening redox polymerization (R3P) [2]. In contrast to literature reports for a cyclic polystyrene with Zw ≈ 10, i.e., unentangled, ring, neither the well entangled cyclic PolyDODT and the unentangled cyclic PIB-disulfide do not show a pronounced strain hardening behavior when compared with. At the same time, the Weissenberg number dependence of the steady-state extensional viscosity is similar for the R3P synthesized rings and the polystyrene rings. The possible explanations for the discrepancies and similarities of the extensional rheology of the R3P synthesized cyclic polymers (PolyDODT and PIB-disulfide) and the cyclic polystyrene are discussed.

**Acknowledgments:** The authors greatly appreciate the U.S. Department of Energy, Office of Science, Basic Energy Sciences for its support under Awards DESC0018657 (DC and GBM), DESC0018891 (K.M., J.E.P. and G.K.) and DE-SC0018655 (H.K. and J.A.K). The authors at OSU also gratefully acknowledge funding from USDA-NIFA under Hatch project number OHO01417. GBM and DC thank the John R. Bradford Endowment at Texas Tech University for partial support and GBM is thankful to the Department of Chemical and Biomolecular Engineering at NC State University for partial support.

**References**

1. Chen, D.; Molnar, K.; Kim, H.; Helfer, C.A.; Kaszas, G.; Puskas, J.E.; Kornfield, J.A.; McKenna, G.B. Linear Viscoelastic Properties of Putative Cyclic Polymers Synthesized by Reversible Radical Recombination Polymerization (R3P). *Macromolecules* **2023**, *56*, 1013–1032.
2. Puskas, J.E.; Sen, D. Synthesis of biodegradable polyisobutylene disulfides by living reversible recombination radical polymerization (R3P): macrocycles? *Macromolecules* **2017**, *50*, 2615–2624.

### 6.2. Non-Linear Mechanics of Foams under Tensile, Compressive, and Shear Deformations: Importance for Design of Helmet Padding

**Shuang Jin** **^1^** ^,^*, Gregory McKenna **^1^, Linh Pham** **^1^, Felipe Santos** **^2^, Hossein Bahreinizad** **^2^, Sudeesh Subramanian** **^2^** **and Suman Chowdhury** **^2^**
^1^  North Carolina State University
^2^  Texas Tech University
*  Correspondence: sjin8@ncsu.edu

**Abstract:** The purpose of protective helmets is to prevent or reduce head injuries, including traumatic brain injury. The properties required of the helmets are different depending on the specific requirements. For example, while a football player mainly needs head protection from a slow-moving but heavy body, military or riot control personnel will need ballistic protection as well. Current combat helmets, while having sufficient ballistic protection against high-velocity projectiles such as bullets, have limited linear and angular impact protections against low-velocity hand-thrown projectiles such as a brick, which are commonly seen by riot police in modern civil unrest situations. Therefore, a novel helmet design that provides both concussive and ballistic protection from head traumas is needed. Traditionally, polymer foams such as polyurethane have been widely used in helmet padding systems due to their good compressive response. Yet, that alone is not sufficient to meet all the requirements because the isolation of the brain during an angular impact requires shear resistance and, possibly, tensile resistance. Classical isotropic finite mechanics models are not applicable in this case and characterization of the tensile and shear responses in addition to the compressive response becomes important. In fact, the behavior of foams is noted for having a tension-compression asymmetry. They can also exhibit linear or non-linear behavior under relatively large shear deformations. Such complex foam behavior has been rarely investigated in helmet pad design. Especially, the viscoelastic response, i.e., frequency and rate dependence, has seen limited investigation. Here we report on a systematic study of 6 foam materials under tensile, compressive, and shear deformation. The work considers linear and non-linear response as well as rate, frequency, and temperature dependence. We are also developing a constitutive model to describe the mechanical behavior of foams to give some insights in making helmet pads for novel helmet design that provides both concussive and ballistic protection.

### 6.3. Spatial Heterogeneity in Block Co-Polymers and Its Impact on Dynamics

Bret Tantorno *, Lori Hoover and Gregory McKenna
North Carolina State University
*  Correspondence: bwtantor@ncsu.edu

**Abstract:** It remains an important challenge to develop the relationships between spatial and dynamic heterogeneities in polymeric materials. The use of co-polymers provides a means of manipulating the size and distribution of spatial heterogeneities and this offers the opportunity to gain insights into the correlation between spatial and dynamic heterogeneities. In this study, four polystyrene (PS)/polyisoprene (PI) co-polymers were investigated: A random PS-PI chain (Sample 1), a 5k PS end block on a random PS-PI chain (Sample 2), a 10k PS end block on a random PS-PI chain (Sample 3), and a 10k PS block in the middle of a random PS-PI chain (Sample 4). Total molecular weights were approximately 100 kg/mol. Oscillatory experiments were conducted across temperatures ranging from 20 °C to 90 °C at frequencies from 0.1 rad/s to 100 rad/s. Time-temperature-superposition (TTS) analysis yielded mastercurves, indicating subtle differences among the sample’s dynamics. To provide more clarity, van Gurp-Palmen (vGP) plots, sensitive to subtle beak-downs of frequency-temperature superposition, were then constructed. Surprisingly, the plots revealed that Sample 1 had the least favorable behavior, i.e., exhibited deviations from superposability in the vGP plots. At the same time Sample 4 had the most favorable behavior, contradicting initial expectations. Our initial hypothesis expected Sample 1 to be the most favorable and Sample 4 to be the least favorable, based on the assumption that Sample 1, the random PS-PI chain, would be more spatially homogeneous than Sample 2, 3, and 4, with the PS blocks. Further investigation is planned to characterize the spatial heterogeneities in the co-polymer samples. In addition, we plan to perform large-amplitude oscillatory shear (LAOS) and mechanical spectral hole burning (MSHB) experiments on the samples as these methods have been successful in past work in the fingerprinting of heterogeneous dynamics [1,2]. Future research will focus on understanding how the types of spatial heterogeneity typical of co-polymers of different micro- or nano-structure influence linear dynamics as well as the response in nonlinear spectroscopies such as LAOS and MSHB.

The authors are grateful for the support of the ACS-PRF grant 60750-ND7 and the National Science Foundation grants 2105065 and 2219327, each for partial support. In addition, we thank the College of Engineering and the Department of Chemical and Biomolecular Engineering for the support of the project. We also thank L. Taylor, R. Register and R. Priestley at Princeton University for generously sharing the samples.

**References**

1. Chandra Hari Mangalara, S.; Paudel, S.; McKenna, G.B. Mechanical Spectral Hole Burning in Glassy Polymers—Investigation of Polycarbonate, a Material with Weak β-Relaxation. *J. Chem. Phys*. **2021**, *154*, 124904.
2. Mangalara SC, H.; McKenna, G.B. Mechanical Hole-Burning Spectroscopy of PMMA Deep in the Glassy State. *J. Chem. Phys*. **2020**, *152*, 074508.

### 6.4. Rheometric Advances to Overcome Challenges Associated with Characterizing Pressure Sensitive Adhesives (PSA’s)

**Abhishek Shetty** **^1^** ^,^*, Gunther Arnold **^2^, Jose Alberto Rodriguez Agudo** **^2^** **and Andre Braun** **^2^**
^1^  Anton Paar USA
^2^  Anton Paar Germany
*  Correspondence: abhi.shetty@anton-paar.com

**Abstract:** Pressure sensitive adhesive (PSA) based products are widely used in a plethora of industries ranging from packaging, automotive, medicine, architecture to home care. Mechanical and thermal analysis methods are essential to guarantee good quality and performance of the final products. Modern rheometers offer the user a wide range of both rotational and oscillation-based tests. Oscillatory measurements such as dynamic mechanical analysis (DMA) allow characterizing the viscoelastic properties of the PSA within a broad range of frequencies and temperatures. DMA is a preferred method when it comes to quality control and formulation of pressure sensitive adhesives (PSA) since it provides a lot of valuable information in one single test. Time-temperature superposition (TTS) measurements which are a subset of DMA are of great interest to characterize samples over a wide range of temperatures and frequencies. One often runs into rheometric limitations while performing such tests on a single sample. For instance, due to the huge range of temperature and frequency which are probed one has to change the configuration which includes changing the plate diameter and using a new sample. When performing torsional tests with PSA, one challenge is that both, the sample between the plates as well as the components of the rheometer (such as measuring system and motor) are exposed to torsional stresses. If the sample is rather liquid and much less stiff than the measuring system, all torsional energy is absorbed by the sample, i.e., the deflection angle of the motor is the same as the deflection angle of the sample. However, if the sample is very stiff, e.g., adhesives exposed to very low temperatures, the rheometer components are also torsionally twisted resulting in a deflection angle of the sample being less than the deflection angle at the motor. Another challenge is oscillating at high frequencies. Due to the inertia of the moving rheometer components and the sample, the torque necessary to accelerate the measuring system becomes eventually much larger than the sample torque. This can lead to spurious results and one has to account for such effects. In this presentation, we will show the strategy to perform TTS measurements on PSAs over a huge temperature (200 °C) and moduli (6–7 decades) range without having to change the configuration and/or the sample. Some advanced rheo-combinatorial techniques like Rheo-Raman and Rheo-Dielectric Spectroscopy for adhesive characterization will also be presented.

### 6.5. Designing Polymer-Ceramic Suspensions for Extrusion-Based Additive Manufacturing: Effects of Non-Adsorbing Polymer Addition on Shear Rheology

Ria Corder ^1^^,^*, Arezoo Ardekani ^2^, Jeffrey Youngblood ^2^ and Kendra Erk ^2^
^1^  University of Tennessee—Knoxville
^2^  Purdue University
*  Correspondence: rcorder@utk.edu

**Abstract:** Concentrated ceramic particle suspensions can be extruded to form 3D structures for biomedical, aviation, and electronic applications using the extrusion-based additive manufacturing technique of direct ink writing (DIW). Designing suspensions or “inks” for DIW requires a balance to be achieved between material strength and viscosity. Non-adsorbing polymers are often added to DIW inks as viscosity and yield stress modifiers. Understanding the mechanisms by which free polymers contribute to the concentrated suspension rheology, and notably how they affect the onset of shear thickening (an undesirable phenomenon for DIW), is crucial to the design of 3D printable polymer-ceramic suspensions.

In this work, we examine the rheology of aqueous colloidal alumina suspensions containing non-adsorbing polyvinylpyrrolidone (PVP) of varying molecular weights and at concentrations within the dilute and semi-dilute non-entangled regimes. PVP addition causes large increases in both viscosity and dynamic yield stress. Discontinuous shear thickening occurs above a critical onset stress (τ min) which is dependent on both PVP loading and molecular weight. We relate the overall suspension rheology to the polymer conformation in the suspending medium, demonstrating how non-adsorbing polymer addition can extend the processing window during extrusion by increasing τ min. The fundamental insights gleaned herein regarding how non-adsorbing polymers contribute to suspension rheology can help to inform the design of 3D-printable polymer-ceramic suspensions and better understand their potential processing limitations.

### 6.6. Rheological Considerations for Material Design and Selection of Print Parameters for Thermoset Additive Manufacturing

Madeline Wimmer * and Brett Compton
University of Tennessee—Knoxville
*  Correspondence: mwimmer1@vols.utk.edu

**Abstract:** Thermoset material extrusion additive manufacturing promises design freedom and flexibility of materials that are wear resistant in a wide range of environments. During printing, the viscoelastic ink experiences a wide range of shear states; after moderate pressure is applied to the feedstock, the material reaches a high shear state while extruding through the nozzle before it structurally recovers to a low shear state after being deposited. The ink must be able to support the weight of additional layers of material without yielding or buckling until cured. Methods to improve the stability of liquid resin address the stiffness of the uncured feedstock, the rate and degree of cure after deposition, or a combination of the two. When designing the ink, this can look like including a rheological modifier or blending with a solid resin. Improving the stability via the crosslinking reaction may involve a long, low-temperature cure schedule when using a heat-activated curing agent or utilizing a chemically or UV-initiated system to begin crosslinking soon after deposition. This talk will discuss the competing softening and stiffening behavior thermoset inks experience as the temperature of their environment increases.

Latent, heat-activated curing agents are popular as they can reach higher thermal and mechanical properties than other crosslink initiation methods. Additionally, these inks boast room temperature processing times on the order of days to months allowing for a feedstock to be compounded in advance and printed from a single reservoir. However, because the ink must be able to extrude during deposition when moderate pressures are applied, the storage modulus and yield stress of the ink are limited by hardware constraints. These constraints then govern the force, and subsequent layers of material, the uncured ink can support before a wall buckles or yields. Because temperature and viscosity are inversely related for uncured ink, the heated cure profile must be designed so that the print does not collapse during the early stages of cure. To build taller parts, the ink must start crosslinking after extrusion but before the print is finished. With a chemically initiated reactive system, the resin and curing agent are mixed during deposition, resulting in a feedstock that is highly shear thinned. As the ink structurally recovers, the exothermic crosslinking reaction increases the temperature of the system which can either decrease or increase the viscosity of the feedstock depending on the degree of cure. These competing behaviors lead to a wide range of shear states across the part depending on both the local temperature and degree of cure. This talk will conclude with a discussion on how rheology can be utilized to prescribe temperature limits when curing a thermoset print.

### 6.7. Characterizing Gelation of Thermally Cured Thermoset Composites for Additive Manufacturing and Semiconductor Packaging

**Stian Romberg** **^1^** ^,^*, Paul Roberts **^1^, Polette Centella** **^1^, Christopher Soles** **^1^, Chad Snyder** **^1^, Jonathan Seppala** **^1^, Anthony Kotula** **^1^** **and Sarah Lehrman** **^2^**
^1^  National Institute of Standards and Technology
^2^  University of Maryland–Baltimore County
*  Correspondence: stian.romberg@nist.gov

**Abstract:** Additive manufacturing and semiconductor packaging frequently utilize thermoset composites, despite key processing challenges. Additively manufactured parts are highly susceptible to structural collapse before the gel point, whereas in packaging applications the gel point marks the moment when cure shrinkage begins to manifest as residual stress. Therefore, understanding how rheology evolves with the extent of reaction (i.e., conversion) is critical to designing successful curing protocols for these composites. Here, we demonstrate that the use of two emerging measurement techniques can provide important information about the curing process. The rheo-Raman instrument provides simultaneous measurements of rheology and conversion. This data enables the development of models that more accurately and fully describe the rheology-conversion relationship than currently available methods. Optimally Windowed Chirp (OWCh) measurements enable quick, low-strain measurements to identify the gel point of rapidly curing composites with small linear viscoelastic regimes, which has proven challenging for more traditional measurements. When combined with the rheo-Raman results, OWCh measurements help us to determine the gel conversion, which can guide material selection and processing parameters.

### 6.8. Rheological Analysis of Recycled Feedstock Materials for Large Format Additive Manufacturing

**Roo Walker** **^1^** ^,^*, Ally Collier **^1^, Chad Duty** **^1^, Matthew Korey** **^2^, Amber M. Hubbard** **^2^** **and Soydan Ozcan** **^2^**
^1^  University of Tennessee—Knoxville
^2^  Oak Ridge National Laboratory
*  Correspondence: rwalke60@vols.utk.edu

**Abstract:** As the demand for more sustainable manufacturing practices and materials grows, it is important not only to identify potential material streams and applications, but also to understand how processing conditions are affected by the degradation imparted during the recycling and repelletization process. Exploring the utilization of recycled short fiber materials in emerging manufacturing techniques like large format additive manufacturing (LFAM) aids in developing a more robust and sustainable supply chain [1]. This study assesses the viability of using carbon fiber acrylonitrile butadiene styrene (CF-ABS) machining scrap, a previously under-utilized material waste stream, as a recycled feedstock material for LFAM applications [2].

Prior to printing, rheological characterization via frequency sweeps were completed for the recycled CF-ABS material, pristine CF-ABS, and neat ABS. This information was then interpreted using a viscoelastic printability model [3]. The four primary pass/fail conditions to determine the printability of the recycled feedstock material were pressure-driven flow, bead formation, bead functionality, and component functionality. This investigation showed that the recycled material is printable even though it exhibits a significantly reduced (97%) complex viscosity compared to the pristine counterpart, which was directly correlated to significant fiber length attrition imparted by the surface machining process. Combining the rheological analysis with a viscoelastic printability model helped to understand observed changes in processing behavior and contributed to a deeper understanding of how the rheological behavior of the material may affect changes in printed microstructure (e.g., fiber orientation) and affect bulk mechanical properties like ultimate tensile strength.

The recycled material was successfully printed, characterized, and compared to a pristine counterpart. It was found that the tensile strength and elastic modulus of the recycled material decreased in the print direction compared to the pristine CF-ABS. However, in the layer-wise direction, it was found that the recycled material exhibited no significant change in elastic modulus and a significant increase in tensile strength. This work not only indicates that machining scraps could be a viable material stream for recycled LFAM feedstock materials but also demonstrates the utility of rheological analysis when assessing and qualifying new recycled feedstock materials for LFAM applications.

**References**

1. Armstrong, K.O.; Kamath, D.; Zhao, X.; Rencheck, M.L.; Tekinalp, H.; Korey, M.; Ozcan, S. Life cycle cost, energy, and carbon emissions of molds for precast concrete: Exploring the impacts of material choices and additive manufacturing. *Resour. Conserv. Recycl.* **2023**, *197*, 107117. https://doi.org/10.1016/j.resconrec.2023.107117.
2. Walker, R.; Korey, M.; Hubbard, A.M.; Clarkson, C.M.; Corum, T.; Smith, T.; Duty, C. Recycling of CF-ABS machining waste for large format additive manufacturing. *Compos. Part B Eng.* **2024**, *275*, 111291. https://doi.org/10.1016/j.compositesb.2024.111291.
3. Duty, C.; Ajinjeru, C.; Kishore, V.; Compton, B.; Hmeidat, N.; Chen, X.; Kunc, V. What makes a material printable? A viscoelastic model for extrusion-based 3D printing of polymers. *J. Manuf. Process.* **2018**, *35*, 526–537. https://doi.org/10.1016/j.jmapro.2018.08.008.

### 6.9. Role of Glass Fiber on the Flow-Induced Crystallization of Poly(Ether Ether Ketone)

Xiaoshi Zhang ^1^^,^*, Alicyn Rhoades ^1^, Jason Alexander ^2^, Jiho Seo ^2^, Benson Jacob ^2^, Ralph Colby ^2^, Gijs Kort ^3^ and Richard Schaake ^3^
^1^  Penn State—Behrend
^2^  Penn State University
^3^  SKF
*  Correspondence: xvz5402@psu.edu

**Abstract:** Poly(ether ether ketone) (PEEK) is widely recognized for its thermal stability and mechanical strength among engineering thermoplastics. However, there is a research gap in understanding PEEK’s crystallization properties, especially in its composites. This study investigated the flow-induced crystallization of glass fiber reinforced PEEK in contrast to neat PEEK resin. Using rheology-based methods, specific work was applied to the resins at distinct isothermal crystallization temperatures. In the quiescent and low work regimes, glass fibers hinder PEEK crystallization. Conversely, under higher work conditions, glass fibers facilitate flow-induced crystallization more rapidly. To understand the role of glass fiber in both situations, synchrotron X-ray scattering techniques were employed to provide insights into the crystal unit cell and lamellar structure orientation of PEEK resin. Additionally, X-ray CT was used to extract information about glass fibers under different specific work conditions. Glass fibers, due to their length and rigidity, align more easily, subsequently creating a shear field for PEEK to align and reduce resin relaxation.

### 6.10. Measuring Liquid Crystallinity by Thermomechanical Analysis of Shape Memory Epoxy

Collin Pekol *
University of Tennessee
*  Correspondence: cpekol@vols.utk.edu

**Abstract:** A method for testing shape memory capability in liquid crystalline epoxy networks (LCEN) is discussed. This technique combines thermomechanical tension testing with calorimetry and X-ray scattering techniques to elucidate the unique relationships between morphology and behavior in LCEN. This work aims to merge the separate thermal analytical methods to measure shape memory polymer functionality, quantify the degree and orientation of liquid crystal domain formation, and measure the degree of shape memory capability. This method provides the means to tune the shape memory behavior via morphological control of LCEN. Cyclic temperature modulation of the LCEN under constant loading revealed high-strain shape memory effects controlled by the isotropic transformation of liquid crystalline domains across a first-order thermal transition. Liquid crystalline domain alignment is induced by thermomechanical processes and confirmed via wide-angle X-ray scattering measurement. Through discussion and examination of observed shape memory behavior and liquid crystalline morphology, a direct correlation of volume fraction of liquid crystalline domains and magnitude of recoverable strain characteristic of shape memory.

## 7. Session: Modeling & Simulation

### 7.1. Introducing Molecular Topology in Polymer Informatics

Konstantinos Vogiatzis *
University of Tennessee
*  Correspondence: kvogiatz@utk.edu

**Abstract:** The prediction of a variety of polymer properties from their monomer composition has been a challenge for polymer informatics. Artificial intelligence/machine learning (AI/ML) has greatly advanced this field by providing valuable tools for a more effective exploration of the material space, synthesis space, and material characterization. The application of AI/ML for chemical and polymer applications requires the conversion of molecular structures to a machine-readable format known as a molecular representation. The choice of such representations impacts the performance and outcomes of chemical machine learning methods. We have developed a new concise molecular representation derived from persistent homology, an applied branch of mathematics that capture the topological features of the molecular units. We have demonstrated its applicability in a high-throughput computational screening of a molecular database for the discovery of CO2–philic groups that can be used as building blocks of polymeric membranes for N2/CO2 separations. More recently, we released POLYMERGNN, a multitask machine learning architecture that relies on polymeric features and graph neural networks and provides accurate estimates for polymer properties based on a database of complex and heterogeneous data. This talk is part of the AI/ML in Materials and Characterization topic.

### 7.2. Remote Monitoring of Vitamin Degradation in Food and Pharmaceutical Products

**Bertrand Roduit** **^1^** ^,^*, Damiano Rickenbach **^1^, Patrick Folly** **^2^,** **Lexandre Sarbach** **^2^** **and Richard** **Baltensperger** **^3^**
^1^  AKTS SA
^2^  armasuisse
^3^  University of Applied Sciences of Western Switzerland
*  Correspondence: b.roduit@akts.com

**Abstract:** Everyone is regularly confronted with the question of food shelf life. Shelf life is indicated on the label in the form of a “best before” date, which indicates how long the food will retain its specific properties, such as taste, aroma, appearance and other qualities. The processes that cause food to deteriorate during storage or transport are complex and involve chemical, biochemical, microbial and physical reactions.

Kinetic analysis is a practical way of predicting food spoilage. This method makes it possible to assess shelf life by measuring certain quality parameters over time.

Despite the complexity of reactions, changes in food quality are often assumed to follow simple reaction rates, such as zero-order or first-order kinetics. Unlike traditional methods, our approach takes into account all possible kinetic models and their combinations, including single and multi-step reaction models.

In addition, food spoilage analysis is generally not carried out continuously, which limits data collection. This limitation requires the use of appropriate statistical methods to analyse the sparse data in the kinetic studies. Our kinetic workflow uses Akaike and Bayes statistical criteria to select the best kinetic model to describe changes in product properties as a function of time and temperature [1,2].

Another common assumption is that food is stored or transported at a stable, constant temperature. However, temperature is a crucial factor affecting food quality, which is why it is necessary to constantly monitor temperature variations.

This study presents a new system which enables continuous remote monitoring of the remaining shelf life of perishable products, even in the event of unexpected temperature changes in the cold chain. The temperature data collected by the dataloggers is used in the best kinetic models or their combinations to continuously calculate the remaining shelf life at different temperatures. As an example, the evolution of the concentration of vitamins in food as a function of time and temperature will be presented. This solution enables us to estimate the thermal behaviour of any perishable product, whose changes in properties can be analysed using kinetic analysis and temperature-time data from dataloggers [3–5].

**References**

1. AKTS-Thermokinetics Software (Thermal Aging version TKsd 6.02), AKTS SA, Technopôle 1, Sierre, Switzerland. Available online: https://www.akts.com (accessed on 17 May 2024).
2. Roduit, B.; Hartmann, M.; Folly, P.; Sarbach, A.; Baltensperger, R. *Thermochim. Acta*. **2014**, *579*, 31.
3. Clenet, D. *Eur. J. Pharm. Biopharm*. **2018**, *125*, 76.
4. Neyra, C.; Clenet, D.; Bright, M.; Kensinger, R.; Hauser, S. *Int. J. Pharm*. **2021**, *609*, 121143.
5. Evers, A.; Clenet, D.; Pfeiffer-Marek, S. *Pharmaceutics* **2022**, *14*, 375.

### 7.3. Thermal Hazards Assessment of Three Azo Nitrile Compounds Using AKTS

Dylan Aljovic *, Cuixian Yang, Thomas Vickery, Megan Roth and Daniel Muzzio
*  Correspondence: dylan.aljovic@merck.com

**Abstract:** Commercially available azo radical initiator compounds provide efficient initiation of many chemical reactions and have been used extensively in synthetic applications across multiple industries. However, many azo compounds are thermally unstable at ambient or even sub-ambient temperatures. Azo compound decomposition, initiated by heat and/or light, forms carbon radicals and significant amounts of nitrogen gas resulting in rapid pressure increases, presenting a safety challenge for shipping, storage, and usage. This presentation outlines a study [1] in which the exothermic activity and gas generation of three azo nitrile compounds was examined using various thermal calorimetry techniques with only 5 mg to 1 g samples. The testing results were subsequently analyzed using differential iso-conversional methods [2] to obtain key decomposition kinetics parameters and generate a model. The model was then validated with additional experimentation and was utilized to predict several key safety parameters for scale-up applications, including the Self-Accelerating Decomposition Temperature (SADT) and Time to Maximum Rate (TMR). Additionally, we evaluated the effect of phase transitions on the kinetic behavior of thermal decomposition and the experimental determination of autocatalytic decomposition. This methodology for assessing thermal decomposition kinetics enhances our safety understanding of storage, handling, and scale-up of the selected azo compounds.

**References**

1. Yang, C.; Aljović, D.; Vickery, T.P.; Roth, M.; Muzzio, D.J. Thermal hazards assessment of three azo nitrile compounds. *Process Saf. Environ*. **2023**, *178*, 1026–1044.
2. Roduit, B.; Hartmann, M.; Folly, P.; Sarbach, A.; Brodard, P.; Baltensperger, R. Thermal decomposition of AIBN, Part B: Simulation of SADT value based on DSC results and large scale tests according to conventional and new kinetic merging approach. *Thermochim. Acta* **2015**, *621*, 6–24.

### 7.4. Understanding the Performance of Holographic Photopolymers Using Thiol-Ene Chemistry via Numerical Simulations

Yunfeng Hu *, Christopher Bowman
University of Colorado—Boulder
*  Correspondence: yunfeng.hu@colorado.edu

**Abstract:** To fully explore the highest available refractive index modulation (∆n) in holographic photopolymers, thiol-ene photopolymerization emerged as a promising writing chemistry. Recent research has demonstrated its capability of achieving a remarkably high ∆n of 0.04. However, the achieved ∆n was only 50% of the theoretical maximum (∆n), suggesting a significant loss, possibly attributable to an imbalance between reaction and diffusion. Few works haven been done to incorporate the step-growth kinetics of thiol-ene polymerization into the modeling of holographic recording, compared to the well-established models using chain-growth kinetics of acrylates. Thus, we are developing a comprehensive model that integrates thiol-ene kinetics to understand the unique mechanism involved thiol-ene holographic recording. This effort aims to demonstrate the distinct materials considerations in thiol-ene formulation compared to conventional acrylates, and gain insights that can potentially guide the development of future thiol-ene formulations.

### 7.5. Exploring Machine Learning Models to Predict SAM Number and Compressive Strength of Optimized Aggregate Graded Structural Concrete Mixes

Sazzadul Saykat *, Debalina Ghosh, Ammar Elhassan, Saeed Davar and John Ma
University of Tennessee—Knoxville
*  Correspondence: ssaykat@vols.utk.edu

**Abstract:** Accurate prediction of material performance can reduce the number of trial tests and increase the efficient use of raw materials. On the other hand, optimized aggregate gradation in concrete can reduce the cement content, shrinkage, material cost, and carbon footprint and can improve strength and durability. Therefore, there is a need for a systematic approach to evaluate the optimized aggregate graded concrete mixes and predict the mechanical properties of the concrete. This research aims to combine a predictive algorithm with experimental methods for combined aggregate gradation in concrete to help engineers optimize concrete mixtures while saving time and money. The current study investigates machine learning (ML) methods, such as Artificial Neural Networks, decision trees, K-nearest neighbor, and Support Vector Machines, for predicting the compressive strength and Super Air Meter SAM number value of different combined aggregate graded concrete because of their autonomy in completing difficult tasks. To this aim, about 100 concrete mixtures were subjected to laboratory testing to gather data, and the findings were utilized to train and test the models. Additionally, SHAP (Shapley Additive Explanations) values are used in this study to determine which input variable most impacts the compressive strength and SAM number characteristics of optimal mixtures.

## 8. Session: 3D Printing/Additive Manufacturing

### 8.1. 3D Printing for Smart/Advanced Applications

Jose Bonilla-Cruz *
Research Center for Advanced Materials (CIMAV)
*  Correspondence: jose.bonilla@cimav.edu.mx

**Abstract:** Nowadays, additive manufacturing (AM, also known as three-dimensional (3D) printing) is considered a novel emerging field with practical applications in many fields of knowledge. This contribution will summarize applications using 3D printing of polymers, ceramics, and composites. Super-hydrophobic and para-hydrophobic 3D printed surfaces, novel ceramic photocatalytic scaffolds, IA applied to estimate viscoelastic properties of ceramic pastes 3D printed by DIW, new aqueous formulations of resins by DLP, and thermomechanical properties of nanocomposites based functionalized graphene at low-loadings with extraordinary mechanical behaviors.

### 8.2. 3D-Printed Polymeric Membranes for Water Purification and Carbon Capture Applications

**Eugene Caldona** **^1^** ^,^*, Dianne Gutierrez **^2^, Lucas Kilpatrick** **^3^** **and Rigoberto Advincula** **^3^**
^1^  North Dakota State University
^2^  University of the Philippines—Diliman
^3^  University of Tennessee—Knoxville
*  Correspondence: eugene.caldona@ndsu.edu

**Abstract:** Membranes find applications in water purification and carbon capture, however, traditional manufacturing techniques and materials limit the size and geometry of such membrane constructions. This work focuses on the design and fabrication of different additively manufactured porous polymeric membranes for high-efficiency oil/water, colloidal, and carbon dioxide separation applications. Firstly, membranes, composed of fluoropolymer matrix with varying amounts of silica particles, were 3D printed via direct ink writing. The printed membranes displayed water repellency and superoleophilicity, allowing oil to pass, but rejecting water, through its openings. Such surface characteristics are mainly attributed to the synergistic effect of surface roughness brought by the presence of silica particles and the fluoropolymer’s low surface energy. Secondly, elastomeric membranes containing silica were developed and 3D printed for CO_2_/N_2_ gas separation. These membranes exhibit high thermal stability and stretchability over 100% of their initial, and high permeability for CO_2_ gas, but with slightly low selectivity for CO_2_/N_2_ gas pair. Overall, the performance and properties of these membranes demonstrate the potential of 3D printing as an economical and sustainable fabrication method in developing advanced materials for a wide variety of practical applications.

### 8.3. Designing for Thermoforming Process via Thermomechanical Characterization

Segun Talabi *, Tyler Smith, Brittany Rodriguez, Ryan Ogle, Julian Charron, Sana Elyas, David Nuttall, Vlastimil Kunc, Vipin Kumar and Ahmed Hassen
Oak Ridge National Laboratory
*  Correspondence: talabisi@ornl.gov

**Abstract:** This paper presents a material characterization study aimed at facilitating the design process for thermoforming of additively reinforced polyethylene terephthalate (PETG) polymer sheet. The characterization methods employed include thermogravimetric analysis (TGA), differential scanning calorimetry (DSC) analysis, heat deflection temperature analysis (HDT) and microstructural examination. The TGA provides insights into the thermal stability and decomposition behavior of the base and deposition materials, while DSC analysis elucidates their thermal transitions such as glass transition temperature and melting points necessary for choosing the thermoforming temperature. Additionally, HDT analysis offers valuable information regarding the viscoelastic properties the materials under varying constant load and increasing temperature. The microstructural examination provides information about the extent of interfacial adhesion between the substrate and reinforcing layer.

### 8.4. 3D Printing of Low to Medium Solid Loaded Epoxy Micro-Composite Inks: Characterizing and Improving Printability for Tunable Material Properties

Zane Smith *
University of Tennessee
*  Correspondence: zsmith31@vols.utk.edu

**Abstract:** 3D printing of polymer composites has gained considerable traction in the last decade. In recent years, one technique of high interest is an extrusion-based AM technique called direct ink writing (DIW), that offers high flexibility in material selection with the ease of processability for composite inks. DIW utilizes a viscous and thixotropic ink that, once printed, is chemically cured to obtain the final AM part. Throughout literature, a common material for DIW is a commercial-grade bisphenol A-based epoxy resin formulated into nano-composite inks. Various studies have optimized the enhancement of material properties by integrating micro- and nanofillers such as chopped carbon fiber (CF), carbon nanotubes (CNTs), graphene, and the like. Although these studies have improved the polymer composites mechanical and sometimes electrical properties, there are significant limitations in the obtained properties. Increasing the loading of fillers within the ink can increase the mechanical integrity but also drastically increase the difficulty of processing and printability. In this work, epoxy resin composites with both micro- and nanofillers are studied to overcome the limitations of processability and printability by utilizing the interfacial interaction the CNTs can introduce when added with milled/chopped CF. Through thermomechanical, spectroscopic, and mechanical characterization, the DIW-printed epoxy composites will be studied to determine if chopped CF’s mechanical, thermal, and electrical properties with the addition of CNTs can be enhanced with lower solid loading percentages than previously reported. Additionally, a comprehensive rheological study on the percolation network effects with the addition of micro- and nanofillers is discussed.

### 8.5. Considerations for Large-Format Additive Manufacturing of Bio-Based Feedstocks in Natural Environments: Polymer Degradation, Aging, and Weathering

Caitlyn Clarkson *, Amber M. Hubbard, Katie Copenhaver, Halil Tekinalp and Soydan Ozcan
Oak Ridge National Laboratory
*  Correspondence: clarksoncm@ornl.gov

**Abstract:** Bio-based materials offer a considerable advantage in designing lower carbon footprint feedstocks compared to many synthetic feedstocks, especially in large-format additive manufacturing (LFAM) where the incumbent feedstocks are based on carbon-fiber and glass fiber-filled composites. The inherently lower embodied energy and carbon footprint of natural materials, and often lower cost, is passed on to the composite feedstock and is desirable for industry applications such as those in buildings and construction. This presentation will highlight findings in understanding weatherability and aging of biobased feedstocks being developed for 3D printing, specifically polylactic-acid (PLA)- wood flour (WF) composites used to create BioHome3D [1]. Weathering and aging of the materials throughout the application’s lifetime can cause color and gloss changes in the material appearance, as well as changes in the thermal and mechanical performance (e.g., embrittlement, changes in tensile strength, thermal conductivity). Additionally, considerations for the next generation of biobased LFAM feedstocks will be addressed, with key findings on the development of biodegradable LFAM feedstocks, which may provide an avenue for easier disposal in AM-construction applications when post-use contamination limits recyclability.

**Reference**

1. Advanced Structure Composites Center. BioHome3D. University of Maine. 2024. Available online: https://composites.umaine.edu/biohome3d/ (accessed on 28 May 2024).

### 8.6. Comprehensive and Multidimensional First-Principles Kinetic Modeling of Free-Radical Photopolymerization for Additive Manufacturing

Christopher Bowman *
University of Colorado—Boulder
*  Correspondence: Christopher.Bowman@colorado.edu

**Abstract:** Photopolymerization processes are ubiquitous in industrial and academic applications, including additive manufacturing, coatings and adhesives, holography, lithography, biomedical devices, and optics. Despite the widespread utility of photopolymerization and its resulting products, the accurate and robust prediction of end-use material properties remains difficult due to the complexities inherent in photopolymerizing systems.

In this talk, decades of advances in understanding and simulating photopolymerization kinetics are synthesized into a single comprehensive and multidimensional first-principles model. The model accommodates key features of photopolymerizing materials, including diffusion limitations, oxygen inhibition, light attenuation, mass and heat transfer, reaction-diffusion, composition-dependent material properties, and chain-length dependent kinetic parameters. From only two straightforward kinetics experiments, robust kinetic parameter values for a polymerizing monomer are determined and can be applied to a wide variety of photopolymerizations with differing geometries, boundary conditions, light intensities, photoinitiator concentrations, and resin formulations.

The model readily predicts gradients in conversion, material properties, temperature, and reaction rates that emerge from the complex interaction of reaction and diffusion phenomena. In one dimension, the model is shown to predict gradients that emerge in depth, including interlayer effects that occur during layer-by-layer additive manufacturing. In three dimensions, edge effects and interaction with the surroundings are included, enabling prediction of three-dimensional voxel fidelity given a variety of polymerization conditions and formulations.

Although validated for model systems of free-radical chain-growth photopolymerizations in bulk and thin films, the kinetic model is sufficiently modular to accommodate any photopolymerization reaction with known mechanism. This includes step-growth polymerization, mixed-mode reactions, chain-transfer, undesired side reactions, and alternative polymerization techniques such as free-radical frontal polymerization. Here, the model will be applied to the specific complexities associated with additive manufacturing, presenting how the photopolymerization systems can be optimized for such processes.

### 8.7. Additive Manufacturing of Polymers with Closed-Loop Circularity

Tomonori Saito ^1^^,^*, Karen Cortes-Guzman ^1^, Mary Danielson ^1^, Anisur Rahman ^1^, Zoriana Demchuk ^1^, Jeff Foster ^1^ and Sungjin Kim ^2^
^1^  Oak Ridge National Laboratory
^2^  University of New Mexico
*  Correspondence: saitot@ornl.gov

**Abstract:** Over 400 million tons of solid plastics are globally produced annually and only ~9% of those are currently recycled in U.S. Establishing closed-loop circularity of plastics with a facile manufacturing path is critical for global circular economy. When commodity plastics are upcycled into higher-performance materials with facile processability, a sustainable closed-loop manufacturing would become reality. Additive manufacturing (AM) of such upcycled plastics to custom-designed structures accomplishes energy and resource efficient low-carbon closed-loop manufacturing. We hereby open a circular upcycling of a commodity plastic into a higher-performance vitrimer with fused deposition modeling or direct ink writing, resulting in robust printout properties comparable to crosslinked thermosets. Vitrimer exhibits mechanical robustness and chemical resistance because of its covalent network formation, but it can also be malleable by reconfiguring reversible crosslinks through the associative bond exchange at elevated temperature, making it recyclable. Due to its crosslinked nature, the upcycled vitrimers provide stronger, tougher, solvent-resistant 3D objects and separatable from unsorted plastic waste. Tailoring the vitrimer composition overcomes the major challenge of (re)printing crosslinked materials, allowing multi-cycle printing. This presentation updates our efforts on AM of upcycled commodity plastics, especially upcycled vitrimers.

### 8.8. AI/ML in Polymers and Additive Manufacturing

Rigoberto Advincula *
University of Tennessee—Knoxville/Oak Ridge National Laboratory
*  Correspondence: rca41@case.edu

**Abstract:** The coming prevalence of artificial intelligence and machine learning (AI/ML) in polymer materials will rapidly optimize synthesis and manufacturing. Using Bayesian and statistical methods enables the applying logic-derived design and regression analysis into an otherwise trial-and-error approach in polymer synthesis, fabrication, and characterization. In this talk, we demonstrate continuous flow reaction chemistry methods to enable unit operation optimization and the possibility of autonomous design and synthesis using a hierarchical approach and learning. The automation for online monitoring is possible with improved instrumentation and the development of a feedback loop learning for possible deep learning (DL) development. Additive manufacturing (AM) is essential in fabricating parts and objects with high complexity and high performance. The use of nanocomposites enables highly improved properties. With AI/ML, it is possible to optimize both manufacturing and characterization methods. This talk will show how finite element analysis (FEA) and modified characterization methods improve workflow.

## 9. Session: Thermophysical Properties

### 9.1. Revisiting the Al-Fe-Si Liquidus Surface for Designing the Next-Generation of Impurity-Tolerant Aluminium Alloys

**Paul Lafaye** **^1^** ^,^*, Jean-Philippe Harvey **^1^** **and Vincent Rioux-Frenette** **^2^**
^1^  Centre for Research in Computational Thermochemistry
^2^  Polytechnique Montreal
*  Correspondence: lafaye.paul88@gmail.com

**Abstract:** The aluminum industry faces significant challenges in reducing greenhouse gas emissions by shifting from primary aluminum production to a circular economy. This transition necessitates the use of scrap and waste materials, which introduce high concentrations of impurities, mainly Fe and Si, into new alloys. Due to their relative nobleness, these impurities are difficult to remove with conventional recycling methods without dilution using primary metals. Consequently, Fe and Si content tends to increase during recycling, complicating the control of brittle Al-Fe-Si intermetallic phase precipitation. Addressing this issue requires the development of impurity-tolerant alloys.

To contribute to this challenge, the present project aims to revisit the liquidus projection of the Al-Fe-Si system across all Al-rich phase fields and accurately measure their isothermal lines. Approximately 50 alloys were synthesized, annealed, and characterized using differential scanning calorimetry (DSC) to determine liquidus temperatures and reassess ternary invariant reaction temperatures. The measured liquidus temperatures were combined with existing literature data and compared with predictions from Calphad modelling of the Al-Fe-Si system, available in the literature. Our results indicate that discrepancies between modeling and measurements are phase-field dependent. This could be partially explained by the design strategy of thermodynamic models for solid solutions (also called sublattice models), which often prioritize practical considerations over crystal chemistry.

By providing a detailed reassessment of the liquidus projection in the Al-Fe-Si system, this research contributes to the fundamental understanding necessary for designing next-generation, impurity-tolerant aluminum alloys. This is crucial for enhancing the recyclability of aluminum and supporting the industry’s transition towards sustainable practices.

### 9.2. Investigating the Influence of Hydrated Aluminum Powder on Metal Oxidation Reaction Correlated with Heating Rate

Mahmuda Ishrat Malek * and Michelle Pantoya
Texas Tech University
*  Correspondence: ishrat.malek@ttu.edu

**Abstract:** Aluminum (Al) particles contain high potential chemical energy (31 MJ/Kg) and are widely used in many energy generation applications. The Al_2_O_3_ passivation layer formed in the presence of air surrounding the Al core influences the combustion mechanism of Al particles by acting as a barrier to reactions. Hydrating the Al surface can replace the passive Al_2_O_3_ layer with Al(OH)_3_ and change the oxidation reaction mechanism thus energy release behavior. Hydrated Al, perceived to offer enhanced reactivity owing to the absence of the inhibitory Al_2_O_3_ layer, exhibits the opposite results in Differential Scanning Calorimetry (DSC) and Thermogravimetric Analysis (TGA) experiments. The hydrated Al exhibits 44.7–63.14% reduced endothermic heat flow due to diminished melting in different DSC environments compared to its Al_2_O_3_-coated counterpart. The amount of oxidized shell from 520 °C to 745 °C was 1.59–2.36% less in hydrated Al particles in different environments. This phenomenon is attributed to the unique characteristics of hydrated aluminum, particularly its propensity for pooling, which retards the melting process and impedes alumina shell growth. Furthermore, the heat flow of hydrated Al increased by 12.1% from 5 KPM to 20 KPM heating rate implying that the faster the heating rate is, the less impeding there will be for the oxidation mechanism in hydrated Al. This observation underscores the intricate relationship between shell formation, particle morphology, and combustion reactivity and provides insights into the necessary conditions for the combustion of hydrated aluminum particles to effectively utilize the altered properties of their shell in energy generation applications.

### 9.3. Thermophysical Properties of Lunar Regolith Simulant LHS-1 and Ice Sublimation Kinetics in Its Mixtures with Frozen Water

Evgeny Shafirovich * and Dominic Austen
University of Texas—El Paso
*  Correspondence: eshafirovich2@utep.edu

**Abstract:** A recently developed lunar regolith simulant LHS-1 is used currently to advance the technologies for water extraction from icy regolith in the polar regions of the Moon. However, thermophysical properties of LHS-1 at low temperatures have not been studied yet, which hinders the development of these technologies. It is also unclear whether experimental data on the rate of ice sublimation in LHS-1/ice mixtures can be applied to the actual icy regolith of the Moon. In the present paper [1], the specific heats and thermal diffusivities of LHS-1 and its finer version LHS-1D were studied at low temperatures using differential scanning calorimetry and laser flash analysis. The thermal diffusivities of LHS-1 and LHS-1D have been measured in the temperature range of 148–300 K in a helium environment at pressures of about 90 kPa and less than 1 Pa. The reduced pressure decreased the thermal diffusivity by several times owing to the decreased role of helium in the heat transfer during the test. The thermal diffusivity also decreased with increasing temperature and decreasing particle size. The specific heats of dry simulants have been measured in the temperature range of 110–320 K. The obtained values are close to the specific heats of the Apollo regolith samples at the same temperatures. The specific heats of LHS-1 and LHS-1D, mixed with water, have been measured in the temperature range of 110–340 K. The theoretically expected increase in specific heat due to the addition of 3–5 wt.% ice was within the experimental error. The ice sublimation rates in LHS-1/ice and LHS-1D/ice mixtures have been measured at temperatures of 240, 247, and 258 K at pressure of 4 Pa. The sublimation rate decreases with time for all samples, which may be associated with increasing vapor pressure over the subliming surface as the sublimation front propagates downward. The analysis of the obtained data has shown that the area of the subliming surface is much less than the surface area of the simulant particles. This should be taken into account when the results of experiments with simulant/ice mixtures are applied to the actual icy regolith on the Moon.

**Reference**

1. Austen, D.H.; Shafirovich, E. Thermophysical properties of lunar regolith simulant LHS-1 and ice sublimation kinetics in its mixtures with frozen water. *Thermochim. Acta* **2024**, *731*, 179648.

### 9.4. Thermal Properties of Diglucoses

Asya Bakhtina *
Mondelēz International MDLZ
*  Correspondence: asya.bakhtina@mdlz.com

**Abstract:** Glass transition temperatures of 11 disaccharides of glucose (diglucoses) were measured by DSC and compared to data in the literature. Most of the materials appear to easily absorb water on storage and crystallize either as an anhydrous or a hydrate form. Crystallized samples were converted to amorphous state in situ in the DSC pan and glass transition temperature was determined. During the measurement temperature of decomposition of the samples had to be taken into consideration, due to the fact that it is close to the melting points. Temperatures of decomposition for the samples were estimated from the analysis of evolved gases analysis by TGA-MS. Glass transition temperatures of the diglucose samples were found to vary slightly from the data in the literature, most likely due to different measurement conditions, sample storage and thermal histories

### 9.5. A Tale of Two Step-Growth Cyclopolymerizations. Synthetic Approaches to Semi-Fluorinated Polyaryl Ethers and Carbon Precursor Resins

**Dennis Smith** **^1^** ^,^*, Gustavo Munoz **^1^, William Johnson** **^1^, Kari Chamberlain** **^1^, Charles Pittman** **^1^** **and Ernesto Borrego** **^2^**
^1^  Mississippi State University
^2^  Hand Technologies, LLC
*  Correspondence: dsmith@chemistry.msstate.edu

**Abstract:** The race toward hypersonic flight dominance depends upon environmental durability and manufacturability of “hot structures” produced from carbon fiber matrix composites wherein the matrix materials are chosen from carbon and/or ceramic elements formed by CVD or pyrolysis of suitable precursors. Although commercial methods based on carbonization of phenolic resins have produced carbon-carbon composites (C/C) for 50+ years, production rates demanded now are severely limited due to low carbon yield, multiple re-infusion steps, and slow carbonization requirements. To address this critical need, our collaborative laboratory is developing resin technology based on the highly efficient thermal (Bergman) cyclopolymerization and copolymerization of bis-o-diynylarene (BODA) monomers affording intermediate reactive resins which can be melt processed, thermally cured to polynaphthalene networks, and pyrolyzed (1000 °C) to high yield (83%) carbon-carbon composites [1]. In addition, specialty semi-fluorinated aromatic ether polymers from step-growth polymerization of (1) fluoroalkenes, and (2) aryl ethers and hexafluoroacetone hydrate (via EAS) is presented. Well established perfluorocyclobutyl (PFCB) aromatic ether polymers and related architectures have demonstrated application as low k insulators, PEM fuel cells, hosts for emissive or electro-optic chromophores, optically tunable films (low loss, variable RI) with high thermal stability and thermally crosslinkable without the use of post-curatives [2]. An overview of both material platforms and commercialization efforts are presented.

**References**

1. Borrego, E.; Athukorale, S.; Gorla, S.; Duckworth, A.; Johnson, W.; Kundu, S.; Toghiani, H.; Farajidizaji, B.; Pittman, C.; Smith, D.W., Jr. High Carbon Yielding and Melt Processable Bis-ortho-Diynylarene (BODA)-Derived Resins for Rapid Processing of Dense Carbon/Carbon Composites. *Compos. Part B* **2022**, *242*, 110080, and references therein. BODA Resin samples are available from Hand Technologies, LLC (handtechllc.com).
2. Munoz, G.; Chamberlain, K.; Athukorale, S.; Ma, G.; Gu, X.; Pittman, C.U., Jr.; Smith, D.W., Jr. Teaching Old Polymers New Tricks. Improved Synthesis and Anomalous Crystallinity for a Lost Semi-Fluorinated Polyaryl Ether via Interfacial Polymerization of Hexafluoroacetone Hydrate and Diphenyl Ether. *Macromol. Rapid Commun*. **2023**, *44*, 200737. Faradizaji, B.; Borrego, E.I.; Jazi, M.E.; Smith, D.W., Jr. Triphenylene-Enchained Perfluorocyclobutyl (PFCB) Aryl Ether Polymers. A Modular Synthetic Route to Processable Thermoplastics Approaching Upper Limit Tg and Photostability. *Macromolecules* **2021**, *54*, 7666. Shelar, K.; Mukeba, K.; Mills, K., Smith, D.W., Jr. Renewable Isosorbide-Containing Semi-Fluorinated Aromatic Ether Polymers. *J. Polym. Sci. Part. A Polym. Chem*. **2022**, *60*, 2500–2507. Mukeba, K.M.; Shelar, K.E.; Faradizaji, B.; Borrego, E.I.; Caldona, E.B.; Pittman, C.U., Jr.; Smith, D.W., Jr. Semi-fluorinated Poly(aryl ether sulfone)s via Step-Growth Polymerization of Perfluorocyclohexene with Bisphenols. *Polymer* **2022**, *253* 124937.

### 9.6. Thermal Degradation of a Novel Series of Polyampholytes

Anuja Jayasekara ^1^^,^*, Ryo Shimada ^1^, Ayse Asatekin ^1^, Luca Mazzaferro ^1^, John Thomas ^1^, Peggy Cebe ^1^, Meghan Luebehusen ^2^, Gigi Zheng ^2^, Coco Xu ^2^ and Nathaniel van Gennip ^3^
^1^  Tufts University
^2^  Rochester Institute of Technology
^3^  Gallaudet University
*  Correspondence: anuja.jayasekara@tufts.edu

**Abstract:** Understanding thermal degradation processes is crucial in novel materials as it reveals their thermal stability with temperature. In this study we performed thermogravimetric (TG) analyses on a novel series of polyampholytes to identify their degradation mechanisms. Polyampholytes (PAs) are polymers with bulky monomer units that have both positively and negatively charged side groups. The newly synthesized series of PAs contains a hydrophobic unit in addition to the charged side groups. By analyzing Fourier-transform infrared spectra of the PAs which were annealed at various stages of the thermal degradation process, the degradation sequence was determined. Selected variants were electrospun to obtain fibrous mats. The fiber quality and average fiber diameter were determined from scanning electron microscope images.

### 9.7. Synthesis and Thermal Studies of High Performance 6F and 12F Polymers

Gustavo Munoz *, Maxim E. Solovyev, Kari Chamberlain, Charles Pittman and Dennis Smith
Mississippi State University
*  Correspondence: gam318@msstate.edu

**Abstract:** Polymers containing 1,1,1,3,3,3-hexafluoroisopropylidene (6F) in the backbone exhibit unique properties due to the strength of the C-F bond and the free volume this moiety generates. These properties include high thermal stability, a high glass transition temperature (Tg), improved solubility, enhanced optical transparency, among others. In this study, we introduce a direct strategy for incorporating the 6F group into polymers. The interfacial polymerization of hexafluoroacetone trihydrate (HFAH) with various activated and non-activated aromatic monomers was carried out using trifluoromethanesulfonic anhydride (Tf2O) and Aliquat^®^ 336 as phase transfer catalyst. This approach allows the synthesis of semi-fluorinated polyaryl ethers and polyphenylenes in high yield, with moderate to high molecular weights, minimal branching, and exceptional thermo-oxidative stability. Tg values ranging from 157–250 °C were obtained. High transmittance throughout the visible region was observed for these materials, along with low dielectric constant values. The developed methodology was successfully applied to both random and block copolymerization processes.

Furthermore, the same chemistry was used to synthesize 4,4′-bis(2-hydroxyhexafluoroisopropyl)diphenyl ether, a semi-fluorinated diol that incorporates 12 fluorine atoms per repeating unit. This monomer, under basic conditions, reacted with highly electrophilic functionalities such as acyl chlorides, carbonates, dichlorosilanes, and dichlorometallocenes to produce semi-fluorinated polyesters, polycarbonates, polysilylethers, and metallopolymers, respectively.

### 9.8. Comprehending the Structure of Ultrathin Polyzwitterionic Surfaces for Anti-Fouling

Charini Maladeniya ^1^^,^*, Panagiotis Christakopoulos ^1^, Ilia Ivanov ^1^, Liam Collins ^1^, Marti Nualart ^1^, Benjamin Doughty ^1^, Sun Pan ^1^, Marea Blake ^1^, Scott Retterer ^1^, Rajeev Kumar ^1^, Rigoberto Advincula ^2^
^1^  Oak Ridge National Laboratory
^2^  University of Tennessee—Knoxville
*  Correspondence: maladeniyacp@ornl.gov

**Abstract:** Polyzwitterions, which belong to a distinct category of polyampholytes, feature opposing charges on individual monomers and demonstrate remarkable antimicrobial and anti-fouling characteristics. This research introduces an effective method for chemically bonding thin layers of polyzwitterions onto solid surfaces to develop ultra-thin antimicrobial and anti-fouling surfaces. A sulfobetaine, specifically poly(2-vinylpyridine propane sulfone) (P2VPPS), was synthesized via free-radical polymerization. Concurrently, a photoreactive benzophenone derivative was covalently linked to the SiO2 substrate using a silane anchor. Ultrathin polyzwitterion films were produced by spin-coating the P2VPPS solution onto the benzophenone-functionalized substrate. To anchor the polyzwitterions onto the substrate, the films underwent UV exposure at 365 nm for various durations. Characterization of the ultrathin films was conducted using quartz crystal microbalance (QCM), atomic force microscopy (AFM), and sum-frequency generation (SFG). The impact of photochemical reactions and exposure duration on the structure and properties of these films will be explored.

### 9.9. Soybean Extract as a Promising Additive for Enhancing Surface Adhesion and Corrosion Protection of Fluoropolymer Coatings

**Marcel Roy Domalanta** **^1^** ^,^*, Delas Armas Emerson **^1^, Caldona** **^1^, Sanjida Ferdousi** **^2^  and Yijie Jiang** **^2^**
^1^  North Dakota State University
^2^  University of Oklahoma
*  Correspondence: marcel.domalanta@ndsu.edu

**Abstract:** Fluoropolymers have attracted the coating industry landscape mainly owing to their hydrophobic character and inherent chemical inertness. However, this nonreactive property, because of their low surface energy, has narrowed their applications, resulting in poor surface adhesion. Although research advancements have paved the way to provide strategies for improving fluoropolymer adhesion, their commercial utility is still limited by the sophistication and cost of these approaches. Extracts from fruits, leaves, and plants have been emerging as eco-friendly additives of interest for polymer coatings due to their abundance, sustainability, low toxicity, and tunable compatibility. Herein, we used soybean extracts (SE) as a green additive for poly(vinylidene fluoride-co-hexafluoropropylene) (PVDF-HFP) coatings for improved surface adhesion and corrosion protection. Results revealed increased fluoropolymer coatings’ affinity toward the metal surface. This enhanced surface adhesion may be credited to the ability of SE, characterized by abundant heteroatoms and pi-electrons from flavonoids, isoflavonoids, and anthocyanidins, to synergistically facilitate physical interactions, not only between the fluoropolymer chains within the coating matrix, but also at the coating/metal interface. Meanwhile, electrochemical results showed increased corrosion suppression in a highly concentrated salt solution, even at low doses of SE. These results were further corroborated by microscopic, spectroscopic, surface free energy, adhesion, thermal stability, and tensile tests, as well as computational and simulation studies to postulate the underlying coating performance mechanism. Overall, these low-cost and sustainable SE-containing fluoropolymer coatings are highly attractive for a wide range of versatile surface protection applications.

### 9.10. In-Situ Dielectric Analysis of Thermoplastic Polymers: Unveiling Thermal and Structural Dynamics

**Alec Redmann** **^1^** ^,^*, Nicholas Ecke **^2^, Alexander Chaloupka** **^2^, Natalie Rudolph** **^3^**
^1^  NETZSCH
^2^  NETZSCH Process Intelligence
^3^  NETZSCH Geraetebau GmbH
*  Correspondence: alec.redmann@netzsch.com

**Abstract:** Dielectric Analysis (DEA) is a well-established technique predominantly utilized for monitoring the curing process of thermosetting polymers. However, its application to thermoplastic polymers presents a novel approach with significant potential for advancing materials research. This study explores the in-situ characterization of thermoplastic polymers such as polypropylene (PP), polyamide (PA), and polycarbonate (PC) using DEA.

Through controlled heating and cooling cycles at a rate of 2 K/min, we observed distinct dielectric responses in both polar and non-polar polymers. These responses capture characteristic thermal and structural effects, providing insights into the behavior of these materials under varying thermal conditions. For instance, thermal aging of PA12 was evidenced by a shift in crystallization temperatures to lower values, highlighting DEA’s sensitivity to subtle microstructural changes.

Typical applications of DEA involve the real-time monitoring of curing processes in thermosets such as epoxies, polyesters, and polyurethanes, where it helps in optimizing production cycles by identifying significant processing parameters. Extending this technique to thermoplastics allows for the detection of phase transitions, such as glass transition and crystallization, which are crucial for understanding and improving the material properties and performance in practical applications.

Our findings underscore the versatility and high sensitivity of DEA in capturing the dynamics of thermoplastic polymers during thermal cycling. This approach not only broadens the scope of DEA but also provides a valuable tool for advancing the characterization and application of thermoplastic materials.

## 10. Session: Kinetics

### 10.1. Nucleation of Poly(pentylene adipate-co-terephthalate) Crystals with Various Ionomers

**Brian Grady** **^1^** ^,^*, Hesham Aboukeila **^1^, John Klier** **^1^, Emily Edwards** **^1^** **and George Huber** **^2^**
^1^  University of Oklahoma
^2^  University of Wisconsin
*  Correspondence: bpgrady@ou.edu

**Abstract:** Small amounts of various Zn, Li, Mg and Na-neutralized ethylene and methacrylic acid-based ionomers were used as nucleating agents for poly(pentylene adipate-co-terephthalate) (PPeAT). The mixtures were tested for isothermal and non-isothermal crystallization in a differential scanning calorimeter (DSC). The addition of 0.05 wt.% nucleating agent led to a decrease in crystallization half-time from 28 min to an average of 2.0, 2.3, 2.4, and 1.9 min for Zn, Li, Mg, and Na-based ionomers respectively. Moreover, increasing the amount of nucleating agent by weight led to a further decrease in crystallization half-time to 0.6 min at 0.5% by weight Na-based ionomer. This reduction in crystallization half-time allows PPeAT to possibly be a viable biobased biodegradable alternative to linear low-density polyethylene for flexible film packaging applications.

### 10.2. Cure-Kinetics Study for New Advanced Curing Structural Adhesives

Lang Wei *
Dupont
*  Correspondence: lang.wei@dupont.com

**Abstract:** The next generation DuPont™ BETAMATE™ broad bake adhesive technology is designed to meet OEM requirements for mechanical performance and adhesion strength in just 10 min baking at 140 °C, and can sustain the high temperatures up to 200 °C. By reducing bake times from 30 min to 10 min, this technology improves factory throughput and can even allow for smaller ovens in factories. To investigate the cure-kinetics of these adhesives, this study employed Differential Scanning Calorimetry (DSC) and successfully used the Kamal model to predict the curing degree and simulate the curing process.

### 10.3. Updating Critical Temperature Calculations of Several Secondary High Explosives

Jason Moore * and Steven Hawks
Lawrence Livermore National Laboratory
*  Correspondence: moore242@llnl.gov

**Abstract:** When designing experiments involving the heating of high explosives (HEs), it is vitally important to know if a thermal runaway is possible. This determination is often done by using the Frank-Kamenetskii (FK) equation.[1] The parameters necessary for this calculation are often referenced from decades-old literature, such as Rogers.[2] Many experiments used previously to estimate critical temperatures were limited to whether an explosion occurred during a single workday. However, more recent experimental data have shown that the values found from this method are often inaccurate, and in some cases overpredict the critical temperature by tens of degrees. Herein, we present updated parameters for several secondary HEs based on measurements from the LLNL one dimensional time to explosion (ODTX)[3] experiment and modeling using the ALE3D software package.[4]

**Acknowledgments:** This work was performed under the auspices of the U.S. DOE by Lawrence Livermore National Laboratory under contract DE-AC52-07NA27344. LLNS, LLC. LLNL-ABS-862041.

**References**

1. Frank-Kamenetskii, D.A. *Diffusion and Heat Transfer in Chemical Kinetics*; Plenum Press: New York, NY, USA, 1969.
2. Rogers, R.N. *Thermochim. Acta* **1975**, *11*, 131–139.
3. Hsu, P.C.; Hust, G.; Howard, W.M.; Maienschein, J.L. In 14th International Detonation Symposium, LLNL-CONF-425264, Coeur d’ Alene, ID, 2010; pp. 984–990.
4. Noble, C.; Anderson, A.; Barton, N.; Bramwell, J.; Capps, A.; Chang, M.; Chou, J.; Dawson, D.; Diana, E.; Dunn, T.; et al. LLNL-TR-732040, Lawrence Livermore National Laboratory, 2017.

### 10.4. Effects of Mass Transport on Thermal Decomposition Kinetics

**Alan Burnham** **^1^** ^,^*, Chiara Saggese **^2^, Batikan Koroglu** **^2^**
^1^  Stratify//MH Chew Associates
^2^  Lawrence Livermore National Laboratory
*  Correspondence: burnham1@llnl.gov

**Abstract:** Thermal analysis methods are widely used for measuring decomposition kinetics, but the precise conditions of the measurements are often not described, which makes it difficult to assess the validity of the kinetic parameters. One example is the role of diffusion resistance to the escape of generated products [1], which can slow the rate of evaporation and slow or enhance the rate of decomposition. The effect is particularly important for the thermal decomposition of energetic materials due to both sublimation in the absence of confinement and enhanced autocatalysis with confinement [2,3]. In this work, a variety of chemical systems are explored in reactor configurations ranging from open pans to hermetically sealed pans with the lid pierced with a pinhole of various diameter sizes. Processes examined are simple evaporation of eicosane, dehydration of Mg(OH)_2_, sublimation and decomposition of TATB and LLM-105, and decomposition of polyethylene. An equilibrium-limited mass transfer model coupled to simple chemical reaction models is developed and explored to show the sensitivity of the results and apparent kinetic parameters when mass transfer limitations are not properly included.

**References**

1. Saggese, C.; Burnham, A. Experimental and modeling study of the effect of confinement on the thermal decomposition of organic materials. *Thermochim. Acta* **2024**, 179741.
2. Koroglu, B.; Burnham, A.K.; Mashiana, I.; Racoveanu, A.; Arose, C.; Crowhurst, J.C.; Reynolds, J.G. Comparative analysis of sublimation and thermal decomposition of TATB. *Propellants Explos. Pyrotech*. **2024**. https://doi.org/10.1002/prep.202300124.
3. Burnham, A.K.; Stanford, V.L.; Vyazovkin, S.; Kahl, K.M. Effect of pressure on TATB and LX-17 thermal decomposition. *Thermochim. Acta* **2021**, *699*, 178908.

This work was performed under the auspices of the U.S. DOE by Lawrence Livermore National Laboratory under contract DE-AC52-07NA27344. LLNL-ABS-862219.

### 10.5. Kinetics and TTT Diagram for Curing of Epoxy-Amine System

Elena Moukhina * and Claire Strasser
NETZSCH Gerätebau GmbH
*  Correspondence: elena.moukhina@netzsch.com

**Abstract:** During the curing of epoxy systems, two processes happen: gelation and vitrification. Both of them have the influence on the physical properties of reacting material and on the curing rate. Both processes must be taken into account in the kinetic analysis.

For such a systems where reacting material changes its state from the viscose liquid over gelation to rubber, and then over vitrification to glassy state, the Time-Temperature-Transition (TTT) diagram is important. It can be used to optimize the time-temperature program for processing of investigated material.

Presented method builds the TTT diagram for amine-based epoxy resin, containing the glass transition temperature for fully uncured and fully cured sample as well as vitrification and gelation curves.

The created kinetic model has two steps and has diffusion-controlled reaction near the glass transition temperature Tg. This model is used for the simulation of the curing process in the industrial size, with simulation of coordinate-dependent temperature, degree of cure, curing rate, glass transition temperature and concentrations of al reactants and products.

Kinetic analysis was done in NETZSCH Kinetics Neo software, and the simulation was done in NETZSCH Termica Neo software, where both software packages are fully compatible and use the same calculation engine for the kinetic simulations.

### 10.6. Insights into Cure and Cure Kinetics from Simulated DSC Curves

Bruce Prime *
IBM (retired)
*  Correspondence: rbprime@sbcglobal.net

**Abstract:** Starting with the modified Kamal-Sourour equation below and incorporating the Arrhenius equation, conversion-time and rate of conversion-temperature curves were generated to simulate DSC curves by solving the differential equation numerically. For some cases and graphical display an Excel spreadsheet was utilized.

$$d\alpha /dt=(k1+k2\alpha^{m}){\left(1-\alpha \right)}^{m}$$

where α is conversion, dα/dt is the rate of conversion, k1 and k2 are rate constants, and m and n are reaction orders.

Sourour and Kamal (1976) developed their model to characterize the autocatalytic cure of an epoxy-amine system. k2 is the rate constant for the epoxy-amine reaction catalyzed by hydroxyl groups formed in the reaction and k1 represents the epoxy-amine reaction catalyzed by other moieties present, including added catalyst. An increasing k1 should therefore simulate this effect. Such addition was seen not only to speed up the reaction but also to alter its character in the direction of nth order kinetics. Differences observed between a 5-min epoxy and a 60-min epoxy (Judovits and Prime, 2015) can be explained by differing catalyst levels.

Separately, two sets of simulated DSC curves were analyzed with Netzsch Kinetics Neo software to test its ability to recover the kinetic parameters used to generate the curves. The first set was generated using the 2nd order kinetic equation found to describe the cure of a fast-reacting polyurethane system by employing isothermal and multiple heating rate DSC with time-temperature superposition. It was found that analysis of simulated DSC curves at four heating rates could generate a kinetic equation indistinguishable from the original equation and with greater accuracy. A second set of simulated DSC curves was generated utilizing the modified Kamal-Sourour equation with a moderate level of added catalyst. Kinetic analysis of these curves yielded the original kinetic equation and its parameters. It also demonstrated the ability of select model-free methodologies to generate conversion-time curves indistinguishable from the model-based analysis as well as the original data, but only the model-based analysis included the kinetic equation.

**References**

1. Judovits, L.; Prime, R.B. Polymer Innovation Blog. Available online: https://polymerinnovationblog.com/mtdsc-of-thermosets-part-6-sixty-minute-epoxy/ (accessed on 17 August 2015).
2. Prime, R.B.; Michalski, C.; Neag, C.M. *Thermochim. Acta* **2005**, *429*, 213.
3. Sourour, S.; Kamal, M. *Thermochim. Acta* **1976**, *14*, 41.
4. Appreciation is expressed to Dr. John Avila, San Jose State University (retired); Dr. Michael Prime, Los Alamos National Laboratory; and Dr. Elena Moukina, Netzsch who contributed to this work.

### 10.7. Kinetic Analysis of Thermoset Precursor-Derived Carbon Catalyzed by Organometallic Compounds

Faheem Muhammed *, Ngon Tran and Daniel B. Knorr
US Army Research Laboratory
*  Correspondence: faheem.h.muhammed.civ@army.mil

**Abstract:** It is established that thermoset carbon precursors yield sp2 hybridized carbon with structures that are not particularly well ordered (i.e., graphitic) after both pyrolysis (1000 °C) and heat treatment at temperatures exceeding 2000 °C under inert gas [1–3]. In this study, we demonstrate that the incorporation of organometallic catalysts (cobaltocene (CC), ferrocene (FC), and nickelocene (NC)) into a purified polybenzoxazine thermoset resin leads to significant increases in both crystallite thickness (LC) and width (LA), as confirmed by X-ray diffraction analysis. A deconvolution approach of the data obtained during pyrolysis utilizing Gaussian peaks was proposed to discern individual reactions within the multi-phased system (organometallic inclusion and polymer). Subsequent optimization facilitated the extraction of unique kinetic parameters (pre-exponential factor (A), activation energy (E), reaction order (n)), enabling quantitative analysis of the matrix mesoscale structure. Through this approach, we assessed the influence of catalysts on carbon matrix properties against pure resin (control) systems with identical thermal histories. The resulting larger crystallite sizes afforded by the organometallics indicate the production of a more graphitic form of carbon that exhibits enhanced oxidation resistance at high temperature due to reduced structural defects. Thermogravimetric analysis (TGA) under an oxidizing atmosphere was employed demonstrate this effect as a function of particle loading and LC.

**References**

1. Franklin, R.E. Crystallite growth in graphitizing and non-graphitizing carbons. *Proc. R. Soc. Lond. Ser. A Math. Phys. Sci.* **1951**, *209*, 196–218. https://doi.org/10.1098/rspa.1951.0197.
2. Harris, P.J.F. Non-Graphitizing Carbon: Its Structure and Formation from Organic Precursors. *Eurasian Chem. Technol. J.* **2019**, *21*, 3. https://doi.org/10.18321/ectj863.
3. Ouzilleau, P.; Gheribi, A.E.; Chartrand, P.; Soucy, G.; Monthioux, M. Why some carbons may or may not graphitize? The point of view of thermodynamics. *Carbon* **2019**, *149*, 419–435. https://doi.org/10.1016/j.carbon.2019.04.018.

### 10.8. Kinetics of Plasticized Poly(Vinyl Chloride) Thermal Degradation. Induction, Autocatalysis, Glass Transition, Diffusion

Vadim Krongauz *
ICU Medical, Inc.
*  Correspondence: vadim.krongauz@icumed.com

**Abstract:** Kinetics of non-oxidative thermal degradation of poly(vinyl chloride) (PVC) plasticized with di(2-ethylhexyl)phthalate and epoxidized linseed oil (ELO) were studied using differential scanning calorimetry. PVC thermal degradation autocatalyzed by forming HCl is delayed in the presence of acid scavenging ELO. Direct HCl elimination rates during steady state degradation (induction period) were almost independent on ELO concentration, with rate constants ranging from 3.2·10^−6^ s^−1^ at 463 K to 7.0·10^−5^ s^−1^ at 503 K. Activation energy of HCl elimination increased with glass transition temperature (Tg) increased from 136.0 kJ mol^−1^ at Tg = 260.3 K to 141.9 kJ mol^−1^ at Tg = 274.2 K. Diffusion coefficients of HCl diffusion in plasticized PVC deduced using Williams-Landel-Ferry equation were of the order of 10^−4^ cm^2^ s^−1^. Calculated activation energy of HCl diffusion in plasticized PVC was 30 kJ mol^−1^. The rate constants of PVC autocatalytic degradation were derived from the experimental data (for the 1st time) using Šesták-Berggren approach and ranged from 3.2·10^−2^ s^−1^ at 503 K to 4.4·10^−3^·s^−1^ at 463 K. Autocatalysis activation energies ranged from 70.4 kJ mol^−1^ to 85.1 kJ mol^−1^, and increased with glass transition temperature increase. It was established that PVC degradation kinetics was not controlled by HCl diffusion. Compensation effect was observed for initial and catalyzed PVC degradation.

### 10.9. Application of the Master Kinetics Approach for the Prediction of the Thermal Behavior of the Propellants Belonging to the Same Class of Materials

**Bertrand Roduit** **^1^** ^,^*, Damiano Rickenbach **^1^, Patrick Folly** **^2^, Alexandre Sarbach** **^2^, Marc Leubin** **^2^,** **Fabian Stucki** **^2^** **and Richard Baltensperger** **^3^**
^1^  AKTS SA
^2^  armasuisse
^3^  University of Applied Sciences of Western Switzerland
*  Correspondence: b.roduit@akts.com

**Abstract:** The assessment of the thermal stability of energetic materials (e.g., nitrocellulose-based propellants) very often begins with tests evaluating the stabilizer depletion, which is performed by artificial aging of the investigated material at a few temperatures (multi-temperature aging procedure [1,2]). Only a few points are collected at each temperature during the experimental procedure, whose duration is generally in the range of weeks or even months. This means that kinetic analysis based on only a few sparse data requires advanced specific mathematical and statistical treatment because the conventional kinetic procedure may result in imprecise or even incorrect results. This issue was discussed in detail in our previous study, published in 2014, which depicts an original kinetic analysis workflow dedicated explicitly to elaborating on the sparse data [3]. The method has been implemented in AKTS-Thermokinetics Software Version TKsd [4].

The proposed method has become a typical means of determining kinetic parameters from sparse data, particularly for assessing the service life of propellants [5] and all kinds of materials [6]. The current study aimed to modify the experimental and computational procedures to evaluate the kinetic parameters by introducing the ‘master kinetics’ approach. This procedure assumes that kinetic assessed data for one type of propellant can also be applied for other propellants belonging to the same class of materials, e.g., the same basic compounds and the same family of stabilizers. Applying the master kinetics approach to similar materials can significantly optimize and shorten the prediction of their thermal behavior, such as shelf life.

**References**

1. Brown, M.E.; Maciejewski, M.; Vyazovkin, S.; Nomen, R.; Sempere, J.; Burnham, A.; Opfermann, J.; Strey, R.; Anderson, H.L.; Kemmler, A.; et al. *Thermochim. Acta* **2000,** *355*, 125.
2. Vyazovkin, S.; Burnham, A.K.; Criado, J.M.; Pérez-Maqueda, L.A.; Popescu, C.; Sbirrazzuoli, N. *Thermochim. Acta* **2011**, *520*, 1.
3. Roduit, B.; Hartmann, M.; Folly, P.; Sarbach, A.; Baltensperger, R. *Thermochim. Acta* **2014**, *579*, 31.
4. AKTS-Thermokinetics Software (Version TKsd 6.02), AKTS SA, Technopôle 1, Sierre, Switzerland. Available online: https://www.akts.com (accessed on 17 May 2024).
5. Roduit, B.; Luyet, C.A.; Hartmann, M.; Folly, P.; Sarbach, A.; Dejeaifve, A.; Dobson, R.; Schroeter, N.; Vorlet, O.; Dabros, M.; et al. *Molecules* **2019**, *24*, 2216.
6. École Supérieure de Physique et de Chimie Industrielles.

### 10.10. Isothermal Crystallization and Time-Temperature-Transformation of Amorphous Indomethacin and Its Co-Amorphs

Elaheh A. T. Moghadam *, Sindee Simon
North Carolina State University
*  Correspondence: eabdoll@ncsu.edu

**Abstract:** Isothermal crystallization of indomethacin and binary miscible mixtures of indomethacin and sucrose benzoate samples are investigated from a super-cooled liquid state using differential scanning calorimetry to study the stability of the systems. These mixtures are termed co-amorphous glasses, and such materials have been shown to have enhanced water solubility and bioavailability relative to the crystalline forms of active pharmaceutical ingredients. The crystallization data for indomethacin as a function of temperature and time are modeled using the Avrami model. These data are also used to obtain the time-temperature-transformation (TTT) diagram, which is modeled using nucleation rate theory. In addition, the crystallization of indomethacin/sucrose benzoate co-amorphous materials is studied at 100 °C and fit to the Avrami model. The preliminary results show that co-amorphs having less than 70% indomethacin are more stable and take longer to crystallize than the pure material.

## 11. Session: Memorial Session for Prof. Bernhard Wunderlich

### 11.1. Comparison of PVDF Resins Made Using Different Surfactants

Larry Judovits *
ARKEMA (retired)
*  Correspondence: larry.judovits@hotmail.com

**Abstract:** This study is of powder and pellet samples of polyvinylidene fluoride (PVDF) made by emulsion polymerization using three different surfactants. The surfactants used contained different levels of hydrogenation. Each formulation differed in its ability to form a rarely observed high-temperature phase. Annealing just above the original melting temperature induced the high-temperature phase. The recipe with the highest hydrogen content was the most influenced. Differential scanning calorimetry (DSC) for all three formulations used a cycled temperature profile of 10 °C/min ramp rate. No significant difference between the formulations was observed with the reheat melting points ranging from 168 °C to 170 °C; however, this was not the case for the annealing pretreatments at approximately 175 °C. The formulations formed a high temperature peak after annealing, with an increased final melting peak that was 20 °C higher. For the pellet form, only the most hydrogenated form demonstrated, to any extent, the upper temperature melting peak after the pre-annealing treatment. For the powder form, all three formulations exhibited the upper temperature melting peak, with the most hydrogenated surfactant resin being the most enhanced in magnitude. Furthermore, DSC analysis found that with increasing molecular weight, the upper melting temperature peak decreased in magnitude for each formulation. Finally, associated with the formation of the high-temperature phase was a clarifying effect.

### 11.2. Liquid Heat Capacity of Amorphous Poly(Lactic Acid)

**Marek Pyda** **^1^** ^,^*, Marcin Skotnicki **^2^** **and Anna Czerniecka** **^3^**
^1^  University of Tennessee (retired)
^2^  University of Medical Sciences—Poland
^3^  Cracow University of Technology
*  Correspondence: mpyda@utk.edu

**Abstract:** The experimental and computed liquid heat capacity of amorphous poly(lactic acid) (PLA) was presented. The liquid heat capacity of PLA above the glass transition 333 K (60 °C) was linked to the molecular motions and computed as the sum of vibrational, external (anharmonic), and conformational contributions. The vibrational motion calculated as the group and skeletal vibrational heat capacity contributions was as most part of the liquid heat capacity, Cp (liquid) of PLA. The external contribution to Cp (liquid) was calculated as a function of temperature from experimental data of the thermal compressibility and expansivity of the liquid state. The contribution of conformational heat capacity to the total heat capacity of amorphous liquid PLA was calculated by fitting of the experimental, liquid heat capacity after subtracting the vibrational and external parts, to the obtained heat capacity based on a one-dimensional Ising-type model with two discrete states. These states characterize such parameters like stiffness, cooperativity, and degeneracy. The computed and experimental data of Cp(liquid) showed good agreement, at the investigated temperature region. The proposed approach can be applied for a determination of liquid heat capacities of more complex systems such as poly(lactic acid) -water or drug- which is widely interested in general or medical applications.

### 11.3. Cooling Calibration Aspects of Differential Scanning Calorimeters

Joseph Menczel *
Thermal Measurements LLC
*  Correspondence: jmenczel@yahoo.com

**Abstract:** Cooling calibration of DSC instruments is important for different types of experiments, first of all for crystallization. Since most materials exhibit supercooling when crystallized non-isothermally, it is difficult to find calibration standards for cooling. Menczel and Leslie (1990, 1993) introduced liquid crystals as temperature calibration standards for cooling experiments, because the nematic-to-isotropic transition of low molecular mass liquid crystals does not have hysteresis when cooled, the transition occurs at the same temperature on heating as on cooling. In refs. [1,2] it was also found that other liquid crystal-to-liquid crystal transitions also take place without hysteresis when these materials are heated and cooled.

Another type of transition that can be used for the cooling calibration is the Curie-transition, which is a second order equilibrium transition. However, the determination of the exact transition temperature in the DSC recordings is not fully clarified.

In this paper, we attempt to determine the transition temperature during a ferromagnetic transition by comparing it with the liquid crystal traces.

**References**

1. Menczel, J.D.; Leslie, T.M. *Thermochim. Acta* **1990**, *166*, 309
2. Menczel, J.D.; Leslie, T.M. *J. Thermal Anal*. **1993**, *40*, 957

### 11.4. Understanding Polymer Behavior: The Wunderlich Message

Michael Jaffe *
New Jersey Innovation Institute
*  Correspondence: jaffe@njit.edu

**Abstract:** It is almost 70 years since I left the Wunderlich RPI Research Group and started my research career. It took me years to recognize the gift Professor Wunderlich gave me was that Thermal Analysis was a route to understanding polymer (material) performance and that understanding behavior was different, and more important than, knowing about behavior. This academic background in polymer physics/thermal analysis led me to a successful R&D career ranging from industrial polymer product development to academic applied biology in the University. The details change, the rules do not. I will utilize thermoplastic fiber formation to illustrate the utility of TA to the understanding of complex materials processing. Utilizing multiple thermal analysis techniques, it is possible to correlate most aspects of fiber processing/properties with TA response, allowing for practical process-structure-property correlations. Utilizing similar techniques, it is possible to reverse engineer competitive products and identify process variable likely to produce similar performance profiles on a given processing platform. The integration of multiple TA and other characterization methods led to the formulations of models of structure formation during spinning that provided direction in the successful creation of new fiber products and process-structure-property concepts that are applicable to other polymer processing methods such as film formation and injection molding.

## 12. Session: Advanced Instrumentation & Tandem Techniques

### 12.1. Advantages of a Single Cell DSC Operated in Power Compensation Mode

Danielle Kimmel *, Matthias Wagner
METTLER TOLEDO
*  Correspondence: danielle.kimmel@mt.com

**Abstract:** Differential Scanning Calorimeters (DSC) running in power compensation mode have been known since their first development by O’Neil in 1964 [1]. In this approach, the power required to compensate for the heat of transition is measured. The commercially offered measurement systems usually consist of two small furnaces installed in a cooled metal block. This concept is available in both conventional DSC and fast DSC using chip calorimeters (Flash DSC) [2].

These instruments typically exhibit a short signal time constant, leading to excellent resolution of close-lying or fast effects.

A new type of power compensation DSC presented here has overcome the limitations regarding crucible or sample size, the challenges when measuring chemical reactions, the dependence on environmental influences, and the lack of automation robustness. A broad crucible portfolio is handled by standard automation in this device.

The DSC sensor has been enhanced with additional heating elements and temperature sensors for power compensation. In addition to the application of this measurement mode, the sensor heating elements enable performing electrical heat flow adjustment resulting in outstanding accuracy over a broad temperature range. The DSC can even switch between heat flux and power compensation mode.

The excellent performance of this device is demonstrated using quantitative performance measurements and advanced applications.

**References**

1. Watson, E.S.; O’Neill, M.J.; Justin, J.; Brenner, N. A differential scanning calorimeter for quantitative differential thermal analysis. *Anal Chem*. **1964**, *36*, 7–13.
2. Mathot, V.; Pyda, M.; Pijpers, T.; Vanden Poel, G.; van de Kerkhof, E.; van Herwaarden, S.; van Herwaarden, F.; Leenaers, A. The Flash DSC 1, a power compensation twin-type, chip-based fast scanning calorimeter (FSC): First findings on polymers. *Thermochim. Acta* **2011**, *522*, 36–45.

### 12.2. Insights into Food Processing Physics and Chemistry with Thermal Analysis and Microscopy

Danielle Kimmel *, Matthias Wagner and Markus Schubnell
METTLER TOLEDO
*  Correspondence: danielle.kimmel@mt.com

**Abstract:** We will demonstrate the usefulness of simultaneous microscopy DSC as well as other thermal analysis techniques for the interpretation of thermal behavior using three food sample types.

The measurement of Aspartame, an artificial sweetener, exhibits (1) the release of water of crystallization and (2) two sharp endothermic peaks at higher temperatures that cannot be easily explained. Microscopy simultaneous to the DSC measurement revealed which effects can be assigned to the peaks.

Utilization of thermal analysis techniques to probe the thermal decomposition of sucrose shows the narrow temperature boundaries between successful caramelization and charring. Simultaneous microscopy delivers valuable information on the various sub-processes that occur upon heating.

The roasting of the green coffee beans, during which several different processes take place, is crucial for the taste of brewed coffee. Many of the small roasting plants rely on the empirical know-how of single persons for controlling the roasting process. Thermal analysis can help to optimize the conditions with quantifiable parameters. We were able to identify various distinct processes with the help of thermomechanical analysis (TMA) and DSC microscopy.

### 12.3. Monitoring Curing Using QiDSC and Pycnometry

Larry Judovits *
ARKEMA (retired)
*  Correspondence: larry.judovits@hotmail.com

**Abstract:** Modulated temperature DSC (MT-DSC) is typically used to monitor the cure of epoxies. Quasi-isothermal DSC (QiDSC) is an MT-DSC technique where a sinusoidal temperature oscillation is performed around a set temperature. MT-DSC can separate the total heat flow into its reversing and nonreversing components, while for QiDSC, only the reversing heat flow can be separated from the total heat flow. As the epoxy hardens, the apparent heat capacity changes as the material cures over time. Gas pycnometry determines the volume change by which the curing can be monitored. It does this by employing the ideal gas law to relate the change in sample dimension over time to the resulting pressure differential.

After mixing a two-part epoxy, the material hardens, with the glass transition increasing as the reaction proceeds. MT-DSC can observe this in two ways. First, one can cure specimens in the DSC cell for various times at a constant temperature and then analyze the heating by MT-DSC, determining the Tg through the reversing heat flow signal. Another method is to use QiDSC and determine on a time basis when the glass transition passes the curing temperature through the analysis of the reversing heat flow signal. A limitation of the DSC methodology is that only small sample sizes can be used, therefore not providing the self-generated heat that may be necessary for the reaction to proceed. Pycnometry allows much larger sample sizes, up to gram amounts, therefore allowing reactions to proceed if self-heating is necessary. For this presentation, both QiDSC and pycnometry are compared for various systems. A pycnometry example is given for the curing of a bulk free radical polymerization that MT-DSC was not able to monitor since self-heating was necessary.

### 12.4. Using Thermal Analysis in Elastomer Compatibility Studies

Tina Adams *
The Lubrizol Corporation
*  Correspondence: tina.adams@lubrizol.com

**Abstract:** When performance issues arise involving elastomeric materials and fluids, but you are sample limited and cannot utilize standard industry tests that require large specimens, where do you turn? When the variables are numerous enough that many specimens are needed for a statistically representative design of experiments, how do you plan? Thermogravimetry (TGA) and differential scanning calorimetry (DSC) are the answers. They utilize exceedingly small specimens and are sensitive to subtle changes.

This presentation covers how TGA along with temperature modulated DSC are keys to not only discovering the nature of a failure involving elastomeric materials and fluids in many environments but also to delving into the fundamental knowledge needed to improve future formulating and applications. The techniques allow for many analyses on small sample sizes. They are applicable to both polymeric and fluid analyses. And the data can be expressed in many formats to create business compelling communications. Custom sample treatment, thermal stability determination, and transition analyses have uncovered the traits of an experimental elastomer and identified its weaknesses in performance and in chemical compatibility.

### 12.5. Enhanced Characterization of Polymers and Composite Materials Utilizing Axial-Torsional Dynamic Mechanical Analysis

**Gunther Arnold** **^1^, Jose Alberto Rodriguez Agudo** **^1^, Jan Haeberle** **^1^, Christopher Giehl** **^1^,** **Jörg Läuger** **^1^** **and Abhishek Shetty** **^2^**
^1^  Anton Paar Germany
^2^  Anton Paar USA
*  Correspondence: gunther.arnold@anton-paar.com

**Abstract:** Rheometers or dynamic mechanical analyzers that are able to combine two drive units (rotational and linear drive) have opened new horizons in the characterization of novel composite materials [1,2]. For example, the combination of both linear and rotational measurement drive enables the determination of the complex Young’s modulus |E*| and the complex shear modulus |G*| for a single sample in one continuous measurement run. Additionally, the setup allows to include frequency, temperature and humidity sweeps in the measurements. This type of multi-task measurements is interesting for isotropic materials as they offer an indirect method to determine the viscoelastic Poisson’s ratio and its dependence on time and temperature [3–5]. In the case of anisotropic materials like composite materials, they are particularly useful since it is possible to evaluate, within one single run, extensive information on the viscoelastic properties of the material in both longitudinal and transversal directions.

In this contribution several application examples are shown in which axial-torsional measurements are combined in one single experiment to obtain a more complete information about the thermo-mechanical response of anisotropic materials. The examples show that the ratio between E* and G* of anisotropic materials differs from the ratio of isotropic materials made of the same matrix materials. Furthermore, the results indicate that the ratio between E* and G* of anisotropic materials is highly temperature dependent. A new parameter, ζ = tanΔG/tanΔE is calculated directly from the phase shift angle in extensional (ΔE), and torsional (ΔG) direction. This parameter appears as an indicator of anisotropy of composite materials and offers a novel dynamic fingerprint that augments the viscoelastic characterization of anisotropic materials. The results indicate the strong dependence of material properties on load direction for anisotropic materials and demonstrate the additional benefit of combined axial-torsional measurements for a comprehensive characterization of currently used materials.

**References**

1. Jiao, D.; Lossada, F.; Guo, J.; Skarsetz, O.; Hoenders, D.; Liu, J.; Walther, A. Electrical switching of high-performance bioinspired nanocellulose nanocomposites. *Nat. Commun*. **2021**, *12*, 1312.
2. Meurer, J.; Rodríguez Agudo, J.A.; Zechel, S.; Hager, M.D.; Schubert, U.S. Quantification of Triple-Shape Memory Behavior of Polymers Utilizing Tension and Torsion. *Macromol. Chem. Phys*. **2021**, *222*, 2000462.
3. Rodríguez Agudo, J.A.; Haeberle, J.; Müller-Pabel, M.; Troiss, A.; Shetty, A.; Kaschta, J.; Giehl, C. Characterization of the temperature and frequency dependency of the complex Poisson’s ratio using a novel combined torsional-axial rheometer. *J. Rheol*. **2023**, *67*, 1221.
4. Müller-Pabel, M.; Rodríguez Agudo, J.A.; Gude, M. Measuring and understanding cure-dependent viscoelastic properties of epoxy resin: A review. *Polym. Test*. **2022**, *114*, 107701.
5. Kim, Y.S.; Rodríguez Agudo, J.A.; Wistuba, M.P.; Büchner, J.; Schäffler, M. Determination of Complex Poisson’s Ratio of Asphalt Binders Using Torsion-Tension Tests in a Dynamic Shear Rheometer. *Road Mater. Pavement*. **2024**, *accepted*.

### 12.6. Temperature Dependent Structural Transitions in Highly Oriented Nylons with Symmetric Alkane Segments

Logan Kearney *, Michael Toomey and Amit Naskar
Oak Ridge National Laboratory
*  Correspondence: kearneylt@ornl.gov

**Abstract:** The Nylon family bearing symmetric alkane segment distances (2n 2[n + 1]), such as Nylon 4 6, exhibit exceptional properties as compared to other more common engineering grates (Nylon 6 6, Nylon 12, etc.). This can be attributed to the intensification of amide hydrogen bonding and the accompanying cohesive energy density. The interplay between the hydrogen bonding sheet formation and segmental stiffness template the procession of chain folding from the melt and result in unique crystal phases. Within this study we will present our work on mapping the Brille transition and process induced structural order for highly oriented Nylon 4 6 that has been drawn into fiber form. Calorimetry serves to give a foundational basis for these studies and is used to inform operando X-ray scattering experiments where the applied stresses and temperature are controlled independently. We correlate these thermal transitions to the semi-crystalline morphology to unlock their function and inform future high performance materials design.

### 12.7. Simplifying the Identification of DSC Curves

Yanxi Zhang *
NETZSCH Instruments North America
*  Correspondence: yanxi.zhang@netzsch.com

**Abstract:** Differential Scanning Calorimetry (DSC) is a very common thermal analysis technique. Interpretation of DSC curve requires a lot of experience and time by looking up in literature or consulting with experts. Thanks to “Identify” software, interpretation of DSC curve is not a tough job anymore, can do with a single mouse click.

“Identify” is a unique thermal analysis database system for the recognition and comparison of measurement curves of DSC, TGA, DIL/TMA as well as data on the specific heat capacity, Cp. The database includes more than 2500 entries from the fields of ceramics and inorganics, metals and alloys, polymers, organics, food and pharmaceuticals, as well as chemical elements. “Identify” software offers an automatic DSC curve recognition of unknown measurements and thus interpretation via database search, which is in general be very helpful for material identification, failure analysis and quality control. The database can expand by users’ own libraries shared with several users in the computer network. Identify can serve as archiving system too, for data mining since evaluated curves can restore in database easily.

Limitation of any database search is that curve recognition is not automatically material recognition. Sometimes multiple interpretation is possible. Under that condition, not only the best hit with one signal considers, but also several signals such as DSC, TGA etc. “Identify” software develops to use with combined TGA and DSC, TGA-c-DTA and STA measurements, which allows for material identification with greater certainty.

## 13. Session: Fast Scan Techniques/Nanocalorimetry

### 13.1. Influence of Molecular Structure on the Cooperative Fluctuation in the Vicinity of the Glass Transition

**Jürgen Schawe** **^1^** ^,^*, Allisson Saiter **^2^, Kylian Hallavant** **^2^** **and Antonella Esposito** **^2^**
^1^  ETH Zurich
^2^  Université de Rouen—Normandy
*  Correspondence: juergen.schawe@mat.ethz.ch

**Abstract:** In a supercooled glass-forming melt the spatio-temporal domains are formed that determine the characteristic time of the long-range molecular rearrangements. These domains are the Cooperative Rearrangement Regions (CRR). During the glass transition upon cooling the CRR freeze and thus reduce the heat capacity of the glass. The average size of the CRR, Ξ, can be determined from the shape of the calorimetrically measured glass transition. How the size of the CRR depends on the molecular structure has been widely and controversy discussed. In such investigations, the molecular structure is usually varied, which than also leads to changes in the glass transition temperature, Tg, and the fragility index, m.

In this contribution, three materials with different molecular structure but similar Tg and m are selected. These are the two polymeric glass formers poly(vinyl acetate) (PVAc) and poly(lactic acid) (PLA) as well as the chalcogenide glass former selenium. The kinetics of the glass transition of these materials is determined the cooling rate dependency of the limiting fictive temperature T’ measured by DSC and fast differential scanning calorimetry (FDSC) using Flash DSC 2+ (METTLER TOLEDO). The heat capacities of the glass and the supercooled melt are measured using stochastically temperature modulated DSC. After correction the influence of the thermal inertia on the heat flow curves, the characteristic length Ξ is determined according to the approach of Donth [1].

It was found that the characteristic length Ξ decreases with increasing temperature. The relation between Ξ and the reduced temperature T/Tg show no significant differences between the investigated materials. In conclusion, the size of the dynamic heterogeneities in a supercooled melt in the vicinity of Tg depends on the macroscopic kinetics of the glass transition and is not directly influenced by the chemical composition and structure [2].

**References**

1. Chua, Y.Z.; et al. Determination of the cooperative length in a glass forming polymer. *ACS Phys. Chem. Au*. **2023**, *3*, 172–180.
2. Hallavant, K.; Mejres, M.; Schawe, J.E.; Esposito, A.; Saiter-Fourcin, A. Influence of chemical composition and structure on the cooperative fluctuation in supercooled glass-forming liquids. *J. Phys. Chem. Lett.* **2024**, *15*, 4508–4514.

### 13.2. Reproducible Cycling of Phase-Change Material via Nanocalorimetry

**Jie Zhao** **^1^** ^,^*, Zichao Ye **^1^, Leslie Allen** **^1^, Chih-Yu Lee** **^2^, Carlos A. Ríos Ocampo** **^2^, Ichiro Takeuchi** **^2^** **and Mikhail Y. Efremov** **^3^**
^1^  University of Illinois—Urbana-Champaign
^2^  University of Maryland
^3^  University of Wisconsin –Madison
*  Correspondence: jiezhao5@illinois.edu

**Abstract:** Optical phase change materials (OPCMs) have garnered significant interest in a variety of photonic applications including photonic integrated circuits, structural color, metasurfaces, optical metamaterials, and plasmonics [1]. Sb_2_Se_3_, a low-loss OPCM in the telecommunication spectrum has been gaining space in this field owing to its reliable response and large bandwidth [2]. OPCMs rely on heat stimuli to switch between their low and high refractive index states, i.e., amorphous and crystalline states, respectively. Therefore, measuring their thermal metrics (heat capacity, transition enthalpy/entropy) with high precision is critical to reveal its intricate switching mechanism.

In this study, we use thin-film Nanocalorimetry [3–6] to probe the phase transition of 20 nm thick thin-film Sb_2_Se_3_. For the first time, we demonstrated reproducible cycling of 20 nm thin-film Sb_2_Se_3_ between amorphous and crystalline phases with a scanning rate up to 900,000 K/s. Such reproducibility highlights Nanocalorimetry’s ability to mimic the device operation and provides practical information to achieve spatial and temporal control of glassy-crystalline distribution. It also mitigates concerns about the transferability of results obtained from model systems to its application, due to, for example, the difference between as-deposited and melt-quenched amorphous states.

**References**

1. Huang, Y.S.; Lee, C.Y.; Rath, M.; Ferrari, V.; Yu, H.; Woehl, T.J.; Ni, J.H.; Takeuchi, I.; Ríos, C. Tunable structural transmissive color in fano-resonant optical coatings employing phase-change materials. *Mater. Today Adv.* **2023**, *18*, 100364.
2. Lee, C.Y.; Lian, C.; Sun, H.; Huang, Y.S.; Acharjee, N.; Takeuchi, I.; Ríos Ocampo, C.A. Spatial and temporal control of glassy-crystalline domains in optical phase change materials. *J. Am. Ceram. Soc.* **2024**, *107*, 1543–1556.
3. Lai, S.L.; Guo, J.Y.; Petrova, V.; Ramanath, G.; Allen, L.H. Size-dependent melting properties of small tin particles: nanocalorimetric measurements. *Phys. Rev. Lett.* **1996**, *77*, 99.
4. Efremov, M.Y.; Olson, E.A.; Zhang, M.; Zhang, Z.; Allen, L.H. Glass transition in ultrathin polymer films: calorimetric study. *Phys. Rev. Lett.* **2003**, *91*, 085703.
5. Zhao, J.; Hui, J.; Ye, Z.; Lai, T.; Efremov, M.Y.; Wang, H.; Allen, L.H. Exploring “No Man’s Land”—Arrhenius Crystallization of Thin-Film Phase Change Material at 1,000,000 K^−1^ via Nanocalorimetry. *Adv. Mater. Interfaces* **2022**, *9*, 2200429.
6. Zhao, J.; Khan, A.I.; Efremov, M.Y.; Ye, Z.; Wu, X.; Kim, K.; Lee, Z.; Wong, H.S.P.; Pop, E.; Allen, L.H. Probing the melting transitions in phase-change superlattices via thin film nanocalorimetry. *Nano Lett.* **2023**, *23*, 4587–4594.

### 13.3. Crystallization Kinetics and Polymorphism in PA66/6I6T Blends: Influence of Chain Mobility and Temperature

Xiaoshi Zhang ^1^^,^*, Alicyn Rhoades ^1^, Ping Yi Leslie Poh ^1^ and John Buzinkai ^2^
^1^  Penn State—Behrend
^2^  INVISTA Nylon Polymer
*  Correspondence: xvz5402@psu.edu

**Abstract:** This study investigates the crystallization kinetics of polyamide 66 (PA66) blended with an amorphous polyphthalamide random copolymer, 6I6T, in a temperature range relevant to injection molding conditions. Blends were prepared with three different weight contents of 6I6T: 10 wt.%, 20 wt.%, and 30 wt.%. Additionally, two types of 6I6T with different molecular weights were utilized to understand their effects on the crystallization behavior of PA66. Our findings reveal that the addition of the amorphous copolymer significantly slows down the crystallization rate of PA66. Interestingly, the polymorphism of PA66, characterized by the formation of mesophase and α-phase structures, is not greatly affected by the presence of 6I6T. However, the molecular weight of 6I6T plays a crucial role in the crystallization kinetics within the mesophase region, while its impact on the α-phase formation remains negligible. Thermal characterization was conducted using fast scanning calorimetry and rotational rheometry to evaluate the crystallization kinetics and chain mobility. The reasons leading to the drastic differences in crystallization kinetics of the blends in the mesophase region will be discussed in detail.

### 13.4. A Novel and Robust Method for Automated Heat Capacity Analysis of Polymeric Calorimetric Data

John Thomas * and Peggy Cebe
Tufts University
*  Correspondence: john.thomas@tufts.edu

**Abstract:** The determination of thermal properties in polymers relies heavily on precise calorimetric measurements, yet ultimately it is necessary for human intervention to identify transition regions. This often occurs through a subjective assessment or “eye test.” Techniques such as differential scanning calorimetry (DSC) or fast scanning DSC (FSC) are used to measure key dynamics such as the glass transition or crystalline melt. However, these dynamics often span a large temperature range, making the pinpointing of their onsets challenging. The significance of the “eye test” becomes apparent in this context, as users manually select the onset and completion of transitions separating them from regions of steady state behavior. This selection process allows for commercial instrument-based software to then calculate the glass transition temperature (Tg), the heat capacity increment at Tg (Δcp), the heat of fusion (ΔHf), and peak melt temperature (Tm). While the manual selection process is crucial to identifying these transitions, this “eye test” is inherently subjective and can lead to high variability. This reliance on human judgement poses a challenge to both consistency and reproducibility emphasizing the need for automation for this critical process. To address this, we developed a robust automated method for heat capacity analysis of polymeric systems, capable of determining these key properties without the need for user input.

### 13.5. The Crystallization and Rigid Fraction of PLLA

Logan Williams * and Sindee Simon
North Carolina State University
*  Correspondence: lwilli25@ncsu.edu

**Abstract:** Fast scanning calorimetry (Flash DSC) is used to study the crystallization kinetics for a model polymer, poly(L-lactic acid) (PLLA) and the concomitant development of the so-called rigid amorphous phase or RAF, which we refer to as simply as the rigid fraction (RF) given the debate over its nature. Contrary to recent studies, a rigid fraction of 15–20% forms during crystallization for both a’ and a polymorphs of PLLA. Kinetic modeling using the Avrami model is used to study the development of both the crystallization and the formation of the RF.

### 13.6. Investigation of Commercial Isotactic Polypropylene Fibers Using Conventional DSC and Flash DSC

Yung P. Koh * and Sindee Simon
North Carolina State University
*  Correspondence: ykoh@ncsu.edu

**Abstract:** The thermal properties of semi-crystalline polymeric fibers, such as their glass transition and melting temperatures, heats of crystallization and melting, as well as their degree of crystallinity and the fraction of mobile and rigid amorphous phases, dictate their behavior both in post-extrusion processing steps, as well as in use. However, measurement of the fiber properties using conventional DSC can be complicated by fast crystallization kinetics and reorganization, and this problem is particularly difficult in the case of isotactic polypropylene (i-PP) fibers. Flash DSC allows the suppression of such reorganization through the use of high scanning rates, up to 30,000 K/s, allowing us to clearly measure the fractions of crystal, mobile amorphous, and rigid amorphous fractions in pristine i-PP fibers. Interestingly in comparing two commercial fibers, we find that although the degree of crystallinity is the same, the fraction of the mobile amorphous phase appears to be directly related to the strength of the resulting nonwoven mat, presumably because the thermal point bonding process is more effective when the mobile amorphous fraction is higher.

### 13.7. FSC-Empowered Multi-Technique Analysis of the Crystallization Kinetics and Resulting Microstructure in Engineering Thermoplastics

**Alicyn Rhoades** **^1^** ^,^*, Xiaoshi Zhang **^1^** **and John Buzinkai** **^2^**
^1^  Penn State—Behrend
^2^  INVISTA Nylon Polymer
*  Correspondence: amh234@psu.edu

**Abstract:** Processing of thermoplastics during injection molding and blow molding usually includes rapid cooling with rates up to 1000 K/s and solidification at high supercooling. In this work, we expand the existing capability of FSC by coupling it with other techniques, including micro-IR spectroscopy (Micro-IR), atomic force microscopy (AFM), polarized optical microscopy (POM), and X-ray computed tomography (XCT). Polymorphism and morphology transition associated with processing conditions will be discussed in polyamide 66, polyamide 6, poly (ether ether) ketone and its composites, including glass-filled and carbon-filled PEEK. Ultimately, a more accurate simulation of plastic solidification can be achieved using insights from fast scanning calorimetry and coupled techniques.

## 14. Session: Electronic Packaging Materials

### 14.1. Thermal Analysis of Materials Used in Microelectronics

Karl F. Schoch, Jr. *
Northrop Grumman
*  Correspondence: k.eric.schoch@ngc.com

**Abstract:** The techniques of thermal analysis, including DSC, TMA, TGA, DMA and rheology are widely used when selecting materials for electronic assemblies and the processes by which they are built. Those methods are used for determination of physical properties, such as glass transition temperature, thermal expansion, and modulus. Those properties are used in developing mechanical models and thermal models to predict the performance of hardware and the useful life of the hardware. In addition, thermal analysis methods are used to evaluate options for cure conditions, determination of working life of thermosets, dispense characteristics, and storage life of raw materials.

This paper will discuss two important topics that can be overlooked when characterizing materials for electronic assemblies: (1) measurement and management of shrinkage and stress during cure of encapsulants and coatings and (2) issues related to thermal interface materials. Thermosets shrink during cure by 3 to 10%, which can lead to void formation, cracking, stress on encapsulated parts, delamination and warping. This phenomenon is well known and has been the subject of considerable research in past 60 years or more. In this paper measurement of cure shrinkage by DMA, simultaneous with determination of gel point, will be discussed. Although cure stress can also be measured by DMA, the paper will describe use of a film stress tool to monitor stress in a thermoset during cure and subsequent thermal cycling. The results of this testing can be used to optimize cure conditions to minimize cure stress and also determine the “zero stress” condition used in mechanical modeling of these assemblies.

Thermal interface materials (TIMs) are used to assist in heat dissipation in electronic assemblies. As the hardware dissipates more power in a smaller volume, thermal management becomes more challenging. There are several types of TIMs, and this paper will describe characterization methods used for phase change materials, elastomeric pads, and “gap fillers”, which are putties that can be either non-curing or cured in place. Thermal conductivity and thermal resistance are key properties for any TIM, and each type has other properties that are important. For example, the softening point and handling characteristics of a phase change material are especially important. Elastomeric pads have a minimum amount of compression required for stable thermal properties and a maximum amount of compression that is possible. They may relax and loose contact with the hardware in use. The rheological properties of putties are important for choosing dispense conditions to minimize damage to hardware during assembly and to ensure good thermal performance during the life of the hardware. The properties are rate dependent and depend on the geometry of the hardware. The paper will show examples of these issues and how we use thermal analysis methods to address them.

### 14.2. Prediction of Thermal Stability of Epoxy Resin by Modified Kinetic and Model Selection Approaches Based on Limited Amount of Experimental Points

**Bertrand Roduit** **^1^** ^,^*, Patrick Folly **^2^, Alexandre Sarbach** **^2^** **and Richard Baltensperger** **^3^**
^1^  AKTS SA
^2^  armasuisse
^3^  University of Applied Sciences of Western Switzerland
*  Correspondence: b.roduit@akts.com

**Abstract:** The study of the ageing of electronic packaging materials at room temperature is experimentally very difficult due to its very low rate, the minimal changes in physicochemical properties and, very often, the limited amount of experimental data. Commonly applied methods of thermal-aging determination are therefore based on kinetic analysis carried out by measuring material properties at several elevated temperatures. In this study we propose a modification of the kinetic approach in the case of the accessibility of limited amount of experimental data without previous assumptions on the reaction mechanism [1]. In proposed modification both, single- and multi- steps kinetic models are applied, what results in much better fit of experimental data. The method used to compare different models takes into account not only the quality of regression fit, but also the number of data points and number of parameters in specific models based on information criterion such as Akaike (AIC). Due to considering regression parameter “w” such procedure allows concluding not only which model is more likely to be correct but even quantifying how much more likely. The proposed method delimits also the borders of the prediction band (e.g., 95% confidence) based on the bootstrap method showing scatter of the data and allows considering uncertainty of the best-fit curve being very important for thermal aging predictions. The proposed method was applied to determine thermal ageing by focusing on the long-term adhesion strength of various two-component epoxy systems with thermal cycling [2]. This analysis aimed to understand how thermal fatigue develops. The results provide valuable insights into the effects of temperature increases in power electronic applications, which often lead to decreased lifetime and reliability.

**References**

1. AKTS-Thermokinetics Software (Version TKsd 6.02), AKTS SA, Technopôle 1, Sierre, Switzerland. Available online: https://www.akts.com (accessed on 17 May 2024).
2. Kolbe, J. adhäsion KLEBEN DICHTEN. **2021**, *1–2*, 32.

### 14.3. Correlation of Underfill Materials Thermo-Mechanical Behavior with Advanced Packaging Stress and Reliability

Kaysar Rahim *
Northrop Grumman Mission Systems
*  Correspondence: md.rahim@ngc.com

**Abstract:** Underfill encapsulation helps to reduce the CTE mismatch between the flip chip die and the organic laminate substrate. It also helps to distribute and minimize the solder joint strains. Advanced Packaging assembly uses Underfill encapsulation to improve the thermo-mechanical fatigue life of electronic components. Underfill delaminations is one of the common failure modes in Advanced Packaging due to harsh operating conditions. Any delaminations that occur at the underfill/die interface will propagate to the neighboring solder bumps and lead to solder joint fatigue and failure. The onset and propagation of delaminations in flip chip assemblies exposed to temperature cycling are governed by the cyclic stresses and damage occurring at the underfill to die interface. For this reason, underfills are optimized by increasing their adhesion strength, interfacial fracture toughness, and resistance to thermal aging.

In this work, the thermo-mechanical behavior of three different underfill materials have been characterized and correlated directly to the packaging stress. A fundamental understanding of delamination initiation and growth in flip chip assemblies through simultaneous characterization of the stress and delamination states at the die to underfill interface have been developed and reported. The packaging stresses have been characterized during temperature cycling tests and correlated with the delaminations occurring at the die passivation to underfill interface. Using the measurements and numerical simulations, valuable insight has been gained on the effects of assembly variables and underfill thermo-mechanical material properties on the reliability of Advanced Packages.

**References**

1. Rahim, K.; Suhling, J.C.; Copeland, D.S.; Jaeger, R.C.; Lall, P. Measurement of stress and delamination in flip chip on laminate assemblies. In Proceedings of IPACK03 International Electronic Packaging Technical Conference and Exhibition, Maui, HI, USA, 6–11 July 2003.
2. Rahim, K.; Suhling, J.C.; Jaeger, R.C.; Lall, P. Fundamentals of Delamination Initiation and Growth in Flip Chip Assemblies. In Proceedings of the 2005 Electronic Components and Technology Conference, Lake Buena Vista, FL, USA, 31 May–3 June 2005.

### 14.4. Measurement and Prediction of Cure Kinetics, Gel Point, and Cure Stress of Underfill

Ran Tao *, Stian Romberg and Amanda Forster
National Institute of Standards and Technology
*  Correspondence: ran.tao@nist.gov

**Abstract:** Underfill is an important class of advanced semiconductor packaging material. These materials are typically epoxy-based thermosetting polymers that are applied to fill the gap between the semiconductor chip (die) and the substrate (such as a printed circuit board). Once cured, the underfill adds support, improving reliability and helping to distribute thermal and mechanical stress, which is crucial for preventing failure in delicate solder connections. During application, liquid underfill is dispensed around the edges of the die, after which it flows beneath the die by capillary action. It is then cured at elevated temperatures to achieve the desired thermo-mechanical properties. In this study, we investigate a widely used underfill material with complex reaction kinetics. We employ a comprehensive approach to provide a fundamental understanding of its reaction kinetics, cure-dependent rheology behaviors, and cure stress dependence on different cure schedules. Specifically, we develop a multi-step kinetics model to predict the degree of cure, and a semi-empirical chemoviscosity model to predict viscosity as a function of time and temperature, an advanced rheology technique called optimally windowed chirp measurements to measure the gel point, and finally, we use a film stress analyzer to measure cure and thermal stresses.

### 14.5. Thermal and Mechanical Characterization of Potting Materials for Electric Vehicle Applications

Arya Hakimian *
C-Therm Technologies Ltd.
*  Correspondence: arya.hakimian@ctherm.com

**Abstract:** Potting, a widely adopted technique for safeguarding electronic components, plays a crucial role in high-voltage applications like electric vehicle (EV) motors and batteries. By encapsulating components in protective materials, potting provides mechanical, thermal, and environmental benefits. To enhance performance, manufacturers often add fillers to the potting material. However, achieving uniform filler dispersion within the potting material is essential to prevent reduced performance, shorter lifespan, and the risk of thermal runaway due to agglomeration or sedimentation. Additionally, avoiding air entrapment during the potting process is critical for maintaining overall quality.

In this context, C-Therm Technologies offers innovative solutions for the thermal characterization of potting materials. Leveraging methods such as the FLEX Transient Plane Source (TPS) and Modified Transient Plane Source (MTPS) available on the Trident platform, these techniques enable fast test times, allowing accelerated feedback during material development. Notably, the single-sided MTPS can thermally map the material, detecting filler non-uniformity and air voids.

From a mechanical perspective, Dynamic Mechanical Analysis (DMA) proves valuable. By measuring the frequency dependence of the modulus under different conditions, DMA provides insights into material behavior. Low frequencies relate to molecular weight, while high-frequency measurements reveal events like impacts and drive train vibrations. Metravib’s line of high-force DMA instruments uniquely qualifies for exploring potting material performance for R&D and quality assurance.

### 14.6. Analysis of High Thermal Conductivity Liquid Dispensed Silicone Gap Fillers for Use in Manufacturing

Adam Karges * and Craig Whitehead
Momentive Performance Materials
*  Correspondence: adam.karges@momentive.com

**Abstract:** Liquid dispensed thermally conductive silicone gap fillers are used in a wide variety of electronic assemblies to dissipate heat while maintaining electrical isolation. These liquid dispensed silicone gap fillers offer performance advantages over pre-cured gap pads primarily due to their soft and conformable nature as well as being formed in place, conforming to the lid or other electronic component and the heat sink.

Momentive Performance Materials develops and commercially manufactures silicone liquid dispensed gap fillers which have bulk thermal conductivity values up to 10 W/mK with materials of greater thermal conductivity in the research phase. These materials, including TIA282GF, 1528-1-53, and XE14-C7412 are two-part silicone systems, which after being mixed at a 1:1 by mass ratio, cure to soft and conformable highly thermally conductive and electrically isolating silicones.

Analysis performed on TIA282GF (nominal 8 W/mK thermal conductivity), 1528-1-53 (nominal 9 W/mK thermal conductivity), and XE14-C7412 (nominal 10 W/mk thermal conductivity) included thermal conductivity along with many manufacturing related properties. Work life of these materials was evaluated by measuring the viscosity of catalyzed material at numerous time intervals post catalyzation. Dispense rate from various dispense tip diameters was also investigated on the multiple materials at various time intervals after catalyzation. An additional evaluation on TIA282GF, 1528-1-53, and XE14-C7412 that was performed was bond line thickness from various application pressures for various time intervals after catalyzation and dispensing.

The analyzed performance of TIA282GF, 1528-1-53, and XE14-C7412 in the work life, dispense rate, and resulting bond line thickness evaluations is critical to electrical assembly manufacturing process development.

## 15. Session: Posters

### 15.1. Application and Development on Multi-Samples Thermal Analysis in Tobacco Pyrolysis and Combustion Process

**Bin Li** **^1^** ^,^*, Lili Fu **^1^, Yue Zhang** **^1^** **and Zhongya Guo** **^2^**
^1^  Zhengzhou Tobacco Research Institute of CNTC
^2^  China Tobacco Guangdong Industry Co., Ltd.
*  Correspondence: ztrilibin@163.com

**Abstract:** Tobacco is mainly consumed through combustion or pyrolysis, so the product characteristics and quality stability of tobacco products are closely related to their thermal properties. Traditionally, the weight loss and differential weight loss curves of powdered samples with a mass of no more than tens of milligrams were mainly collected through a thermogravimetric analysis to further analyze their thermal properties. However, tobacco products and their production processes involve a larger sample size, more diverse sample shapes and stacking states, and traditional microcalorimetric analysis clearly cannot meet the usage needs. Thus, a multi-sample tobacco heat treatment online analyzer with automatic sampling function has been developed. This instrument consists of an atmosphere generation module, a sample processing module, a weighing module, an automatic sampling module, and a data acquisition and control unit, etc. It can handle sample masses of several grams and autonomously adjust parameters such as carrier gas type, concentration, and flow rate. Moreover, it is capable of providing continuous sample injection detection under higher furnace temperature conditions. This instrument has been utilized for researching the consistency evaluation of tobacco formulas, the quality stability of cutting and drying processes, as well as the stability evaluation of cigarette manufacturing, based on thermal conversion properties. The development and application research of this instrument play an important role in guiding the stable production of tobacco products.

### 15.2. Application of Thermal Analysis to Evaluate Thermal State of Tobacco Materials in Heated Tobacco Products

Lili Fu *, Bin Li, Ke Zhang, Le Wang, Chunping Wang, Qi Zhang and Wenkui Zhu
Zhengzhou Tobacco Research Institute of CNTC
*  Correspondence: lili1101_fu@163.com

**Abstract:** Heated tobacco products (HTPs) play an important part of the next generation product (NGPs). Under the working condition of a HTP, a series of thermal physiochemical processes occur in tobacco materials that are dependent on the specific heating conditions. In order to establish a quantitative characterization of the thermal state of the tobacco materials irrespective of the heating method used by the HTP heating device (e.g., central vs. peripheral), this study constructed a universal multidimensional framework to evaluate the thermal state of all electrically heated tobacco products (e-HTPs). For this purpose, a combinational dataset corresponding to thermal state was defined, which involved 8 key thermophysical indices, such as the tobacco`s thermal mass loss, the release of nicotine, glycerol, carbon monoxide, oxygen consumption rate, and the releases of acetaldehyde and toluene as markers of volatile HPHCs. the framework of the thermal state could be used to quantitatively characterize the thermal state of e-HTPs irrespective of their heating mechanisms.

### 15.3. Tough and Recyclable Glass Fiber Composites with Tailored Dynamic Chemistry

**Menisha Koralalage, Anisur Rahman and Tomonori Saito**
Oak Ridge National Laboratory
*  Correspondence: mahappukorms@ornl.gov

**Abstract:** Glass fiber composites are paramount in the development of lightweight structural materials due to their exceptional strength and cost-effectiveness. Traditional glass fiber reinforced polymer (GFRP) composites, which rely on thermoset matrices, face significant challenges at the end of their lifecycle, including non-recyclable nature and fiber-polymer delamination caused by insufficient interfacial adhesion. This study addresses these issues by integrating vitrimer chemistry into GFRPs, utilizing a facile composite design with unmodified glass fiber surfaces. We present an effective approach to fabricating tough and closed-loop recyclable glass-fiber-reinforced vitrimer (GFRV) composites with exceptional interfacial adhesion. This is achieved by introducing a boronic ester-modified upcycled thermoplastic elastomer, a multi-diol crosslinker, and neat glass fibers (GFs). The dynamically crosslinked vitrimer resin interacts with the hydroxyl group-rich unmodified glass fiber surface, facilitating the formation of dynamic covalent bonds with boronic ester groups. This dynamic boronic ester exchange at the resin-fiber interface results in robust mechanical properties, strong fiber-matrix interfacial adhesion, thermal reprocessing, and efficient closed-loop recycling of both fiber and resin, addressing the challenge of end-of-life disposal of GFRP composites. The developed GFRV composites exhibit a remarkable 552% increase in in-plane shear toughness and a 27% increase in ultimate tensile strength compared to conventional epoxy-based matrices. Additionally, the GFRV composites demonstrate significantly lower embodied carbon values, representing a threefold decrease, and very low cumulative energy demand compared to commercial GFRP composites, ensuring a sustainable alternative. Furthermore, the upcycled polymer and the GFRV composites exhibit excellent adhesion to various surfaces, expanding the potential applications of the composite across diverse industries. This study underscores the effective utilization of vitrimer chemistry in addressing challenges associated with traditional GFRP materials, offering a promising approach toward sustainable and multifunctional structural materials.

### 15.4. Electrospinning of Non-Woven Fibrous Filtration Membranes from PVDF/Polyampholyte Blends

Anuja Jayasekara *, Peggy Cebe, Ayse Asatekin, Luca Mazzaferro and Ryan O’Hara
Tufts University
*  Correspondence: anuja.jayasekara@tufts.edu

**Abstract:** Poly (vinylidene fluoride) (PVDF) is widely used in filtration and waste-water treatment applications due to its excellent mechanical, chemical, and thermal stability [1]. However, its inherent high hydrophobicity leads to membrane fouling [2]. One way to increase fouling resistance is to improve the hydrophilicity of PVDF-based membranes through blending with more hydrophilic polymers [3]. Our research investigates the thermal and structural properties of blends of PVDF with polyampholytes, amphoteric macromolecules with positive and negative ionic groups showing excellent hydrophilicity [4]. Non-woven fibrous membranes were fabricated using electrospinning of PVDF and a random polyampholyte copolymer (r-PAC) blended at different concentrations. r-PAC is synthesized by randomly co-polymerizing three monomers that are either hydrophobic (2,2,2-trifluoroethyl methacrylate); positively charged ([2-(methacryloyloxy) ethyl]tri-methylammonium chloride); or negatively charged (methacrylic acid). The obtained fiber mats were characterized by thermogravimetric (TG) analysis, fast scanning calorimetry (FSC), and scanning electron microscopy (SEM). Thermogravimetry showed that the PVDF/r-PAC blends have a multi-step thermal degradation mechanism while PVDF homopolymer showed single-step thermal degradation. The glass transition temperature of r-PAC was observed around 165 °C in the FSC plots.

**References**

1. Sadeghi, I.; Govinna, N.; Cebe, P.; Asatekin, A. *ACS Appl. Polym. Mater*. **2019**, *1*, 765–776.
2. Wanke, D.; da Silva, A.; Costa, C. *Chem. Eng. Res. Des*. **2021**, *171*, 268–276.
3. Madaeni, S.; Zinadini, S.; Vatanpour, V. *J. Membr. Sci*. **2011**, *380*, 155–162.
4. Mazzaferro, L.; Lounder, S.J.; Asatekin, A. *ACS Appl. Mater. Interfaces* **2023**, *15*, 42557–42567.

### 15.5. Site Specific Thermomechanical Analysis of Large Format Printed Polymer Composite Structures

**Benjamin Blume** **^1^** ^,^*, Tyler Corum **^1^, Chad Duty** **^2^, Vipin Kumar** **^3^** **and Ahmed Hassen** **^3^**
^1^  University of Tennessee
^2^  University of Tennessee—Knoxville
^3^  Oak Ridge National Laboratory
*  Correspondence: bblume@vols.utk.edu

**Abstract:** Large-format additive manufacturing (LFAM) is advantageous for autoclave tooling applications as LFAM can print large (>1 m^3^), complex structures at high rates (~50 kg/h). Fiber reinforced polymers (FRPs) are typically used as feedstock for LFAM to increase stiffness and reduce coefficient of thermal expansion (CTE). However, with the addition of FRPs, LFAM structures exhibit an anisotropic response to thermal loading across a nonhomogeneous microstructure. Anisotropy is due to the orientation of fiber reinforcing materials that resist expansion in the longitudinal direction but provide little resistance in the transverse direction. The nonhomogeneous microstructure stems from variations in the fiber orientation across each bead due to shearing effects in the print nozzle. Fibers are shear aligned by the nozzle edge during extrusion resulting in variation of fiber orientation within the bead, with highly aligned regions at the bead edge and a more random orientation toward the center. This leads to varying thermomechanical properties across the bead as the orientation of each fiber dictates CTE.

This study measured the CTE of an LFAM structure made from 20% by weight carbon fiber reinforced acrylonitrile butadiene styrene (CF-ABS) using thermomechanical analysis (TMA). The LFAM structure was initially sampled from three different locations along the deposition path of the print in the form of a slice. Each slice was then further divided into 5 × 5 × 5 mm samples for TMA testing. The TMA samples were spaced across the span of a single bead to capture the effects of varying fiber orientation on CTE results from TMA testing. The sampling locations were left bead interface (LBI), left of center (LC), center (CB), right of center (RC), and right bead interface (RBI). The LBI and RBI locations both contained regions of highly aligned fibers in deposition direction. The LC and RC samples contain a portion of highly aligned regions, but most of the sample is composed of randomly orientated fibers. CB samples primarily contain randomly oriented fibers. The CTE values for each sample were averaged between the different slice locations of the structure to represent a homogenized thermal response that accounts for the complex LFAM microstructure. This data is useful input for a finite element model that can predict the thermal expansion of LFAM structures for applications like printed composite tooling.

### 15.6. Fast Scanning Calorimetry Study of Confinement Dynamics in Polyzwitterions Containing Molar Ratios of Salt

John Thomas *, Sophia Dinn, Ashleigh Herrera, Ayse Asatekin and Peggy Cebe
Tufts University
*  Correspondence: john.thomas@tufts.edu

**Abstract:** The confinement seen in crosslinked polyzwitterions (PZIs) is analogous to the confinement seen in semi-crystalline polymers. As confinement in a semi-crystalline polymer increases, the breadth of transition increases, the glass transition temperature increases, and the heat capacity increment at Tg decreases. The crosslinking occurs by dipole-dipole interactions between differently charged moieties of the PZI side groups. In our study of the PZI poly(sulfobetaine acrylate), PSBA, complexes of PZI-salt were created by blending the following salts: LiCl, NH_4_Cl, and Na_2_SO_4_, at a 1:1 molar ratio of PZI monomeric unit to moles of the salt anionic unit. Through the dissolution of the salt into PSBA, disruptions of the dipolar crosslinking occur due to the association of anions and cations ions onto the zwitterionic side groups. In the presence of salt ions, the glass transition temperature decreased while the heat capacity increment at Tg increased showing that these salts act as a plasticizing agent. PZIs have high glass transition temperatures relative to conventional polymers/ionic glass formers and have low fragility indices. Using fast scanning calorimetry to avoid degradation, the fragility indices for PSBA-salt complexes was studied as a function of LiCl, NH_4_Cl, and Na_2_SO_4_ content, and compared to the homopolymer PZI without salt addition.

### 15.7. Interlayer Bonding in Hybrid Material Fused Filament Fabrication Processes

**Micah Vranes** **^1^** ^,^* **and Philip Barnett** **^2^**
^1^  University of Tennessee—Knoxville
^2^  University of Tennessee
*  Correspondence: mvranes@vols.utk.edu

**Abstract:** The fused filament fabrication process is one of the most accessible additive manufacturing (AM) processes available to a wide user base. In this process, layers of polymer are deposited layer by layer to form complex structures. In general, the strength has been found to be anisotropic, where the build direction is significantly weaker than in the build plane. This is primarily due to imperfect interlayer bonding, the strength of which is a function of variables such as temperature, layer height, and print velocity. Much of the bonding is diffusion limited, where cooling between subsequent layers limits polymer diffusion across the interface, thereby weakening the printed part. The recent advent of multi-material printers has created unique opportunities for the creation of functionally graded structures, but the interlayer bonding of multi-material prints remains mostly unexplored. In this work, we explored the interlayer bond development in multi-material 3D printed parts of polyethylene terephthalate glycol (PETG) and PETG/carbon fiber, representing sandwich structures used to produce high strength, low density parts. We studied the interlayer bond strength as a function of print speed through Mode I fracture toughness measurements to provide insight into the interlayer bond development to better understand optimal print parameters required for producing high quality AM parts.

### 15.8. Nylon 6 Deconstruction and Upcycling: From Waste to a New Place

**Jackie Zheng** **^1^** ^,^*, Mairead Boucher **^2^, Nick Galan** **^2^, Jeff Foster** **^2^,** **Tomonori Saito** **^2^** **and Md Arifuzzaman** **^3^**
^1^  University of Tennessee—Knoxville
^2^  Oak Ridge National Laboratory
^3^  Re-Du
*  Correspondence: jzheng17@vols.utk.edu

**Abstract:** Nylon is a high-performing, versatile polymer due to its high strength and durability. Over 8.7 million tons of nylon are produced annually, and their application in textiles as fibers makes it the second highest produced synthetic fiber. Other applications include automotive parts, engineering plastics, and electronics, bringing the global nylon market size to be 32.6B USD in 2022. Most of the commercially produced nylons are synthesized from fossil fuel sources due to their inexpensive cost and established manufacturing process. However, end-of-life nylon products are hard to recycle in a cost-effective and energy efficient way. Current nylon recycling processes include grinding nylon waste to produce downcycled nylons which are used as low-end filler materials. Thus, establishing chemical deconstruction of nylon in an energy-efficient and cost-effective manner to produce building blocks for subsequent upcycling is an important strategy for nylon waste management.

In this study, nylon-6 (Mw = 40 kg/mol) is deconstructed via an organocatalyst catalyzed glycolysis process to produce shorter chain and oligomers of nylon-6. By changing the stoichiometric ratios of ethylene glycol and reaction times, the glycolysis process can be tailored to achieve deconstructed products of specific molecular weights. Our strategy of deconstructing nylon-6 into shorter chain polymers rather than converting it to monomers conserves energy and resources in repolymerizing the material into new polymers. Furthermore, the deconstructed nylon 6 products of tailored molecular weights are copolymerized with other building blocks to produce upcycled polymers having new properties and improved circularity. Nuclear magnetic resonance spectroscopy and size exclusion chromatography are used to verify tailored deconstruction as well as new copolymers synthesized from the shorter chain nylon-6 deconstruction products. Thermal stability and the change of glass transition and melting temperature are characterized via thermal gravimetric analysis and differential scanning calorimetry. Mechanical properties are characterized by tensile measurements and dynamic mechanical analysis.

### 15.9. Elucidating the Role of Ionic Liquids on the Deconstruction of Step-Growth Polymers

**Nick Galan** **^1^** ^,^*, Christine Rukeyser **^1^, Subhamay Pramanik** **^1^, Bobby Sumpter** **^1^, Jeff Foster** **^1^, Tomonori Saito** **^1^** **and Jackie Zheng** **^2^**
^1^  Oak Ridge National Laboratory
^2^  University of Tennessee—Knoxville
*  Correspondence: galannj@ornl.gov

**Abstract:** Step-growth polymers such as poly(ethylene terephthalate) (PET), polyamide (PA), polyurethane (PU), and polycarbonate (PC) comprise ~30% of the global plastic production. However, accessing targeted product distributions (i.e., oligomers, dimers, monomers, etc.) remains a significant challenge. A variety of Ionic liquids (ILs) have been shown to promote the deconstruction of various step growth polymers, however, it is not well-understood how their cation/anion relationship influence product distribution. In this work, we employed a guided DFT approach to elucidate how the PET glycolysis was mediated by the dissociation energy and ethylene glycol activation energy of imidazolium and pyrrolidinium based ILs, with electronically distinct counter anions. When ILs have anions of strong acids they exhibit high thermal stability, but PET deconstruction is limited to lower molecular weight (MW) (15^−8^ kDa) products. On the other hand, ILs bearing anions derived from weak acids promoted near quantitative PET conversion, but afforded varied amounts of monomer, dimer, and trimer products as determined by HPLC analysis. These yields can be tuned by increasing the loading of IL. Furthermore, we have demonstrated that ILs with acetate anions promote productive deconstruction of other step-growth polymers such as PC and PA. These results shed light on how anion electronics/basicity can be tuned to influence the product distribution from lower MW oligomers toward monomeric, dimeric, and trimeric species of PET.

### 15.10. 3D-Printing Formulated Polyelectrolyte Complexes (PECs) in Air via Direct Ink Writing

**Anh Nguyen** **^1^** ^,^*, Alicja Jurago **^1^, Rigoberto Advincula** **^2^** **and Charles Patten** **^2^**
^1^  University of Tennessee
^2^  University of Tennessee—Knoxville
*  Correspondence: anguye22@vols.utk.edu

**Abstract:** Thermal Polyelectrolyte complexes (PECs) are versatile materials with a wide range of applications in the biomedical and energy industries, including membranes, coatings, films, fibers, and pharmaceutical products. The ionic bonds within PECs can be disrupted by dissolving them in salt, which plasticizes the material and enhances its mobility, making it ideal for 3D printing using direct ink writing (DIW). This study introduces novel 3D printing techniques for strong polyelectrolytes, specifically poly(styrenesulfonate) (PSS) and poly(diallyldimethylammonium) (PDADMAC), dissolved in aqueous potassium bromide (KBr).Two innovative approaches were developed: one utilizing silica nanoparticles as a rheological modifier, and the other involving the deposition of viscous PEC solutions layer-by-layer, followed by quenching with deionized water to form mechanically robust, viscoelastic hydrogels with intricate geometries. These techniques optimize the printing process, ensuring high-resolution prints and mechanically viable structures, expanding the potential applications of PECs in advanced manufacturing.

### 15.11. Enhancing Anti-Corrosion Properties of PANI- via Electrodeposited Composite Films with GO and PVK Additives

Zachary Watson *, Evelyn Winn, Karen Yanith Patiño Jaimes, Rigoberto Advincula and Ellis Kim
University of Tennessee—Knoxville
*  Correspondence: zactwats@vols.utk.edu

**Abstract:** Conductive polymers have gained significant popularity for their diverse range of applications, including coatings [1], capacitors [2], and sensors [3]. Electrochemical deposition enables precise control over thickness, coating uniformity, and adhesion, making it an ideal method for synthesizing conductive polymers on a conductive substrate [4]. Polyaniline (PANI) is a commonly used conductive polymer obtained through the oxidation reaction of aniline monomer. PANI exhibits desirable properties such as environmental stability, non-toxicity, and ease of modification [4]. Depending on the degree of oxidation, PANI can exist in the form of Emeraldine Salt (PANI-ES), which possesses high conductivity and excellent protection characteristics.

This study aims to demonstrate the enhancement of PANI-ES properties through the incorporation of additives such as graphene oxide (GO) and polyvinyl carbazole (PVK). GO serves as a filler, offering chemical inertness, barrier properties, and robustness [5], while PVK aids in the dispersion of GO [6]. By employing electrodeposition techniques onto an Indium Titanium Oxide substrate, a thin conductive film with anti-corrosion properties was successfully produced. To evaluate the anti-corrosion properties, various electrochemical measurements, including Electrochemical Impedance Spectroscopy (EIS) and Potentiodynamic Polarization, were conducted. The sample with a GO:PVK mass ratio of 1:2 exhibited the highest resistance, 9.91E4 Ohms cm^2^. Additionally, chemical and surface characterization techniques such as UV-VIS, Raman Spectroscopy, AFM, contact angle, and SEM were employed. Noteworthy results from these characterizations include the composite with the highest contact angle observed at a GO: PVK mass ratio of 1:1 (105°), and the average thickness of all samples was determined to be 2.5 +/− 5 µm. Anti-microbial properties will be also tested by evaluating the bacterial growth inhibition properties of the film [7]. These findings contribute to the development of thin, conductive films with improved protective properties, demonstrating the potential of these composites in various industrial and technological applications.

**References**

1. Caldona, E.B.; de Leon AC, C.; Pajarito, B.B.; Advincula, R.C. Novel Anti-Corrosion Coatings from Rubber-Modified Polybenzoxazine-Based Polyaniline Composites. *Appl. Surf. Sci*. **2017**, *422*, 162–171. https://doi.org/10.1016/j.apsusc.2017.05.083.
2. Wang, H.; Hao, Q.; Yang, X.; Lu, L.; Wang, X. Graphene Oxide Doped Polyaniline for Supercapacitors. *Electrochem. Commun*. **2009**, *11*, 1158–1161. https://doi.org/10.1016/j.elecom.2009.03.036.
3. Park, S.; Gordon, C.T.; Swager, T.M. Resistivity Detection of Perfluoroalkyl Substances with Fluorous Polyaniline in an Electrical Lateral Flow Sensor. *Proc. Natl. Acad. Sci. USA* **2024**, *121*. https://doi.org/10.1073/pnas.2317300121.
4. Mohd, Y.; Ibrahim, R.; Zainal, M.F. Electrodeposition and Characterization of Polyaniline Films. In *SHUSER 2012–2012 IEEE Symposium on Humanities, Science and Engineering Research*; IEEE: Piscataway, NJ, USA, 2012; pp. 1301–1306. https://doi.org/10.1109/SHUSER.2012.6268811.
5. Ding, R.; Li, W.; Wang, X.; Gui, T.; Li, B.; Han, P.; Tian, H.; Liu, A.; Wang, X.; Liu, X.; Gao, X.; Wang, W.; Song, L. A Brief Review of Corrosion Protective Films and Coatings Based on Graphene and Graphene Oxide. *J. Alloys. Compd*. **2018**, *764*, 1039–1055. https://doi.org/10.1016/J.JALLCOM.2018.06.133.
6. Edaugal, J.P.; Ribeiro, E.L.; Mitchell, M.K.; Cheng, X.; Buckner, E.M.; Chen, J.; Ivanov, I.N.; Ellermann, M.; Advincula, R.C. Digital Light Processing (DLP): 3D Printing of Polymer-Based Graphene Oxide Nanocomposites—Efficient Antimicrobial Material for Biomedical Devices. *MRS Commun*. **2023**, *13*, 594–602. https://doi.org/10.1557/S43579-023-00390-X.
7. Santos, C.M.; Tria MC, R.; Vergara RA, M.V.; Ahmed, F.; Advincula, R.C.; Rodrigues, D.F. Antimicrobial Graphene Polymer (PVK-GO) Nanocomposite Films. *Chem. Commun.* **2011**, *47*, 8892–8894. https://doi.org/10.1039/C1CC11877C.

### 15.12. Thermal, Mechanical, and Unusual Crystallization Properties of Sustainable Aliphatic Polyesters Derived from 1,18 Octadecanedioic Acid and α-ω Diol Congeners from 2 to 18 CH2

Rufina G. Alamo *, Nickolas Boyd, Clay Kramer and Hamed Janani
FAMU-FSU College of Engineering
*  Correspondence: alamo@eng.famu.fsu.edu

**Abstract:** Thermal Ester groups interspersed in a predominantly polyethylene backbone act as cleavable points, representing a strategy to achieve deconstructable polyethylene mimics. Long-spaced aliphatic polyesters are possible substitutes for commodity polymers derived from fossil fuel feedstocks. The thermal, crystalline structure, and crystallization kinetics of a series of aliphatic polyesters type PE-X,18 (with 2 3 is orthorhombic, while polyesters with the shortest diol (X = 2, 3) develop hexagonal packing when fast-quenched from the melt, and orthorhombic under slower crystallization. The isothermal crystallization rates are unusual, exhibiting local rate minima at temperatures where the evolution of crystallinity is very slow. Hence, the rate minima are “kinetic traps” of relevance in processing these materials from the melt.

### 15.13. Thermomechanical Properties of Sequence-Defined Polyamides

Isaiah Dishner * and Jeff Foster
Oak Ridge National Laboratory
*  Correspondence: dishnerit@ornl.gov

**Abstract:** The primary structure of macromolecules profoundly impacts their thermomechanical performance. Although it is generally understood that altering monomer sequence can substantially impact bulk properties such as (semi)crystallinity and hydrophilicity, sequence-property relationships remain underexplored. In this work, we use iterative synthesis to build sequenced diad, triad, and tetrad oligoamides from a variety of diacid and diamine building blocks. These sequenced oligomers are subsequently polymerized through conventional solid-state polymerization to compare their performance against non-sequenced analogues. Copolymer sequence fidelity is confirmed by nuclear magnetic resonance spectroscopy. Molecular weight as determined by size exclusion chromatography is found to be comparable to polyamides synthesized from traditional diacid/diamine salts. Finally, we compare thermomechanical performance of sequenced copolyamides to their random counterparts using differential scanning calorimetry, dynamic mechanical analysis, tensile testing, and melt rheology. These results demonstrate the importance of monomer sequence in determining material performance. Future studies will seek to produce sequenced polymers more efficiently, and to explore additional chemistries and functionalization pathways to introduce new functionality.

### 15.14. Application of Data Loggers, Heat Balance and Kinetic Analysis of HFC, DSC or ARC Data for Evaluation of Safety Parameters of Energetic Materials. Remote Monitoring of the Remaining Shelf-Life

**Bertrand Roduit** **^1^** ^,^*, Damiano Rickenbach **^1^, Patrick Folly** **^2^, Alexandre Sarbach** **^2^** **and Richard Baltensperger** **^3^**
^1^  AKTS SA
^2^  armasuisse
^3^  University of Applied Sciences of Western Switzerland
*  Correspondence: b.roduit@akts.com

**Abstract:** Most goods made by human hands are subject to decay, whose rates determine their shelf life (SL). In general terms, SL can be defined as a finite period after production during which the product retains a required level of quality under well-defined storage conditions.

To be considered applicable and/or safe, the products

(i) must fulfil during the SL the properties set by the producers
(ii) their Self-Accelerating Decomposition Temperature (SADT) must meet the regulations set by the United Nations (UN) for the storage and transport of dangerous goods.

The SL of a product is directly related to the rate of its deterioration, which depends on external parameters, among which the temperature plays the most crucial role. Predicting when the acceptability limit of quality is reached requires knowledge of the kinetics of deterioration. This issue becomes even more critical in the last leg of the supply chain when the temperature fluctuates during storage or transport. Therefore, the proper evaluation of the current state of the ageing degree of the materials requires advanced kinetic tools, which additionally can continuously predict the rate of material deterioration at any time, temperature profile and localization during transport. In the present study, we propose a solution which applies an online system that receives the temperature (T) data supplied by T-data-loggers using Internet Of Things (IoT) capabilities. By combining onboard GPS for continuous geo-location, a “Shelf Life Monitoring System” has been developed to provide a global solution for remote, 24/7 tracing, monitoring and predicting the degree of material degradation and thermal safety in any location and any environment. Due to the continuous monitoring of the reaction progress, the kinetic parameters allow calculating the remaining shelf life of the materials at any storage or transport conditions. The potential of the proposed approach is wide-ranging, including the pharmaceutical (vaccines, drugs), food and chemical industries and the safety for the surveillance of dangerous goods. The cost of implementing the Internet Of Things (IoT) capabilities during transport and storage will be covered by the savings caused by reducing product waste and preventing accidents.

**References**

1. AKTS-Thermokinetics Software (V 6.02), AKTS SA, Technopôle 1, Sierre, Switzerland. Available online: https://www.akts.com (accessed on 17 May 2024).
2. Roduit, B.; Luyet, C.; Hartmann, M.; Folly, P.; Sarbach, A.; Dejeaifve, A.; Dobson, R.; Schroeter, N.; Vorlet, O.; Dabros, M.; Baltensperger, R. *Molecules* **2019**, *24*, 2217.
3. Evers, A.; Clenet, D.; Pfeiffer-Marek, S. *Pharmaceutics* **2022**, *14*, 375.
4. Huelsmeyer, M.; Kuzman, D.; Martinez, J.; Steinbrugger, C.; Weusten, J.; Calero-Rubio, C.; Roche, W.; Niederhaus, B.; VanHaelst, Y.; Hrynyk, M.; Ballesta, P.; Achard, H.; Augusto, S.; Guillois, M.; Pszczolinski, C.; Gerasimov, M.; Neyra, C.; Ponduri, D.; Ramesh, S., Cleacutenet, D. Scientific Reports. *Nature*. **2023**, *13*, 10077.

### 15.15. Eco-Friendly Water-Based Conducting Ink Using Cellulose and Polyaniline for Printable Electronics

**Karen Yanith Patiño Jaimes** **^1^** ^,^*, Alexandra Perez **^1^, Rigoberto Advincula** **^1^** **and Karl Albright Tiston** **^2^**
^1^  University of Tennessee—Knoxville
^2^  Chulalongkorn University
*  Correspondence: kpatino@vols.utk.edu

**Abstract:** Conductive polymers have gained significant attention for their diverse range of applications, including coatings [1], sensors[2], supercapacitors [3], wearable devices [4], and electronics [5,6]. In particular, some of these polymers have been engineered to create inks for printable electronics [3,4,7]. Despite having substantial flexibility, electrical conductivity, and processability, the eventual dehydration may diminish these desired material properties [8]. Thus, alternative polymer materials for the conductive inks in electronics remain open for exploration.

This study aims to develop an eco-friendly, water-based polyaniline (PANI) ink for electronic printing using a commercial desktop printed circuit board (PCB) printer. PANI is a conductive polymer with environmental stability, non-toxicity, and ease of modification [9]. Using the most conductive emeraldine salt form (PANI-ES), an extrudable conductive ink is compounded by mixing PANI with carboxymethyl cellulose (CMC) as viscosity modifier [10], and glycerol as plasticizer [11]. Graphene is also added to increase conductivity and adhesion [12]. Electrical conductivity of the resulting film will be measured using a four-point probe method, and its mechanical properties will be evaluated using a universal testing machine. Finally, the ink functionality will be demonstrated by printing a circuit using the Voltera™ printer. This work aims to provide a simple approach in the preparation of renewable and green conductive inks for future devices such as flexible sensors, soft robotics, and biocompatible human-machine interfaces.

**References**

1. Caldona, E.B.; de Leon AC, C.; Pajarito, B.B.; Advincula, R.C. Novel Anti-Corrosion Coatings from Rubber-Modified Polybenzoxazine-Based Polyaniline Composites. *Appl. Surf. Sci*. **2017**, *422*, 162–171. https://doi.org/10.1016/j.apsusc.2017.05.083.
2. Park, S.; Gordon, C.T.; Swager, T.M. Resistivity Detection of Perfluoroalkyl Substances with Fluorous Polyaniline in an Electrical Lateral Flow Sensor. *Proc. Natl. Acad. Sci. USA* **2024**, *121*, e2317300121. https://doi.org/10.1073/pnas.2317300121.
3. Tadesse, M.G.; Simon, N.; Felix Lübben, J. Highly Conductive Films through PEDOT-PSS Ink Formulation via Doping Using Spontaneous Wicking of Liquids for Supercapacitor Applications. *Adv. Energy Sustain. Res.* **2024**, 2400006. https://doi.org/10.1002/AESR.202400006.
4. Galliani, M.; Ferrari, L.M.; Bouet, G.; Eglin, D.; Ismailova, E. Tailoring Inkjet-Printed PEDOT:PSS Composition toward Green, Wearable Device Fabrication. *APL Bioeng* **2023**, *7*, 16101. https://doi.org/10.1063/5.0117278.
5. Kwiatkowska, E.; Mech, W.; Wincukiewicz, A.; Korona, K.P.; Zarębska, K.; Kamińska, M.; Skompska, M. Investigation of Polyaniline Doped with Camphorsulfonic Acid in Chloroform Solution as a Hole Transporting Layer in PTB7: PCBM and Perovskite-Based Solar Cells. *Electrochim. Acta* **2021**, *380*, 138264. https://doi.org/10.1016/J.ELECTACTA.2021.138264
6. Dongmin Kang, S.; Jeffrey Snyder, G. Charge-Transport Model for Conducting Polymers. *Nat. Mater.* **2016**, *16*, 2521257. https://doi.org/10.1038/nmat4784.
7. Fan, X.; Nie, W.; Tsai, H.; Wang, N.; Huang, H.; Cheng, Y.; Wen, R.; Ma, L.; Yan, F.; Xia, Y. PEDOT:PSS for Flexible and Stretchable Electronics: Modifications, Strategies, and Applications. *Adv. Sci.* **2019**, *6*, 1900813. https://doi.org/10.1002/ADVS.201900813.
8. Ren, T.; Yang, H.; Zhang, J.; Lv, K.; Kong, D.; Jiang, F.; Chang, Y.; Yu, P.; Tao, J.; Wang, D.; Kong, N.; Shao, Y. PEDOT:PSS/Natural Rubber Latex Inks for Screen-Printing Multifunctional Wearable Strain—Humidity Sensors. *Adv. Eng. Mater*. **2023**, *25*. https://doi.org/10.1002/ADEM.202301018.
9. Mohd, Y.; Ibrahim, R.; Zainal, M.F. Electrodeposition and Characterization of Polyaniline Films. In *SHUSER 2012–2012 IEEE Symposium on Humanities, Science and Engineering Research*; IEEE: Piscataway, NJ, USA, 2012; pp. 1301–1306. https://doi.org/10.1109/SHUSER.2012.6268811.
10. Li, Y.; Gong, Q.; Han, L.; Liu, X.; Yang, Y.; Chen, C.; Qian, C.; Han, Q. Carboxymethyl Cellulose Assisted Polyaniline in Conductive Hydrogels for High-Performance Self-Powered Strain Sensors. *Carbohydr. Polym*. **2022**, *298*, 120060. https://doi.org/10.1016/J.CARBPOL.2022.120060.
11. Tarique, J.; Sapuan, S.M.; Khalina, A. Effect of Glycerol Plasticizer Loading on the Physical, Mechanical, Thermal, and Barrier Properties of Arrowroot (Maranta Arundinacea) Starch Biopolymers. *Sci. Rep.* **2021**, *11*, 1–17. https://doi.org/10.1038/s41598-021-93094-y.
12. Wang, H.; Hao, Q.; Yang, X.; Lu, L.; Wang, X. Graphene Oxide Doped Polyaniline for Supercapacitors. *Electrochem. Commun*. **2009**, *11*, 1158–1161. https://doi.org/10.1016/j.elecom.2009.03.036.

### 15.16. Crystallization and Rigid Fraction of PLLA

Logan Williams * and Sindee Simon
North Carolina State University
*  Correspondence: lwilli25@ncsu.edu

**Abstract:** Fast scanning calorimetry (Flash DSC) is used to study the crystallization kinetics for a model polymer, poly(L-lactic acid) (PLLA) and the concomitant development ofthe so-called rigid amorphous phase or RAF, which we refer to as simply as the rigid fraction (RF) given the debate over its nature. Contrary to recent studies, a rigid fraction of 15–20% forms during crystallization for both a’ and a polymorphs of PLLA. Kinetic modeling using the Avrami model is used to study the development of both the crystallization and the formation of the RF.

### 15.17. Color Additive Effects on the Rheological Properties of Recycled LFAM Materials

**Ally Collier** **^1^** ^,^*, Roo Walker **^1^, Nina Bhat** **^1^, Chad Duty** **^1^, Dylan Hoskins** **^2^** **and Kyle Rowe** **^2^**
^1^  University of Tennessee—Knoxville
^2^  Haddy
*  Correspondence: acolli70@vols.utk.edu

**Abstract:** With the increasing demand for recycled materials and sustainable manufacturing processes, large format additive manufacturing (LFAM) has emerged as a manufacturing technique capable of utilizing mechanically recycled short fiber thermoplastic composites (SFRPs) for a variety of applications [1,2]. An up-and-coming application space that is enabled by LFAM is the design and development of urban furniture using recycled SFRP materials. Urban furniture plays a key role in the development of sustainable urban spaces, where sustainable materials and design intersect with the needs for sustainable community development. When utilizing recycled materials in LFAM, it is important to consider how processing conditions may change based on the degradation introduced by the recycling process; previous research has shown that matrix and fiber phase degradation are correlated to changes in processing behavior like viscosity during deposition. However, for applications like urban furniture where colorants may be used in the feedstock, we must also consider how the addition of colorants may also influence the materials processing behavior after recycling. Therefore, this study assessed the role that color additives have on the rheological properties of three differently colored post-industrial recycled glass-fiber polyethylene-terephthalate-glycol (PIPG) LFAM feedstock materials.

To better understand how processing conditions may change because of the color of the PIPG, the complex viscosity of black, dark grey, and white PIPG were observed using parallel plate frequency sweeps from 0.1–628 rad/s at the materials’ deposition temperature. The complex viscosity at 100 rad/s for each material was observed and compared to determine differences in rheological behavior. The frequency of interest was selected to best represent the shear rate region that a material may experience during LFAM deposition. It was found that the black material had the highest complex viscosity (945 Pa-s) whereas the white and grey PIPG had a 43% and 24% lower complex viscosity, respectively. In addition to the difference in complex viscosities at 100 rad/s, there were notable differences in the shear thinning behavior of the white and dark grey materials compared to the black. The power law index (n) of the black, white, and dark grey PIPG was calculated and were found to be 0.6, 0.7, and 0.8, respectively. The differences in n indicate a difference in shear thinning behavior, where the black PIPG experiences more shear thinning than the white and dark grey PIPG materials.

As the feedstock materials are made from the same base PIPG material it can be proposed that the change in rheological properties is a function of the pigment used to color the PIPG materials. Carbon black (CB) and titanium dioxide (TiO_2_) are commonly used pigments to achieve black and white colored materials, respectively. Previous research has shown that materials reinforced with CB, despite smaller particle size, have a higher viscosity than materials reinforced with TiO_2_ and is correlated to the particle-particle interaction differences between CB/TiO_2_ and the polymer matrix [3]. The phenomena observed in the grey material is believed to be due to particle size heterogeneity due to the mixing of pigments and its effect on both interparticle interactions and hydrodynamic forces experienced by the material. The investigation of the effects of colorants within a recycled material can make determining processing conditions easier and allow for adjustments to be made prior to manufacturing. This study shows that colorants do influence processing behavior, like viscosity, at deposition and therefore should be considered when utilizing colored recycled materials in LFAM applications.

**References**

1. Duty, C.E.; Love, L.J. *Cincinnati Big Area Additive Manufacturing (BAAM)*; Oak Ridge National Lab. (ORNL): Oak Ridge, TN, USA, 2015. https://doi.org/10.2172/1210140.
2. Walker, R.; Korey, M.; Hubbard, A.M.; Clarkson, C.M.; Corum, T.; Smith, T.; Duty, C. Recycling of CF-ABS machining waste for large format additive manufacturing. *Compos. Part B Eng.* **2024**, *275*, 111291. https://doi.org/10.1016/j.compositesb.2024.111291.
3. Tanaka, H.; White, J.L. Experimental investigations of shear and elongational flow properties of polystyrene melts reinforced with calcium carbonate, titanium dioxide, and carbon black. *Polym. Eng. Sci*. **1980**, *20*, 949–956. https://doi.org/10.1002/pen.760201406.

### 15.18. The Glass Transition and TTT Diagram in Indomethacin/Sucrose Benzoate Co-Amorphous Drug Delivery Systems

Elaheh A. T. Moghadam * and Sindee Simon
North Carolina State University
*  Correspondence: eabdoll@ncsu.edu

**Abstract:** The glass transition temperature (Tg) and related dynamics, as well as isothermal crystallization, are investigated for binary miscible mixtures of indomethacin and sucrose benzoate using differential scanning calorimetry to study the stability of the systems. These mixtures are termed co-amorphous glasses, and such materials have been shown to have enhanced water solubility and bioavailability relative to the crystalline forms of active pharmaceutical ingredients. For the co-amorphous glasses studied, the composition-dependent Tg displays negative deviations from expectations for an athermal mixture, and the data cannot be described by the Fox equation. The ability of other approaches to describe composition-dependent Tg will be discussed. In addition, the cooling rate dependence of Tg and the fragility of each mixture is determined in an effort to clarify the relationship between fragility, ease of glass formation, and stability of the glass against crystallization. The crystallization data for indomethacin as a function of temperature and time are modeled using the Avrami model. These data are also used to obtain the time-temperature-transformation (TTT) diagram, which is modeled using nucleation rate theory. In addition, the crystallization of indomethacin/sucrose benzoate co-amorphous materials is studied at 100 °C and fit to the Avrami model. The preliminary results show that co-amorphs having less than 70% indomethacin are more stable and take longer to crystallize than the pure material.

### 15.19. Thermophysical Properties of p-Quaterphenyl

Andrew McGhie * and Steven J. Szewczyk
University of Pennsylvania
*  Correspondence: mcghie@lrsm.upenn.edu

**Abstract:** The high temperature properties of ultrapure p-quaterphenyl have been studied by DSC, at heating rates ranging from 0.01 °C/min. to 10 °C/min, to measure the true melting point (onset of melting) and heat of fusion using single crystalline material grown by the Bridgman technique from zone refined starting material, scintillation grade p-quaterphenyl. The melting point onset of high purity p-quaterphenyl was determined to be 314.8 °C +/− 0.2 °C. and the Heat of Fusion, ∆Hf = 49. 7 +/− 0.3 KJ/mol., using high purity Pb, mp 327.5 °C as calibrant. These numbers disagree with reported literature values. In addition, evidence for a high temperature crystalline transition, ∆Htrans~0.6–1.2 KJ/mol, was observed in the range 150–250 °C in agreement with the findings of Wasiki & Radomska. Evidence for a nematic phase was observed within the 0.3 °C melting range observed on scanning at 0.01 °C/min. Interestingly, the temperature decreased slightly during the nematic-isotropic liquid transition and the reason will be discussed. The system was checked using high purity single crystalline p-terphenyl whose melting point was determined to be 212.7 °C and ∆Hf = 35.4 kJ/mol., in agreement with Wasiki & Radomska, using pure Sn, mp 231.9 °C, as calibrant.

### 15.20. Polymers as Electrolytes for Lithium Batteries

Isaac Sutton *
University of Tennessee—Knoxville
*  Correspondence: Sutton.ij.08@gmail.com

**Abstract:** Lithium Ion Batteries (LIBs) and Lithium Metal Batteries (LMBs) are crucial components of modern society’s energy storage needs. Electrolytes are one of the three main components of these batteries, with liquid electrolytes being the most widely used today mainly due to their high conductivity. However, their use in energy-demanding applications is restricted by their toxicity, flammability, and reactivity.

Solid polymer electrolytes (SPEs), and especially Single-Ion Solid Polymer Electrolytes (SLIC-SPEs), are a promising alternative due to their sufficient thermal and mechanical stability, non-toxicity, flexibility, and easy processability. The main limitation is their low conductivity and the sacrifice of good mechanical properties to enhance it.

SLIC-SPEs with complex architecture (star polymers) using monomeric lithium salts (MASTFSILi) for the electrolyte preparation and making blends of them with low Molecular Weight (MW) Poly Ethylene Oxide (PEO) is our answer to the issue. The polymeric star salts have a dual role as will be the source of the Li cations while providing good mechanical properties due to their stiff core. The PEO due to its low MW and low Glass Transition Temperature (Tg) will be the conductive face for the Li cations. We expect that the architecture will compromise the two competitive properties and allow their deconvolution.

### 15.21. Characterization of Evolving Semi-Crystalline Carbon Structures from Bis-Ortho-Diynylarene (BODA) Homo- and Co-Polymer Networks

**William Johnson** **^1^** ^,^*, Patrick Madden **^1^, Joshua Brown** **^1^, Charles Pittman** **^1^, Dennis Smith** **^1^** **and Ernesto Borrego** **^2^**
^1^  Mississippi State University
^2^  Hand Technologies, LLC
*  Correspondence: wwj38@msstate.edu

**Abstract:** Bis-ortho-diynylarene (BODA)-derived resins (BDR) are a class of melt-processable high yielding (>80%) carbon precursor resins that form highly crosslinked polyarylene networks via a thermally initiated Bergman cyclization. The thermoset networks therefrom have low flammability, high thermal oxidative stability, and convert efficiently into carbon. Due to these advantageous properties, this material system can be utilized to rapidly produce high value carbon/carbon composite structures for ultra-high temperature aerospace applications. Thermal conversion, carbonization kinetics, and thermal degradation studies were conducted on select BDR homo- and co-formulations to further understand the conversion of BDR into carbon. This study sheds light on the chemical architecture and mechanisms of this system for achieving near quantitative carbon yields from polymeric carbon precursors.

### 15.22. Laser Synthesized High-Performance MnOx/rGO Hybrid Nanocomposite for Energy Storage Applications

Mahhsid Mokhtarnejad *, Soheil Almasi, Mariana Milano-Benitez and Bamin Khomami
University of Tennessee
*  Correspondence: mmokhtar@vols.utk.edu

**Abstract:** With the rising demand for efficient energy storage devices such as batteries and supercapacitors, there’s a growing focus on improving material performance through facile, reproducible, and cost-efficient synthesis methods. This means it is important to tune the shape, morphology, and overall functionality of these materials. This study explores the influence of the laser-solution-phase parameters on the synthesis of different MnOx phases (such as MnO nanoparticles, MnO_2_, and Mn_3_O_4_ nanorods) in Laser synthesized Manganese oxide composited with reduced graphene oxide (MnOx/rGO) and their performance as supercapacitor (SC) material. Our investigations indicate that an optimized concentration of MnO_2_ to MnO and Mn_3_O_4_ in the LASiS-synthesized MnOx/rGO hybrid nanocomposite (HNC) improves the capacitance properties of LASiS-derived HNCs. We will show their capacitance enhancement when used as high-performance supercapacitor material after introducing the optimal solution and laser parameters to optimize the MnO_2_ phase content in LASiS-derived HNCs. Additionally, we investigate the relationship between laser ablation parameters, solution temperature, and the resulting MnOx nanostructures. Our findings reveal that MnOx nanorods synthesized under eight minutes ablation time at a solution temperature of four degrees Celsius exhibit a capacitance of ~425 F/g at the current density of 2 A/g. These results show the efficacy of the specified conditions in optimizing the MnO_2_ phase, highlighting our observations of enhanced energy storage capabilities.

### 15.23. Controlling Interphase Morphology in Thermoplastic Carbon Fiber Composite Manufacturing

Kendra Allen ^1^^,^*, Logan Kearney ^2^, Amit Naskar ^2^, Jong Keum ^2^ and Hicham Ghossein ^3^
^1^  University of Tennessee—Knoxville
^2^  Oak Ridge National Laboratory
^3^  Endeavor Composites, Inc.
*  Correspondence: kallen58@vols.utk.edu

**Abstract:** Carbon fiber composites are an important category of materials in the energy industry that are being more widely used in the automotive, aerospace, and wind turbine sectors. Composite matrix materials are typically composed of thermoset materials (i.e., epoxies, polyurethanes, etc.) which adhere to the fiber surface utilizing specialized surface treatments and sizing techniques. In this work, a recently developed wet-lay technique was utilized to co-mingle chopped carbon fiber and isotactic polypropylene (iPP) fibers to form isotropic composites and optimize the interphase morphology performance by precisely controlling the thermal processing window. Thermal characterization and X-ray scattering were used to observe underlying microstructural attributes of composites under simulated processing conditions. Our results show that that carbon fiber increases nucleation rates, accelerates overall crystallization kinetics, expands the thermal envelope, and overall alters the morphology of the carbon fiber/iPP composites. These results provide a practical framework for the manufacturing of thermoplastic matrix composites.

### 15.24. Correlating Local Structural and Dielectric Heterogeneity with Charge Separation in Polymer Semiconductors

**Henry Kantrow** **^1^** ^,^*, Natalie Stingelin **^1^, Claire Welton** **^2^, Neethu Thomas** **^2^, Manjunatha Reddy** **^2^** **and Carlos Silva** **^3^**
^1^  Georgia Institute of Technology
^2^  Université de Lille
^3^  Université de Montréal
*  Correspondence: henryk@gatech.edu

**Abstract:** Polymer semiconductors have drawn increasing attention for energy-storage and -harvesting technologies due to their high tunability, mechanical robustness, and scalability compared to traditional inorganic semiconductors. These emerging photoelectrochemical applications require polymers to operate in high-dielectric electrolytes, which modifies their structure and the local dielectric environment, both affecting photophysical processes. To date, it has been difficult to disentangle the impact of the dielectric environment on the optoelectronic response from the one of structure. Here, we address this challenge using solid-state blends of the semiconducting poly(3-hexylthiophone) (P3HT) and the polar, high dielectric poly(vinylidene fluoride) (PVDF). We selected these blends because solution processing at high entanglement densities limits mass transport, forcing the system in a relatively static state where the polar PVDF macromolecules are finely intermixed with the P3HT. This creates a scenario where the P3HT is exposed to a high dielectric environment, emulating the liquid electrolyte: polymer systems commonly used in energy storage/harvesting systems. We find that the chain conformations of the PVDF vary on extremely local length scales (<1 nm), creating a locally heterogeneous dielectric environment. We then compare these local structural variations with the bulk dielectric permittivity. Moreover, the P3HT chains in the blended system adapt a more torsionally ordered conformation, which increases intrachain exciton delocalization. Resolving exciton dynamics in the blends, we then show how both these structural and dielectric effects manipulate charge separation processes. These insights provide mechanistic understanding on the influence of high-polar media on the structure and photophysical processes in polymer semiconductors, towards widely applicable materials guidelines for the design of next-generation polymer semiconductors for energy storage and conversion.

### 15.25. Probing the Effect of Phase Morphology on Electrochemical Doping in a Conjugated Hairy-Rod Polymer

Jude Kpare *, Natalie Stingelin and Henry Kantrow
Georgia Institute of Technology
*  Correspondence: jkpare3@gatech.edu

**Abstract:** Conjugated hairy-rod polymers have emerged as promising materials for energy storage and solar fuel applications, but there is still little understanding of how phase morphology impacts their electrochemical properties. Understanding such relationships is critical for applications where the insertion of counterions and electrolyte species can alter the overall solid-state structure. To systematically probe these interdependencies, we focus on the prototypical conjugated hairy-rod polymer, PBTTT, blended with methyl-ester surfactants to control the polymer’s phase morphology and local ordering. We show that methyl-ester surfactants intercalate within the PBTTT sidechains. Importantly, the PBTTT lamellar spacing can be manipulated to increase, decrease, or stay the same as in the neat material, depending on the surfactant’s alkyl tail length/nature. This structural control promises to open up the opportunity to investigate the electrochemical and photo-physical properties of different solid-state structures of PBTTT. The insights gained from our work may, thus, provide a framework for improved characterization of interphases in hairy-rod polymer:electrolyte systems which is a key challenge for their integration into energy conversion and storage technologies.

### 15.26. Unraveling the Physical Chemistry of Polyolefin-Cellulose Interfaces

**Hsiang-Ju Kai** **^1^** ^,^*, Natalie Stingelin **^1^** **and Thomas Gartner** **^2^**
^1^  Georgia Institute of Technology
^2^  Lehigh University
*  Correspondence: hkai3@gatech.edu

**Abstract:** Composite materials (materials composed of subunits with two or more chemistries) are popular for many industrial applications or consumer products for their beneficial properties such as good mechanical strength and chemical stability. One class of broadly used composite materials are made from a combination of petroleum and renewable sources. As these materials play a significant role in both traditional consumer products (e.g., plastic-coated paper cups) and advanced functional materials (e.g., polyolefin-cellulose nanocrystal nanocomposites) applications, they have garnered engineering interest to investigate methods or pathways to improve not only their material properties but also their recycling/disposal processes. Since these composite materials are often designed to possess strong adhesion between the petroleum-based plastic and renewably-sourced material, from the standpoint of renewable products and environmental sustainability, this desired property renders the end-of-life recycling or upcycling processes difficult, and poses challenges to forest products industry which focuses heavily on using recycled-materials more effectively. To address this issue, here we discuss natural/synthetic polymer adhesion and delamination and apply molecular simulation to investigate the physical chemistry of polyolefin-cellulose interfaces. Experimental methods are used to verify critical findings from the simulations towards novel degradation/deconstruction pathways for more facile recycling/disposal processes.

### 15.27. The Effects of Layer Height on a 3D Printed PLA/TPU/ABS Multi-Material Weld Interface for Simplification of Hybrid Polymer Manufacturing

**David Dabkowski** **^1^** ^,^*, Avery Blau **^1^, Samantha Sedillo** **^1^, Andrew Tchiblakian** **^1^,** **and Rigoberto Advincula** **^2^**
^1^  University of Tennessee
^2^  University of Tennessee—Knoxville
*  Correspondence: ddabkows@vols.utk.edu

**Abstract:** One of the most utilized forms of polymer blends involve a blending procedure that is very labor and energy intensive, often involving a twin screw extruder to create a hybrid filament. Our testing delves into the application of a multi-filament printer utilizing very fine print resolution to enable us to make near homogenous blends without the use of the blending procedure. This will also allow us to tailor the properties of the print on-the-fly instead of creating a large batch of filament each time a new blend is tested. We present our findings of testing this pseudo-homogeneous blend sample for PLA, TPU, ABS hybrid samples. These samples were then mechanically characterized via tensile, flexural, and compression testing and further analyzed with scanning electron microscopy and X-ray diffraction. The presentation will also touch on potential changes in design geometry to further tune the properties.

### 15.28. Detection of Changes in Crystallinity of 3D Printed PLA Using Broadband Dielectric Spectroscopy

**James Lambert** **^1^** ^,^* **, Rigoberto Advincula** **^1^** **and Yangyang Wang** **^2^**
^1^  University of Tennessee—Knoxville
^2^  Oak Ridge National Laboratory
*  Correspondence: jlambe30@vols.utk.edu

**Abstract:** In recent years, 3D printing has made leaps and bounds in the world of polymers. The advancement in technology has allowed various types of polymeric material to be used in applications never thought possible. This work presents techniques which may be used to characterize the dielectric properties of various rigid and flexible 3D printed polymers using broadband dielectric spectroscopy (BDS). A parallel plate geometry was used to test across a range of low to high frequencies, giving insight into the capacitance, permittivity, and dielectric constant of the printed material. Variation of printing parameters allows better control of the crystallinity of 3D printed PLA, with preliminary results showing a 10% shift in permittivity with increasing crystallinity. This technique offers various degrees of tunability, allowing for the application of controlled temperature gradients and atmospheric conditions. The goal of this research is to detect the variations in dielectric properties as a result of changes in 3D printing parameters, with potential applications to a variety of different polymer types and print compositions.

### 15.29. Improving Thermomechanical Properties of DLP 3D-Printed Composites, Using Phosphate Functionalized Graphene at Ultralow Loadings

Jose Bonilla-Cruz *
Research Center for Advanced Materials (CIMAV)
*  Correspondence: jose.bonilla@cimav.edu.mx

**Abstract:** Advanced materials are increasingly demanded in applications such as aerospace equipment, medical devices, and healthcare equipment. Overall, such materials must be lightweight, stiff, and strong to maximize their efficiency while maintaining structural integrity. Owing to the complexity of lattice designs, 3D printing techniques with contactless methods, high speed (up to 15 mm/s), superior scalable resolution (down 1 mm), and low shear forces like digital light processing (DLP) are pursued to obtain versatile and affordable processes. Herein, we obtained DLP 3D-printed composites using ultralow loadings (<0.1 wt.%) of phosphatefunctionalized graphene into a commercial resin (Formlabs elastic resin). We evaluated the effect of the ultralow loads at 0.01, 0.025, and 0.05 wt.% over the thermomechanical macroscopic properties. The functionalized graphene was homogeneously dispersed into isopropyl alcohol via ultrasonication for 10 min. Then, the resulting dispersion was added to the Formlabs elastic resin, which assisted with magnetic stirring for 10 min. The color changed as the functionalized graphene load increased. The insights derived from our study indicate that using phosphate-functionalized graphene nanosheets at ultralow loadings has the potential to modulate the thermomechanical properties of commercial resins, obtaining DLP 3D-printed composites with specific stiffness and strength.

### 15.30. Upcycling of Multi-Layered Plastics through Delamination

Matteo Palesati *, Mark Weber, Naomi Deneke, Blair Brettmann and Natalie Stingelin
Georgia Institute of Technology
*  Correspondence: mpalesati3@gatech.edu

**Abstract:** The recovery of single polymer layers from multi-layer packaging is a crucial step in the upcycling process. However, realizing efficient upcycling has proven challenging due to the complexities of such materials systems. Multi-layer packaging materials are typically composed of different polymer layers (e.g., PE or PP) and an adhesive tie-layer binding them together. Due to the strong adhesion provided by adhesive tie-layers, delamination methods such as peeling are not suitable for multi-layer plastic upcycling. As such, a promising method to achieve delamination for subsequent upcycling relies on diffusing solvents to the adhesive interface between the polymer layers and selectively dissolving the tie-layer. However, despite some existing literature and processes related to solvent-induced delamination, there is still a poor understanding of the kinetics of diffusion through the polymer layers, particularly when solvents are used that also swell the polymer. Here, we present a detailed description of solvent diffusion with the objective of deciphering the role of polymer swelling and diffusion kinetics on solvent transport using Yasuda’s description of free-volume dependent diffusion. This model is compared with experimentally derived swelling coefficient parameters obtained from quartz crystal micro balance with dissipation monitoring (QCM-D) measurements. The combination of the diffusion/swelling model and measured values will provide a platform towards predicting the delamination behavior of multi-layer plastics for a wide range of solvent and polymer/tie-layer combinations, allowing for the maximization of material recovery and the minimization of solvent waste.

### 15.31. Multivariate Approach to Predict Thermal Degradation in Wire and Cable Insulation

Amy Kurr * and David Harper
University of Tennessee—Knoxville
*  Correspondence: ackurr2@gmail.com

**Abstract:** Codes and regulations exist for wire and cable qualification, certification, and use; however, no organization has developed a consensus or standard for when insulation warrants replacement from aging due to thermal exposure, a primary stressor of wire and cables. This knowledge gap threatens public safety, property damage, and business operations. A multivariate approach is presented to investigate the thermo-oxidative degradation of crosslinked polyethylene (XLPE) insulation for application in the automotive field based on different dependent properties.

### 15.32. Kinetic Study of Pyrolysis of Tobacco Particles of Different Sizes Based on the Distributed Activation Energy Model

Wen Kui Zhu *, Gaofei Guo, Ke Zhang, Duoduo Huang and Bin Li
Zhengzhou Tobacco Research Institute of CNTC
*  Correspondence: wkzhu79@r63.com

**Abstract:** Particle size significantly impacts the thermal degradation behavior of biomass during thermochemical conversion. This study examines the thermal degradation behavior of flue-cured tobacco with different particle sizes using a macro-thermogravimetric analyzer (macro-TGA) under inert and oxidative conditions. Under nitrogen, the degradation process has four stages, and under air, it has five stages. The weight loss rate during hemicellulose decomposition increases with decreasing particle size, except for medium cut tobaccos (MCT-0.8, MCT-1.0, and MCT-1.2) under nitrogen. The combustion character index rises with smaller particle size and is lower for cut tobaccos compared to tobacco powders. Kinetic parameters show that activation energy varies with conversion rate. Under nitrogen, activation energy increased with conversion rate for pyrolysis, while under air, it peaked at a middle conversion rate for combustion. Additionally, SCT-1.0 has the highest, and P-MCT-1.0 the lowest average activation energy.

### 15.33. Electrophoretically Deposited TiO_2_-Containing Pectin Smart and Corrosion-Resistant Composite Coatings

Marcel Roy Domalanta *, Mark Rigel Ali and Eugene Caldona
North Dakota State University
*  Correspondence: marcel.domalanta@ndsu.edu

**Abstract:** Stimuli-responsive coatings, with reversible tunable wettability, are highly attractive for a broad range of biological, chemical, environmental, and electrical applications. Titania (TiO_2_) has been at the forefront of industrial-scale competency as an additive for coating applications due to its high-index light scattering and photo-responsivity—characteristics essential for fabricating smart coatings. However, the methods used in further improving its durability and compatibility, generally involve the utility of rare earth metals coupled with complex fabrication approaches. As such, efforts have pushed through in adapting sustainably sourced materials to synergize with TiO_2_, including the use of chitosan, cellulose, pectin, and the like. In this study, facile electrophoretic deposition from ethanol-water solution was employed to fabricate novel TiO_2_-containing pectin composite coatings, with switchable wettability, for corrosion protection applications. The influence of varying TiO_2_ loadings in a predetermined pectin concentration on the structural and topographic properties was investigated by spectroscopy, microscopy, and diffraction technique, while wetting reversibility was studied by contact angle measurements before and after UV light exposure. Furthermore, electrochemical impedance spectroscopy, potentiodynamic polarization, and electrochemical microscopy were used to assess the coatings’ corrosion protection properties and occurrence of localized corrosion.

### 15.34. Super-Nonwettable and Superoleophilic Fluoropolymer-Modified Electrodeposited Polythiophene Coatings for Corrosion Protection

Mark Rigel Ali *, Jackson Honeyman, Marcel Roy Domalanta and Eugene Caldona
North Dakota State University
*  Correspondence: mark.ali@ndsu.edu

**Abstract:** In this study, we introduce an approach aimed at enhancing the adhesion, surface stability, water repellency, and corrosion resistance of electrodeposited polythiophene (PTH) coatings by integrating a thienyl-substituted silane coupling agent as a surface modifier alongside poly(vinylidene fluoride-co-hexafluoropropylene) as a topcoat. The fluoropolymer inclusion exploits its inherent hydrophobicity to further improve the resultant coating’s water repellency, while the utility of a silane coupling agent addresses a crucial yet often overlooked in polymeric coatings—ensuring strong adhesion to steel substrates. Spectroscopic analyses confirm the silanization of steel, indicating strong chemical bonding between the substrate and coating. Subsequent tape adhesion tests substantiate this finding, revealing minimal coating delamination and corroborating the enhanced adhesion achieved. Even after rigorous cyclic corrosion tests, the coating maintains a consistently high water contact angle (~163°), demonstrating remarkable stability and durability, while impedance measurements and potentiodynamic polarization unveil a notable increased corrosion resistance (low frequency impedance modulus |Z|0.1 Hz ~7.2 mΩ cm^2^, corrosion potential ECORR ~0.14 V, and corrosion current ICORR ~0.76 nA). Our approach, unprecedented in existing literature, not only extends the lifespan of PTH coatings, but also broadens their utility in corrosion-prone environments. Overall, this study sets a new benchmark for the development of durable and hydrophobic protective coatings, offering a promising avenue for future research and industrial applications.

### 15.35. The Use of Thermal Analysis and Calorimetry for the Design and Production of Thermite-Based Heaters for Plug and Abandonment in Oil and Gas

Andre Levchenko *, Paul Carragher and Arild Stein
BiSN Oil Tools LLC
*  Correspondence: andrelevko@hotmail.com

**Abstract:** Setting cement plugs is a traditional way of abandoning an oil or gas well at the end of its lifespan [1]. However, the cement plugs tend to crack and leak over time and are corroded by carbon dioxide present in the wells. A resurgence of practical interest in carbon capture and sequestration has brought corrosion-resistant bismuth-based plugs to the forefront of research and development in the oil and gas industry [2]. Bismuth-based plugs not only expand on solidification creating a gas tight seal with the well casing material but also resist corrosion in the harshest environments of high-pressure carbon dioxide and hydrogen sulfide [3,4]. To set a bismuth-based plug downhole in a range of geometries, a high energy heat source is required to melt the plug material. A thermite reaction is used in BiSN heaters which are, in simple terms, sealed pipes loaded with a “tamed” thermite. Designing such heaters and deploying them downhole are the art forms requiring knowledge of thermite burn characteristics including the energy output and burn speeds.

Varying the average particle size (and distribution) as well as using a binding agent are important methods to adjust the burn characteristics. Adding other inert components can also alter the burn speed and energy output. In this presentation, thermal analysis and calorimetry are shown as useful tools to characterize the thermite materials based on common components such as aluminum fuel and iron oxides. We present the data demonstrating how thermal analysis and calorimetry are utilized in our efforts to develop a well-behaved thermite composition for a wide range of novel commercial/practical applications and conduct quality control in the manufacturing process.

**References**

1. Vrålstad, T.; Saasen, A.; Fjær, E.; Øia, T.; Ytrehus, J.D.; Khalifeh, M. Plug & abandonment of offshore wells: Ensuring long-term well integrity and cost-efficiency. *J. Pet. Sci. Eng.* **2019**, *173*, 478–491. https://doi.org/10.1016/j.petrol.2018.10.049.
2. Ars, F.; Rios, R. Decommissioning: A Call for a New Approach. In Proceedings of the Offshore Technology Conference, Houston, Texas, USA, 1–4 May 2017. https://doi.org/10.4043/27717-MS.
3. Carragher, P.; Fulks, J. A New Look at Sealing with Bismuth and Thermite. In Proceedings of the SPE Annual Technical Conference and Exhibition? Dallas, TX, USA, 24–26 September 2018. https://doi.org/10.2118/191469-MS.
4. Fulks, J.M.; Carragher, P. A New Solution for Well Abandonment: Bismuth and Thermite. In Proceedings of the Offshore Technology Conference Asia, Kuala Lumpur, Malaysia, 2–6 November 2020. https://doi.org/10.4043/30421-MS.

